# Selective targeting of collagen IV in the cancer cell microenvironment reduces tumor burden

**DOI:** 10.18632/oncotarget.24280

**Published:** 2018-01-19

**Authors:** Fernando Revert, Francisco Revert-Ros, Raül Blasco, Aida Artigot, Ernesto López-Pascual, Roberto Gozalbo-Rovira, Ignacio Ventura, Elain Gutiérrez-Carbonell, Nuria Roda, Daniel Ruíz-Sanchis, Jerónimo Forteza, Javier Alcácer, Alejandra Pérez-Sastre, Ana Díaz, Enrique Pérez-Payá, Juan F. Sanz-Cervera, Juan Saus

**Affiliations:** ^1^ FibroStatin, Parc Científic Universitat de València, Paterna 46980, Spain; ^2^ Centro de Investigación Príncipe Felipe, Valencia 46012, Spain; ^3^ Departament de Química Orgànica, Universitat de València, Burjassot 46100, Spain; ^4^ Departament de Bioquímica i Biologia Molecular, Universitat de València, Burjassot 46100, Spain; ^5^ Instituto Valenciano de Patología of Universidad Católica de Valencia, Centro de Investigación Príncipe Felipe, Valencia 46012, Spain; ^6^ Anatomía Patológica, Hospital Quirónsalud de Valencia, Valencia 46010, Spain; ^7^ Unitat Central d'investigació en Medicina, Facultat de Medicina i Odontologia, Universitat de València, Valencia 46010, Spain; ^8^ Present address: Departament de Microbiologia i Ecologia, Facultat de Medicina i Odontologia, Universitat de València, Valencia 46010, Spain; ^9^ Present address: Universidad Católica de Valencia, Valencia 46001, Spain; ^10^ Present address: Sciex Spain, Alcobendas, Madrid 28108, Spain; ^11^ Present address: Sistemas Genómicos, Paterna 46980, Spain

**Keywords:** GPBP, collagen IV, EMT, drug-resistant cancer, tumor microenvironment

## Abstract

Goodpasture antigen-binding protein (GPBP) is an exportable^1^ Ser/Thr kinase that induces collagen IV expansion and has been associated with chemoresistance following epithelial-to-mesenchymal transition (EMT). Here we demonstrate that cancer EMT phenotypes secrete GPBP (mesenchymal GPBP) which displays a predominant multimeric oligomerization and directs the formation of previously unrecognized mesh collagen IV networks (mesenchymal collagen IV). Yeast two-hybrid (YTH) system was used to identify a ^260^SHCIE^264^ motif critical for multimeric GPBP assembly which then facilitated design of a series of potential peptidomimetics. The compound 3-[4''-methoxy-3,2'-dimethyl-(1,1';4',1'')terphenyl-2''-yl]propionic acid, or T12, specifically targets mesenchymal GPBP and disturbs its multimerization without affecting kinase catalytic site. Importantly, T12 reduces growth and metastases of tumors populated by EMT phenotypes. Moreover, low-dose doxorubicin sensitizes epithelial cancer precursor cells to T12, thereby further reducing tumor load. Given that T12 targets the pathogenic mesenchymal GPBP, it does not bind significantly to normal tissues and therapeutic dosing was not associated with toxicity. T12 is a first-in-class drug candidate to treat cancer by selectively targeting the collagen IV of the tumor cell microenvironment.

## INTRODUCTION

Goodpasture disease is a rare, human-specific autoimmune disorder mediated by circulating antibodies against the non-collagenous 1 (NC1) domain of the α3 chain of collagen IV (α3NC1) [1]. A unique region in the human α3NC1 is the target of GPBP kinase which is active at the low extracellular levels of ATP [2–4]. GPBP (also known as GPBP-1 or 77-kDa GPBP), the primary product of *COL4A3BP*, is a polypeptide with a M_*r*_ ranging from 71- to 89-kDa mainly depending on phosphoresidue content [3] which assembles into two major quaternary structures, one trimeric and the other multimeric exhibiting higher autophosphorylation activity [5]. Two other isoforms are generated from the *COL4A3BP* gene: GPBP-2 (also known as GPBPΔ26 and ceramide transfer protein or CERT), an alternative splice variant which transfers ceramide between the endoplasmic reticulum (ER) and Golgi apparatus and promotes protein secretion [5–7]; and GPBP-3 (also known as 91-kDa GPBP), a variant arising from alternative translation initiation containing additional N terminal sequence for membrane binding which facilitates GPBP exportation [4].

Collagen IV is a principal component of epithelial basement membranes where it exists in sheet-like network referred to here as epithelial or membrane collagen IV [8]. It also exists in more expanded mesh-like organization in the renal glomerulus [9], and has been associated with decidua formation [10] and pancreatic cancer malignancy [11]. Mesh-like collagen IV is produced by cells of mesenchymal lineage (i.e. mesangial and epithelioid cells) and possibly by cancer cells that have undergone EMT and is referred to here as mesenchymal or mesh collagen IV. In the renal glomerulus, increased extracellular GPBP induces membrane collagen IV disruption and mesh collagen IV expansion [12]. Increased peri-cellular GPBP expression precedes mesh collagen IV emergence in decidua formation [10]. These observations suggest that exportable GPBP directs mesenchymal collagen IV meshwork formation.

In basement membranes, the α1-α6 chains of collagen IV form three different triple helical molecules (protomers). The (α1)_2_α2 combination is the most commonly expressed whereas α3α4α5 and (α5)_2_α6 are present only in specific tissues [8]. Mesh collagen IV is composed predominantly of α1 and α2 chains [9, 10], but their precise molecular and supramolecular organizations have not yet been established.

*COL4A3BP* activation has been associated with multidrug resistance [13], a condition attributed to EMT and stemness [14–16], suggesting that GPBP-induced mesh collagen IV maintains EMT phenotypes (progenitor and mesenchymal) populating tumors after chemotherapy. We rationally designed T12 to specifically target the multimeric assembly of extracellular GPBP secreted by the EMT phenotypes. T12 inhibited the formation of two independent mesh collagen IV networks maintaining these phenotypes, one for progenitor based on α1α2(IV) chains and another for mesenchymal containing α5(IV) chain. T12 also exhibited efficacy in treating lung and breast cancer in mouse models without apparent toxicity.

## RESULTS

### GPBP directs the formation of two independent mesh collagen IV networks in chemoresistant EMT cancer cell phenotypes

**Figure 1:**
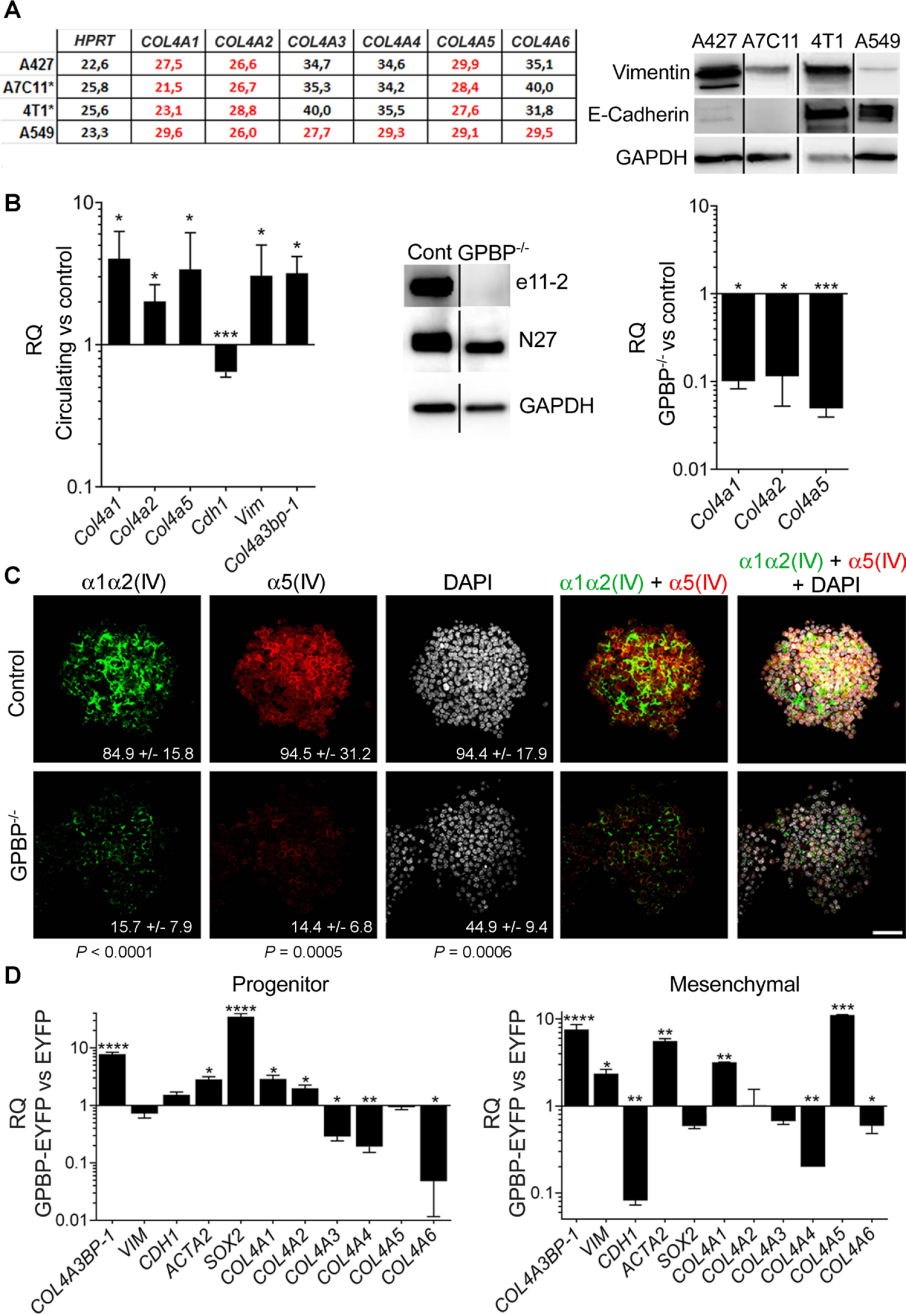
GPBP directs the formation of two independent mesh collagen IV networks in chemoresistant EMT cancer cell phenotypes (**A**) *Left*, the threshold-cycle (C_T_) for the indicated mRNA and cell lines was determined by RT-qPCR. Denoted in red characters are predominantly expressed mRNA (^*^murine breast cancer cell lines). *Right*, WB analysis of the indicated cell cultures and polypeptides. (**B**) *Left*, represented with bars are the relative quantity (RQ) expressed as mean ± standard deviation (SD) of the indicated mRNA in c4T1 cultures (circulating) in comparison (*versus*, vs) with 4T1 cultures (control). *Middle*, WB analysis of the indicated 4T1 cell extracts to assess GPBP (e11-2), GPBP and GPBP-2/CERT (N27) and GAPDH (glyceraldehyde 3-phosphate dehydrogenase) expression. *Right*, RQ of the indicated mRNA and 4T1 cultures is represented as at *left.* Statistics: Student’s *t-*test*, n* = 2. (**C**) The indicated 4T1 spheroids and polypeptides were analyzed by IF-CM. In this and following figures denoted in arbitrary units (AU) are fluorescence intensities (FI) expressed as mean ± SD of five representative fields (*n* = 5). Statistics: Student’s *t*-test. (**D**) RQ of the indicated mRNA is represented as in B comparing A549-derived clone C79 (progenitor) or G7A (mesenchymal) expressing GPBP-EYFP with an A549 clone expressing EYFP (EYFP). Shown is a representative analysis of two independent experiments. Statistics: Student’s *t*-test, *n* = 2. In all the figures: n, sample size per group; ^*^P < 0.05, ^**^P < 0.01, ^***^P < 0.001, ^****^P < 0.0001; DAPI (4′,6-diamidine-2′-phenylindole dihydrochloride) stained cell nucleus; WCIF ImageJ was used for quantification of WB; unless otherwise indicated scale bars were 50 μm.

Reverse transcription coupled to quantitative polymerase chain reaction (RT-qPCR) of purified RNA was used to analyze collagen IV gene expression in several carcinoma cell lines, including human non-small cell lung carcinoma (NSCLC) A427 and A459 and murine breast carcinoma A7C11 and 4T1. Intriguingly, A427, A7C11 and 4T1 expressed *COL4A1/Col4a1, COL4A2/Col4a2* and *COL4A5/Col4a5,* coding for previously unrecognized α1α2α5(IV) chain combination whereas A549 cells expressed *COL4A1*-*COL4A6*, coding for α1-α6(IV) chains. Western blot (WB) analysis revealed that cell lines expressing *COL4A1/Col4a1, COL4A2/Col4a2* and *COL4A5/Col4a5* combination expressed relatively more vimentin (intermediate filament characteristic of mesenchymal cells) than A549 cells which expressed abundant E-cadherin known to stabilize epithelial organization (Figure 1A), suggesting that cancer cell lines expressing α1α2α5(IV) chain combination have underwent EMT [17]. Further, 4T1 cells were seeded in mammary path of Balb/c female mice and, following xenograft formation, circulating 4T1 (c4T1), representing more developed EMT phenotypes mediating metastases formation, were isolated and in-parallel cultured with primary 4T1 cells (Figure 1B, left). As expected c4T1 cultures exhibited a more mesenchymalized phenotype and expressed less *Cdh1*, coding for E-cadherin, and more *Vim*, coding for vimentin, than cultures of primary 4T1 cells. Importantly, c4T1 cultures expressed more *Col4a1, Col4a2* and *Col4a5,* and *Col4a3bp-1*, coding for GPBP, suggesting that EMT phenotypes expressed GPBP-dependent α1α2α5(IV) chain combination. Consistently, CRISPR/Cas9 edited 4T1 cells (Supplementary Figure 1A–1C) lacking GPBP (GPBP^−/−^) but producing non-exportable GPBP-2/CERT (Figure 1B, middle) expressed *Col4a1/Col4a2/Col4a5* at low levels (Figure 1B, right). Subsequently, 4T1 and 4T1 GPBP^−/−^ cells were cultured in three-dimensional (3D) conditions and the resulting spheroids analyzed by immunofluorescence and confocal microscopy (IF-CM) (Figure 1C). These studies revealed the existence of a previously unrecognized GPBP-dependent collagen IV network containing the α5(IV) chain which assembled independently from the α1α2(IV) chains, and also displayed GPBP-dependent meshwork organization. To further investigate GPBP relevance in EMT we forced constitutive GPBP expression in A549 cells (Figure 1D). Clones exhibiting progenitor phenotype (elevated *SOX2*, coding transcriptional factor that maintains pluripotency) expressed predominantly *COL4A1* and *COL4A2*. Clones exhibiting predominant mesenchymal phenotype (reduced *CDH1*, elevated *VIM* and *ACTA2*, coding for α-smooth muscle actin -α-SMA- which renders cells migratory) expressed predominantly *COL4A5.* Clones expressing a mixed phenotype were also found (Supplementary Figure 1D). These data suggested that GPBP overexpression had different consequences on individual phenotypes present in A549 cultures (see below) and induced two distinct mesh collagen IV networks: α1α2(IV) for progenitor and α5(IV) for mesenchymal phenotype maintenance. The notion that α5(IV) network maintains more differentiated mesenchymal phenotypes was further supported by demonstrating that a murine leukemia RAW 264.7 macrophages expressed only α5(IV) network (Supplementary Figure 1E).

### GPBP and mesh collagen IV networks maintain chemoresistant EMT cancer cell phenotypes in A549 spheroids

**Figure 2:**
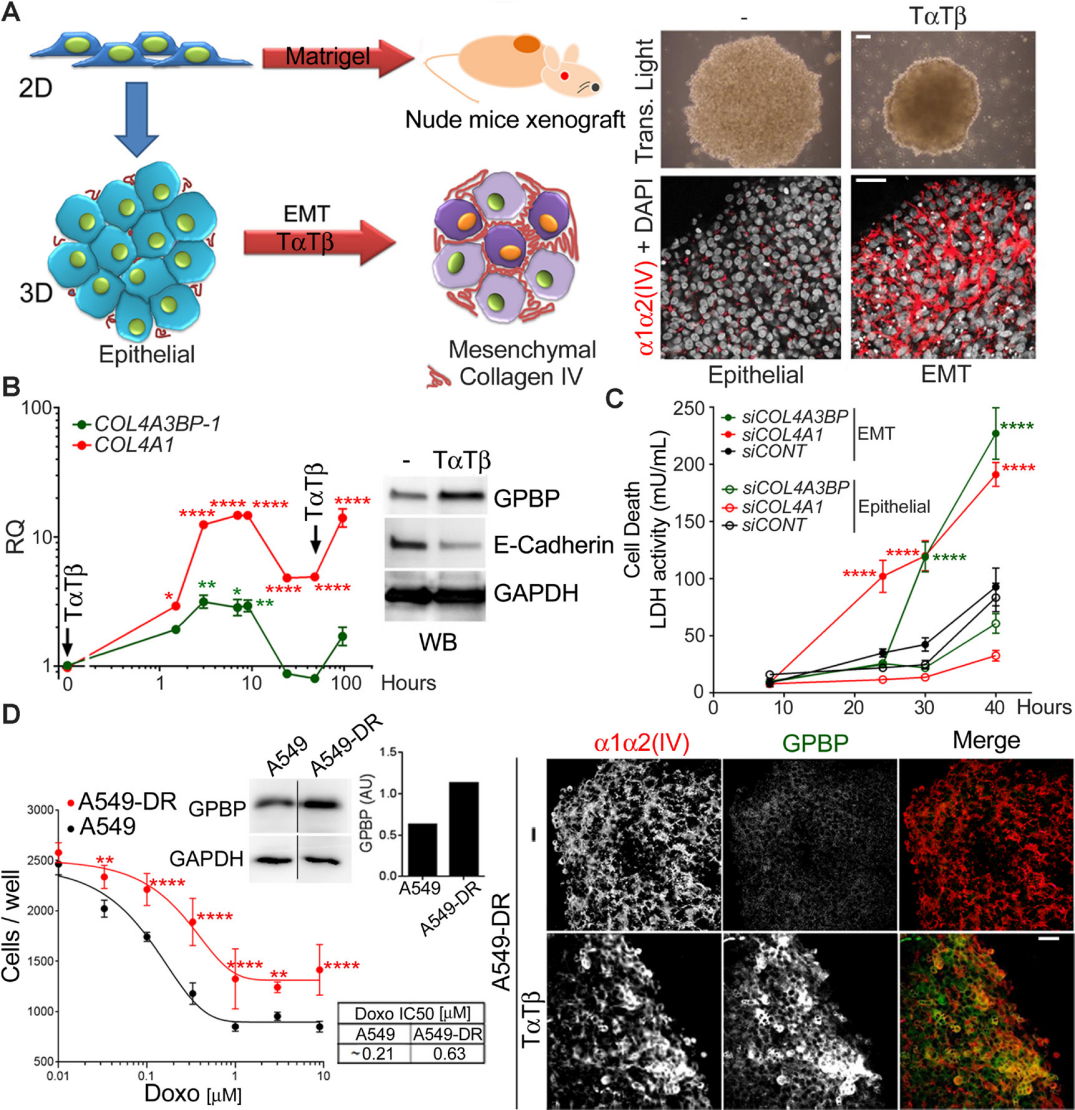
GPBP and mesh collagen IV networks maintain chemoresistant EMT cancer cell phenotypes in A549 spheroids (**A**) *Left,* A549 2D cultures form tumors in nude mice when inoculated with Matrigel^®^ or spheroids when seeded in non-adherent plates (3D culture conditions). Spheroids exhibit *epithelial* characteristics or, upon EMT-induction with TαTβ, they display an *EMT* phenotype. *Right*, spheroids observed by transmitted light microcopy (top, bar = 200 μm) or IF-CM with antibodies against the indicated polypeptides. (**B**) *Graph*, plotted is RQ of indicated mRNA over time in stimulated vs unstimulated A549 spheroids. Arrows denote stimuli pulses. *Image*, WB analysis of the indicated spheroids (24 h) and polypeptides. Statistics: one-way ANOVA and Dunnett’s (1WA-D) comparison respect *t* = 0 in each series. Results are expressed as mean ± SD of three assays. (**C**) A549 cultures were transfected with the indicated small interfering (si)RNA (siCONT = control siRNA) seeded in 3D conditions and following TαTβ stimulation, lactate dehydrogenase (LDH) measured over time in culture media (mean ± SD). A siRNA efficacy >75% was determined by RT-qPCR at the end of the studies. Statistics: 2WA-Sidak’s (2WA-S), *n* = 2. (**D**) *Left*, dose-response curve (mean ± SD) (graph) and IC50 (table) of doxorubicin, and GAPDH-normalized GPBP expression (WB and histogram) were determined for the indicated cultures. *Right*, IF-CM analysis of indicated spheroids and polypeptides. Statistics: 2WA-S, *n* = 4. Shown are representative analysis of a total of three (A, D) and two (C) assays.

To determine the relevance of GPBP in EMT induction, we used A549 cells reported to undergo EMT in response to tumor necrosis factor-alpha (TNF-α) and transforming growth factor-beta (TGF-β) [18], referred to here as TαTβ stimulation. Due to its expanded side population (SP) containing progenitor phenotypes [19], A549 cells are prompt to form spheroids in 3D culture conditions and tumors (xenografts) in nude mice (Figure 2A, left). Compared with two-dimensional (2D) cultures, A549 spheroids expressed relatively more *CDH1*, *ACTA2*, *SOX2* and *NANOG*, the latter also coding for a transcriptional factor that maintains pluripotency (Supplementary Figure 2A). Thus, the evidence revealed that A549 progenitor compartment expanded further under 3D cultures conditions (epithelial spheroid). TαTβ stimulation of epithelial spheroids upregulated *VIM*, downregulated *CDH1*, and further activated *NANOG* and *SOX2* (Supplementary Figure 2B) previously associated with EMT in human NSCLC [20]. Resulting EMT spheroids expressed more *COL4A3BP-1, COL4A1* and *COL4A2* than epithelial spheroids but not more *COL4A5*, suggesting that TαTβ stimulation induced predominant epithelial progenitor cells populating epithelial spheroids to switch into mesenchymal progenitor phenotypes. EMT spheroids were more sclerotic due, at least in part, to an enlarged α1α2(IV) network exhibiting mesh-like organization (Figure 2A, right). mRNA expression monitoring revealed that *COL4A3BP-1* and *COL4A1* were coordinately regulated during EMT induction and WB analyses further revealed that EMT phenotypes expressed more GPBP (Figure 2B). Altogether, these data suggested that GPBP and α1α2(IV) mesh collagen IV induced and maintained mesenchymal progenitor phenotypes. Indeed, down-regulation of either *COL4A3BP* or *COL4A1* compromised the viability of EMT and not of epithelial A549 spheroids (Figure 2C). Finally, we generated a population of doxorubicin-resistant A549 cells (A549-DR) that were cultured in 3D conditions and stimulated with TαTβ (Figure 2D). A549-DR expressed more GPBP and formed spheroids with a prominent mesh collagen IV network which accumulated GPBP following TαTβ stimulation. Collectively, the data suggested that exportable GPBP and resulting mesh collagen IV network induced and maintained A549 drug-resistant EMT phenotypes.

### GPBP and mesh collagen IV networks associate with EMT phenotypes in human NSCLC

**Figure 3:**
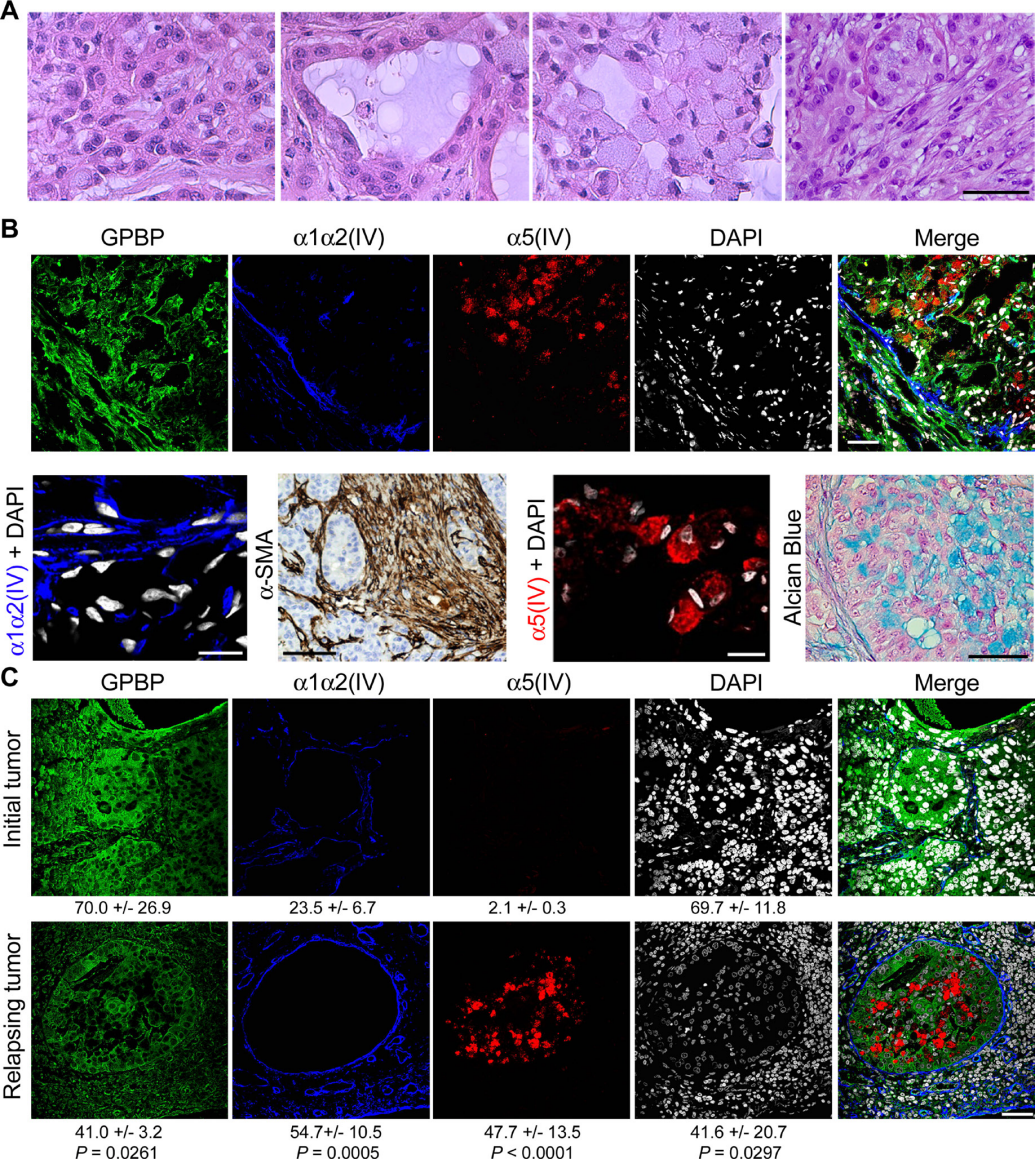
GPBP and mesh collagen IV networks associate with EMT phenotypes in human NSCLC (**A**) HE analysis of cell types in small A549 tumors. Left to right: poorly differentiated, epithelial, signet ring-like and fusiform cells. (**B**) Frozen (IF-CM) and paraffin-embedded (α-SMA and Alcian Blue) sections of small A549 tumors were analyzed. *Upper*, IF-CM detection of the indicated polypeptides in stromal and nodular tumor regions. *Lower*, from left to right the indicated polypeptides were detected by IF-CM (bar = 10 μm), horseradish peroxidase immunohistochemical method (brown) (bar = 100 μm) in tumor peri-nodular stroma, IF-CM (bar = 10 μm), and Alcian blue staining (bar = 20 μm) of the mucus (blue) in tumor nodular regions. (**C**) Paraffin-embedded sections of a NSCLC patient tumor at diagnosis prior to initiation of chemotherapy (Initial tumor) and at relapsing after surgery and chemotherapy (Relapsing tumor) were analyzed by IF-CM to visualize the indicated proteins. Indicated are FI measured, expressed and compared as in Figure 1. Statistics: Student’s *t*-test. Similar conclusions were obtained when comparing untreated *vs* after treatment chemoresistant NSCLC specimens.

To further investigate mesh collagen IV relevance *in vivo*, we analyzed small A549 xenografts as they contain more cells with EMT phenotypes as compared to large xenografts (see below). Hematoxylin eosin (HE) staining revealed four major cell types: poorly differentiated, epithelial, signet ring-like, and myofibroblast-like fusiform (Figure 3A). Tumor nodular regions and surrounding stroma expressed abundant collagenous material (Supplementary Figure 3A) and GPBP (Figure 3B), suggesting that they contained GPBP-dependent EMT phenotypes. But, while the stroma contained fusiform cells expressing α-SMA and mesh α1α2(IV) collagen, revealing their mesenchymal progenitor condition; signet ring-like cells at nodular regions expressed mesh α5(IV) collagen and accumulated mucus (Alcian Blue), revealing they were mesenchymalized epithelial cells that had lost apical-basal polarization and ability for mucus secretion (Figure 3B). We similarly analyzed a naturally occurring human NSCLC tumor at diagnosis and, after chemotherapy and surgery, the corresponding relapsing tumor. Masson staining revealed relapsing tumor to contain more collagenous material than tumor at diagnosis (Supplementary Figure 3B). IF-CM analysis further revealed that naturally occurring NSCLC tumors expressed abundant GPBP in tumor structures resembling nodular regions in A549 xenografts (Figure 3C). However, GPBP distributed more intracellularly in the untreated than in the relapsing tumor where it exhibited a more peripheral extracellular localization. These analyses also revealed two independent mesh α1α2(IV) and α5(IV) networks in these tumor areas. The untreated tumor exhibited a membrane-like α1α2(IV) collagen network encircling epithelial acinar structures virtually devoid of α5(IV) collagen network. In contrast, in the relapsing tumor the α1α2(IV) collagen was significantly expanded displaying mesh-like organization, and the cells occupying the nodular tumor areas expressed abundant mesh α5(IV) collagen. Collectively, the data suggested that both, fusiform cells remaining at the peripheral stroma and signet ring-like cells which detach and occupy the acinar space, derived from epithelial structures undergoing EMT. However, whereas fusiform cells were maintained by an α1α2(IV) meshwork, an α5(IV) meshwork maintained signet ring-like cells. The evidence also suggested that fusiform and signet ring-like cells represent GPBP-dependent EMT phenotypes likely deriving from “epithelial progenitor” and “epithelial secretory” cells, respectively, both residing in the tumor epithelial structures. Finally, data from naturally occurring NSCLC suggest that chemoresistance in the clinical setting is due, at least in part, to a GPBP-dependent EMT.

### The druggable GPBP isoform is a multimer stabilized by self-interacting ^260^SHCIE^264^ motif

**Figure 4:**
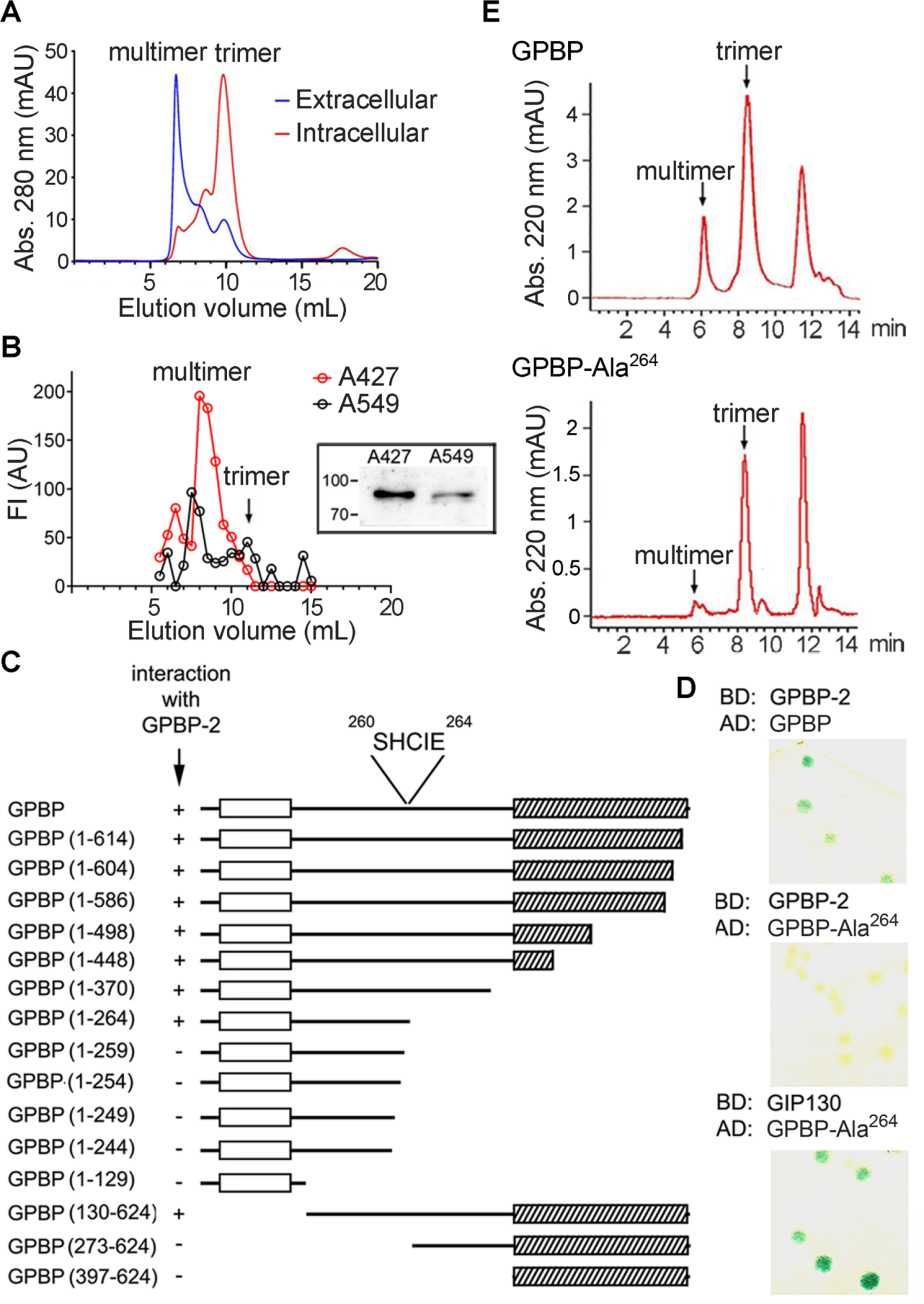
The druggable GPBP isoform is a multimer stabilized by self-interacting ^260^SHCIE^264^ motif (**A**) The indicated recombinant GPBP pools were analyzed by fast protein liquid chromatography (FPLC)-SEC. Calibrators, MW (kDa) and elution volumes (mL) were: thyroglobulin (669, 6.86), aldolase (158, 12.35) and serum human albumin (66, 13.89). In all chromatograms, the positions of the main GPBP oligomers are denoted. (**B**) Media (2.5 L) from the indicated cultures were affinity-purified using single-chain variable fragment (scFv)N26-column. Bound material was eluted in native conditions using N26-competing synthetic peptide (PS132) and analyzed as in A. Represented in AU are the FI of individual fractions in indirect ELISA analysis using anti-GPBP monoclonal antibodies (mAb). Shown is a representative assay of three. *Image*, mAb N27-developed WB of similarly purified (25 mL) GPBP eluted under denaturing conditions. Numbers and bars here and in following WB denote kDa and position of MW markers, respectively. (**C**) Structural features such as PH (empty box) and Start (scratched box) domains are indicated and represent GPBP and deletion mutants thereof. Indicated are spanning residues (numbers) and YTH interaction using colony-lift filter assays (+ or −). The core interactive motif and its position are denoted. (**D**) The interaction of the indicated pairs of fused polypeptides (BD, binding domain; AD, activation domain of GAL4 transcription factor) was assayed as in C (GIP130, GPBP-interacting protein 130-kDa, residues 86–350). Blue color (+). (**E**) The indicated FLAG-tagged polypeptides were expressed, purified and analyzed by high-performance liquid chromatography (HPLC)-SEC. (A, E) Chromatograms are representative of at least three independent experiments.

We previously described intracellular human recombinant GPBP to be mainly trimeric [5]. However, the aggregate status of extracellular GPBP was unknown. Using FLAG-tagged GPBP expression in Sf9 cells, we observed that extracellular GPBP appeared to be mainly multimeric (Figure 4A). This was confirmed with GPBP isolated from the culture media of human NSCLC cells which further revealed that A427, exhibiting EMT phenotype, secreted more GPBP than A549 displaying epithelial phenotype (Figure 4B). A YTH approach was then used to map motifs that stabilize GPBP multimers. Since GPBP exhibited transcriptional activity when fused to the binding domain of GAL4 [5], we used GPBP-2/CERT as bait in these studies. By assaying GPBP deletion mutants, we identified the five-residue motif ^260^SHCIE^264^ to be essential for interacting with GPBP-2/CERT (Figure 4C). Subsequently, we generated Ala mutants of specific individual residues and performed additional YTH assays. GPBP-Ala^264^ did not interact with GPBP-2/CERT (Figure 4D). In contrast, GPBP-Ala^264^ interacted with a polypeptide representing GIP130, an intracellular GPBP substrate [21], suggesting that Glu^264^ was essential for GPBP self-interaction and not for interacting with protein substrates. To further assess the relevance of Glu^264^ in multimer stabilization, we performed comparative size exclusion chromatography (SEC) of purified yeast recombinant GPBP and GPBP-Ala^264^ (Figure 4E). The mutant was mainly trimeric along with traces of higher and lower MW materials, revealing that Glu^264^ was a critical residue for multimer assembly. Finally, Q2 (Ac-LATLSHCIELMVKR-NH2), a synthetic peptide representing α-helix region predicted to contain the interactive motif was shown to hamper GPBP autophosphorylation (Supplementary Figure 4).

### T12, a peptidomimetic of the GPBP region comprising ^260^SHCIE^264^ motif disturbs multimerization

**Figure 5:**
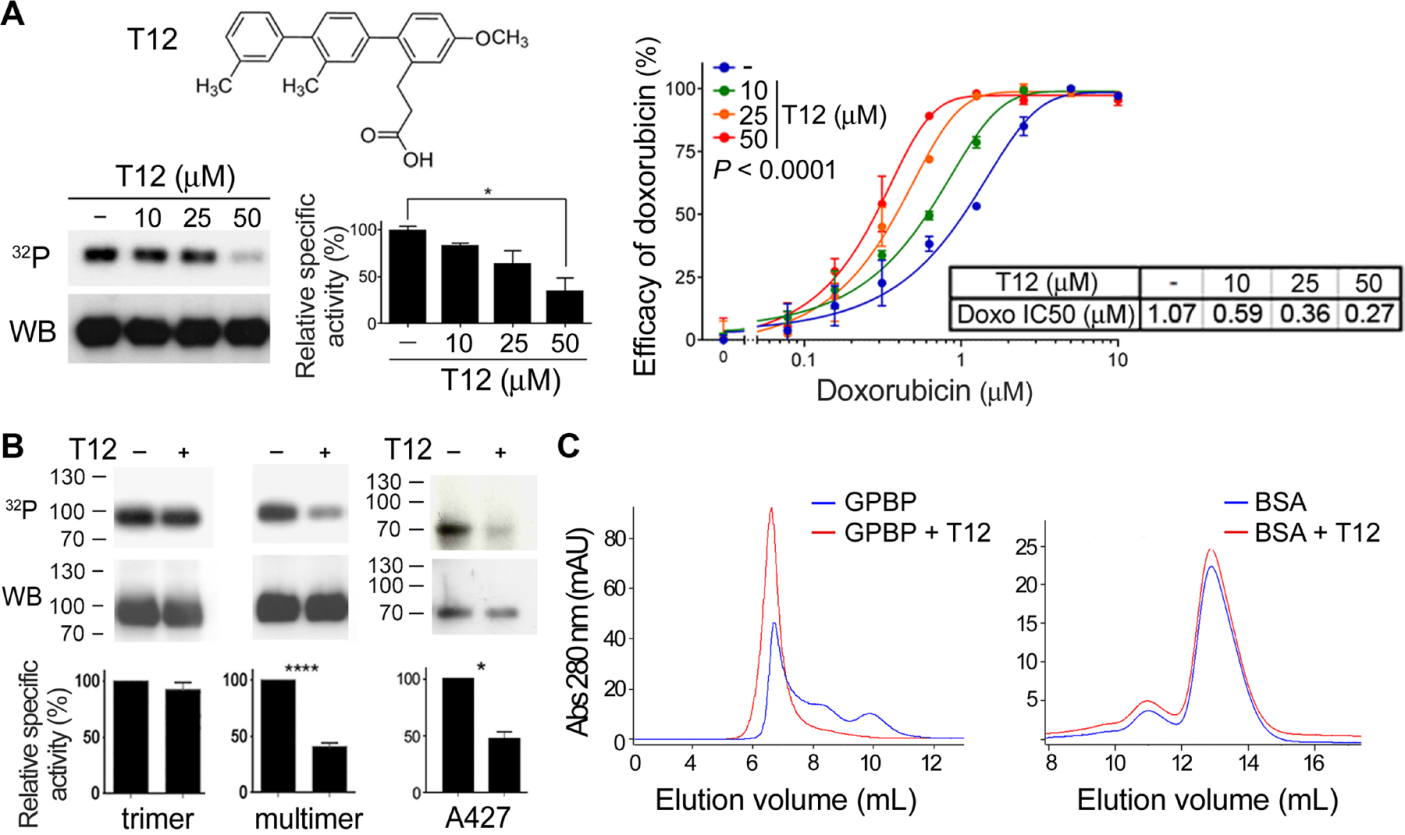
T12, a peptidomimetic of the GPBP region comprising ^260^SHCIE^264^ motif disturbs multimerization (**A**) Chemical structure of T12 and dose-response effects on GPBP autophosphorylation and A549 doxorubicin IC50. Phosphorylation mixtures were analyzed by sodium dodecyl sulfate polyacrylamide gel electrophoresis (SDS-PAGE), blotted and exposed (^32^P) prior to detect GPBP with N27 (WB). Specific activities (histogram) were calculated using WCIF ImageJ and represented as percentage (mean ± standard error of the mean, SEM) of control mixture (−) that was set at 100%. Statistics: 1WA and Fisher’s (1WA-F), *n* = 2. Graph and Table show the efficacy and IC50 of doxorubicin on A549 cultures at the indicated T12 concentrations. Efficacy values are mean ± SD. The 2WA and Tukey’s (2WA-T) and IC50 are indicated, *n* = 4. Representative of five assays. (**B**) Recombinant (multimer or trimer, *n* = 3) or extracellular native (A427, *n* = 2) GPBP were analyzed as in A at 50 µM T12. Statistics: Student’s *t*-test. (**C**) Similar amounts (125 μg) of recombinant extracellular GPBP and bovine serum albumin (BSA) were exposed to T12 (10 mM) during 15 min at room temperature and further FPLC-SEC analyzed essentially as in previous Figure. Represented is one out of three independent analysis performed from two different GPBP preparations.

It has been proposed that peptides forming an α-helix could be mimicked by a terphenyl scaffold in which each phenyl ring contained a different functional group representing the lateral chain of specific residues in the target sequence [22]. To test this, we synthesized terphenyl mimicking Q2 (Supplementary Figure 5A) and a library of related compounds (Supplementary Table 1).^2^ We predicted that potential drug candidates would inhibit GPBP and reduce EMT chemoresistance. Thus, to select a candidate, we tested individual terphenyls for their capacity to reduce GPBP autophosphorylation (*in vitro*) and half maximal inhibitory concentration (IC50) of doxorubicin (*ex vivo*) to similar extent (Supplementary Figure 5B). Terphenyl 12 (T12) or 3-[4″-methoxy-3,2′-dimethyl-(1,1′;4′,1″)terphenyl-2″-yl]propionic acid displayed the best combined activities and was selected for subsequent studies (Figure 5A). T12 reduced autophosphorylation of recombinant multimer but did not affect the recombinant GPBP trimer autophosphorylation activity (Figure 5B). In addition, T12 inhibited to a similar extent native extracellular A427 GPBP mainly consisting of multimeric aggregated (Figure 5B). Finally, at molar GPBP: T12 ratios used in autophosphorylation inhibition assays, T12 induced extracellular GPBP oligomers to form aggregates that eluted at the void volume of the SEC column; however, the SEC profile of an extracellular circulating protein of similar size used as a control was unaffected (Figure 5C). Collectively, the data suggested that T12 disturbs normal GPBP multimer formation without inhibiting kinase active site.

### T12, selectively targets mesenchymal GPBP produced by cancer EMT phenotypes

**Figure 6:**
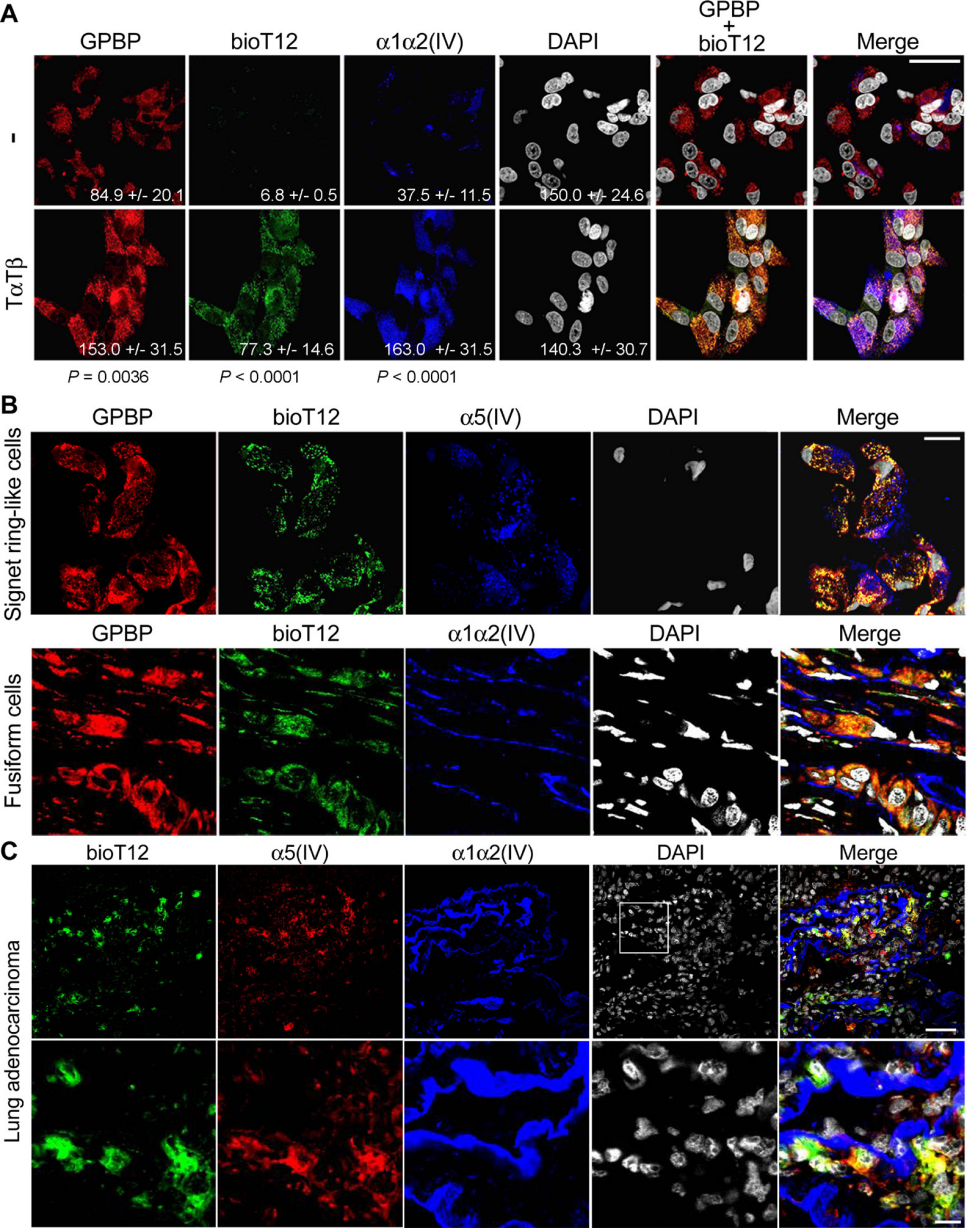
T12 selectively targets mesenchymal GPBP produced by cancer EMT phenotypes (**A**) A549 cultures (−) were stimulated (TαTβ) for 24 h and analyzed by IF-CM to visualize *in situ* T12 binding (bioT12) and the indicated polypeptides (bar = 20 μm). Indicated are FI measured, expressed and compared as in Figure 1. Statistics: Student’s *t*-test. (**B**) Frozen sections of a small A549 tumor were analyzed by IF-CM to visualize *in situ* T12 binding (bioT12) and the indicated polypeptides at tumor areas populated by the indicated cells (bar = 20 μm). (**C**) Frozen sections of patient adenocarcinoma were IF-CM analyzed to visualize *in situ* T12 binding (bioT12) and the indicated polypeptides in a representative tumor region. In the bottom row, the squared area in the top row is displayed at higher magnification (bottom row bar = 10 μm).

The evidence indicated that T12 targeted the oligomerization process that renders multimer formation, the predominant GPBP species produced and secreted by cancer cells following EMT. To confirm this, we generated biotinylated T12 (bioT12) retaining biological activities (Supplementary Figure 6A and 6B) and stained A549 cultures prior to or after EMT induction by TαTβ stimulation (Figure 6A). BioT12 efficiently bound to GPBP aggregates being produced by stimulated A549 cells that also expressed more mesh α1α2(IV) collagen. In contrast, bioT12 did not bind significantly to unstimulated A549 cultures which expressed limited α1α2(IV) collagen. Further, we investigated bioT12 specificity in A549 xenografts (Figure 6B). As expected, bioT12 bound to GPBP aggregates produced by fusiform and signet ring-like cells respectively expressing α1α2(IV) and α5(IV) networks and representing tumor EMT phenotypes. Consistent with this, bioT12 also bound to 4T1, Lewis lung cancer (LLC) xenografts and no significant binding of bioT12 was observed in the tissues of control mice (Supplementary Figure 6C). Finally, in naturally occurring NSCLC bioT12 bound to many tumor cells expressing abundant GPBP (Supplementary Figure 6D), mesh α5(IV) and α1α2(IV) collagens (Figure 6C). Collectively, our findings suggested that T12 targets GPBP species expressed by tumor cells following EMT and referred to here as “mesenchymal GPBP”.

### T12 impairs mesh collagen IV formation and reduces tumor burden in mouse models

**Figure 7:**
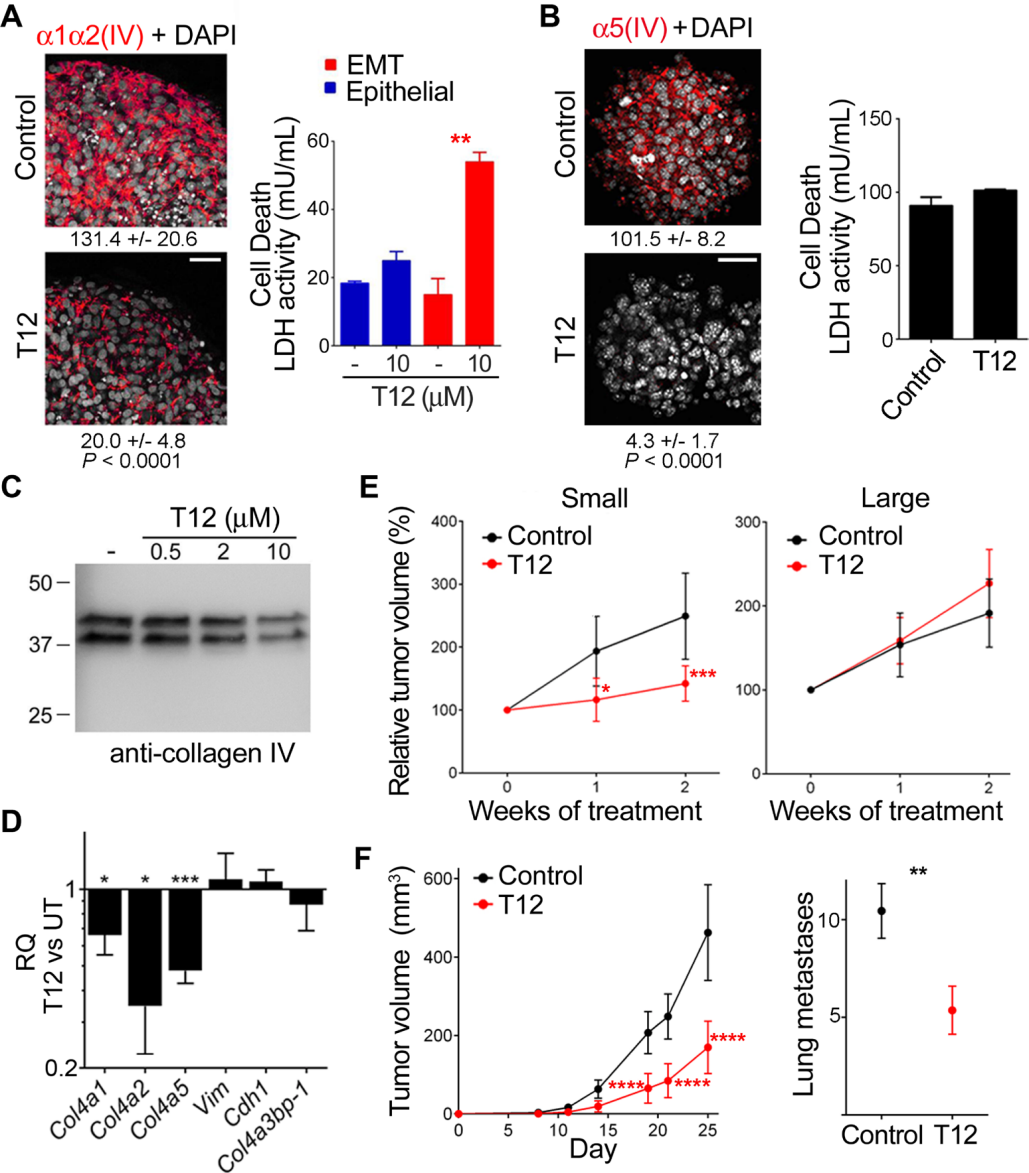
T12 impairs mesh collagen IV formation and reduces tumor burden in mouse models (**A**) TαTβ-stimulated A549 and (**B**) A7C11 spheroids were treated (48 h) and analyzed by IF-CM to visualize the indicated polypeptides (images) and cell death measured as in Figure 2C and represented with bars (histograms). Statistics: Student’s *t*-test, *n* = 3 (A) *n* = 2 (B). Indicated are FI measured, expressed and compared as in Figure 1 Statistics: Student’s *t*-test. (**C**) The extracellular matrix of A7C11 spheroids subjected to the indicated treatments (48 h) was collagenase digested and analyzed by WB using mAb 202. (**D**) The RQ of the indicated mRNA in treated (10 µM, 36 h) A7C11 spheroids (T12) vs untreated (UT) is represented as in previous figures. Statistics: Student’s *t*-test, *n* = 2. (**E**) A549 mouse-model bearing the indicated xenografts was untreated (Control) or treated (T12) and the indicated variable determined over time and plotted (mean ± 95% confidence interval, CI). Tumor volume at treatment initiation was set to 100%. Statistics: 2WA-S, *n* = 15. (**F**) Plotted are indicated variables (mean± 95% CI) in 4T1 mouse-model untreated (Control) or treated (T12) over time (left) or at day-25 (right). Statistics: *Left*, 2WA-S, *n* = 10. Representative of three assays. *Right*, Student’s *t*-test, *n* = 30.

To analyze the consequence of T12 treatment on EMT phenotypes and associated mesh collagen IV networks, we treated EMT A549 spheroids populated by mesenchymal progenitor cells with mesh α1α2(IV) collagen network (see above) and A7C11 spheroids containing more differentiated EMT phenotypes that expressed also mesh α5(IV) collagen network. At 10 µM, T12 inhibited mesh α1α2(IV) collagen formation in EMT A549 spheroids and induced cancer cell death (Figure 7A), at least in part, by mediating spheroid cell detachment (Supplementary Figure 7A). In contrast, T12 treatment of A7C11 spheroids efficiently inhibited mesh α5(IV) collagen formation without inducing cell death (Figure 7B). To further explore the effects of T12 treatment on mesh collagen IV expression, we subjected A7C11 spheroids-extracellular matrix to bacterial collagenase-digestion and WB analysis of released NC1 materials using monoclonal antibody (mAb) 202, which recognizes an epitope conserved in all six α(IV) chains (Figure 7C). WB analysis revealed two major NC1 polypeptides whose relative abundance decreased with T12 treatment. The M_*r*_ of these polypeptides was consistent with dimeric rather than with monomeric NC1 material. This suggested that treatment reduced mesh collagen IV expression, which exhibited head-to-head protomer supramolecular organization (NC1-to-NC1) with prominent covalent crosslinking reinforcement. To investigate individual mesh collagen IV network expression inhibition, we performed mRNA quantification which revealed that T12 reduced *Col4a1, Col4a2* and particularly *Col4a5* expression (Figure 7D). Subsequent toxicology studies in mice revealed T12 to be a safe compound up to 400 mg/kg/day when administered orally for 8 days; T12 displayed efficient absorption and bioavailability (see Toxicokinetics in Auxiliary Supplementary Materials). Thus, we tested the anti-tumor potential by administrating T12 in the drinking water (0.1 mg/mL) to nude mice with A549 tumors (Figure 7E). Small xenografts (200–300 mm^3^) responded to T12 whereas large xenografts (>300 mm^3^) did not. Further, we assessed the effect of T12 on tumor load in immune-competent murine breast cancer 4T1 model (Figure 7F). Early administration of T12 (from day-0) reduced primary tumor growth and metastases formation: 27% of the mice treated with T12 had no superficial lung metastases. In these preclinical studies of efficacy, T12 plasma concentrations at steady state were 2.3 ± 0.53 µM (*n* = 5). Based on pharmacokinetic data, T12 plasma concentrations using oral dosing reached 10 µM or higher in mouse plasma (see Toxicokinetics in Auxiliary Supplementary Materials). Therefore, we further characterized T12 anti-cancer activity at higher concentrations using a variety of different cell culture systems. At 10–50 µM, T12 induced apoptosis of cultured c4T1 cells (Supplementary Figure 7B) and 50–100 µM of T12 were needed to induce apoptosis in A427 cultures (Supplementary Figure 7C). In all these studies, the cultures of epithelial counterparts used as control were far less prone to apoptosis. The IC50 of T12 was determined using a hundred different cancer cell lines and was 58.9 ± 37.6 µM (see IC50 Cancer Cell Line Panel in Auxiliary Supplementary Materials), suggesting that T12 at 50–100 µM also compromised cancer epithelial phenotype through a non-apoptotic mechanism. Consistently, at these concentrations T12 induced accumulation of A549 cells in G0/G1 phase (Supplementary Figure 7D) and thus, inhibited cell proliferation (Supplementary Figure 7E). Higher concentrations (100–200 µM) of T12 were required to compromise viability of telomerase-immortalized cultures representing human (epithelial and mesenchymal) non-malignant cells (Supplementary Figure 7F), without any significant cytotoxicity (Supplementary Figure 7G). However, at these higher concentrations, T12 was cytotoxic for cancer epithelial A549 cultures (Supplementary Figure 7G) and still more for the A549-DR cultures (Supplementary Figure 7H). Finally, T12 exhibited no mutagenicity and limited cross-reactivity with other protein kinases at the therapeutic steady state mouse plasma concentration. Moreover, T12 was metabolized rapidly by cultured mouse hepatocytes exhibiting a half-life of 4.36 min (see corresponding Reports in Auxiliary Supplementary Materials). Collectively, these data suggested that T12 was a safe and reliable drug candidate.

### T12 impedes tumor epithelial-mesenchymal transdifferentiation

**Figure 8:**
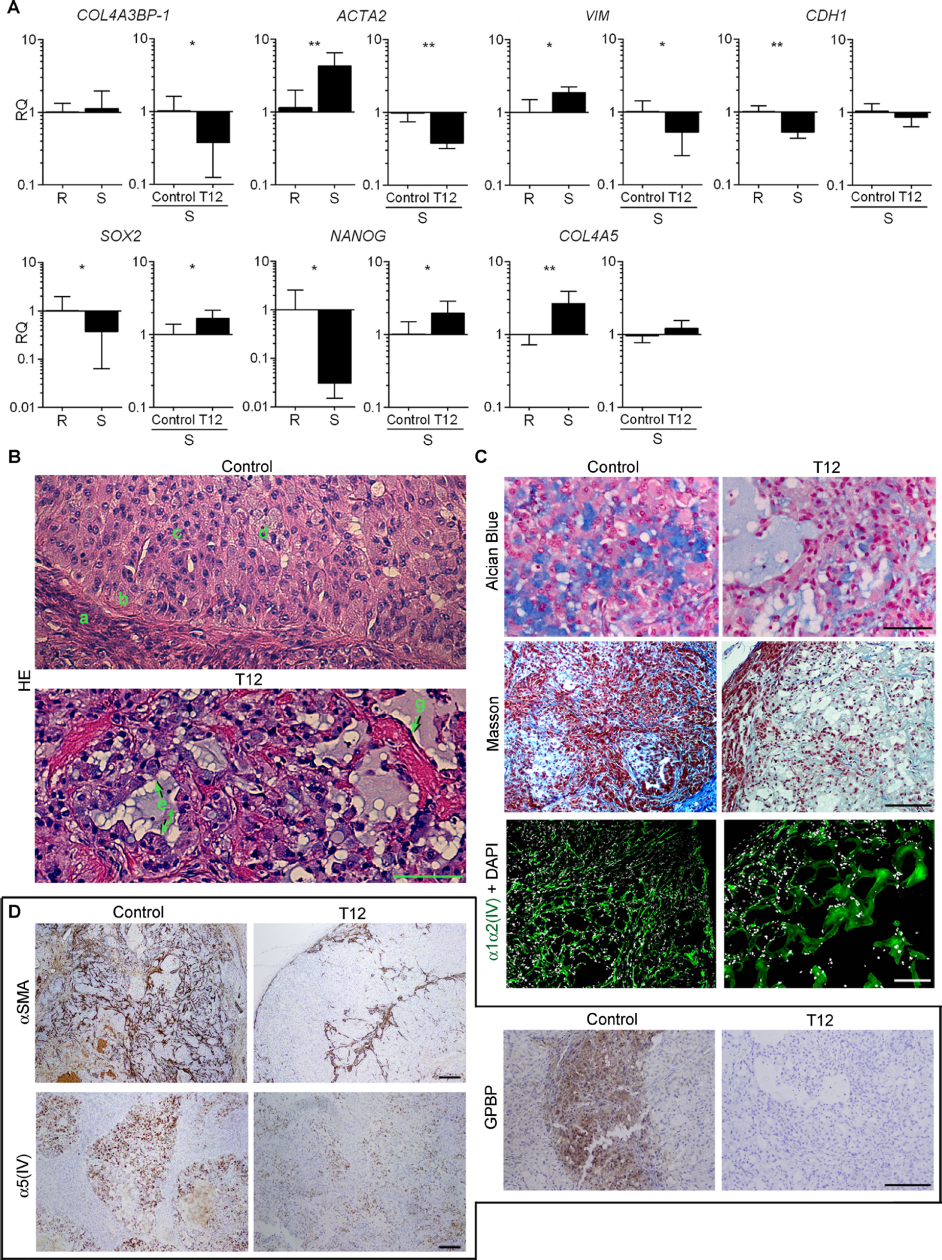
T12 impedes tumor epithelial-mesenchymal transdifferentiation (**A**) Bars represent RQ (mean ± SD) of the indicated mRNA in small sensitive (S, *n* = 14) vs large refractory (R, *n* = 8) A549 tumors and in T12-treated (*n* = 6) vs untreated (control, *n* = 6) S-A549 tumors. Statistics: Student´s *t*-test. (**B**–**D**) Paraffin-embedded sections from untreated (Control, *n* = 6) or treated (T12, *n* = 6) S-A549 tumors were stained with the indicated reagents or subjected to IF-CM or standard immunohistochemical procedures to visualize the indicated polypeptides. Immunohistochemical specimens were counterstained with hematoxylin. Shown are selected representative images. Bar = 100 μm in B and C (Alcian Blue); Bar = 200 μm in C (Masson, IF-CM) and D.

We used RT-qPCR to analyze the expression of relevant EMT biomarkers in small sensitive (S) vs large refractory (R) A459 tumors to T12 treatment (Figure 8A). S-tumors expressed more of the mesenchymal biomarkers (*VIM*, *ACTA2*, *COL4A5*), and less of those representing epithelial (*CDH1*) and progenitor (*SOX2, NANOG*) phenotypes than R-tumors. No significant differences were found in the relative expression of *COL4A3BP-1* between S- and R-tumors. Interestingly, T12 treatment reduced *ACTA2* and *VIM* in S-tumors but increased the relative expression of *SOX2* and *NANOG*. Treatment also reduced *COL4A3BP-1* but did not significantly change *COL4A5* expression. We investigated treatment effects using histological analysis of HE specimens (Figure 8B). In untreated S-tumors, fusiform cells were found in the stroma (a) encircling nodular regions with epithelial cells at the periphery (b) that detached (c) to generate signet ring-like cells (d). Treatment stimulated signet ring-like cells to secrete the accumulated mucus (e) and to re-epithelize (f), and reduced fusiform cell population which now appeared encircling an enlarged stromal matrix (g). Alcian blue staining confirmed the presence of abundant extracellular mucus in nodular regions of treated S-tumors and combined Masson and IF-CM analysis revealed that the enlarged stromal matrix consisted of an expanded mesh α1α2(IV) network (Figure 8C). Treatment sharply reduced the expression of α-SMA in stroma, α5(IV) in nodules and GPBP in both stroma and nodules of the tumor (Figure 8D). Altogether, these observations suggested that T12 treatment disrupted mesh α5(IV), thereby stabilizing signet ring-like cells and inducing re-polarization and intracellular mucus release. Moreover, T12 reduced fusiform cell population and increased the relative proportion of the corresponding progenitor cells (*SOX2*, *NANOG*) which expressed an abundant mesh α1α2(IV) network. Finally, we found no evidence for cell death associated with treatment. Intriguingly, treated R-tumors exhibited reduced expression of *CDH1* and *SOX2* without evident consequences on epithelial cell organization (Supplementary Figure 8) or tumor growth (see above), suggesting that GPBP oligomers sensitive to T12 also maintain to some extent the epithelial progenitor phenotype. Collectively, the findings suggested that T12 treatment impaired A549 tumor growth by inhibiting EMT.

### Low-dose doxorubicin sensitizes epithelial cancer progenitor cells to T12

**Figure 9:**
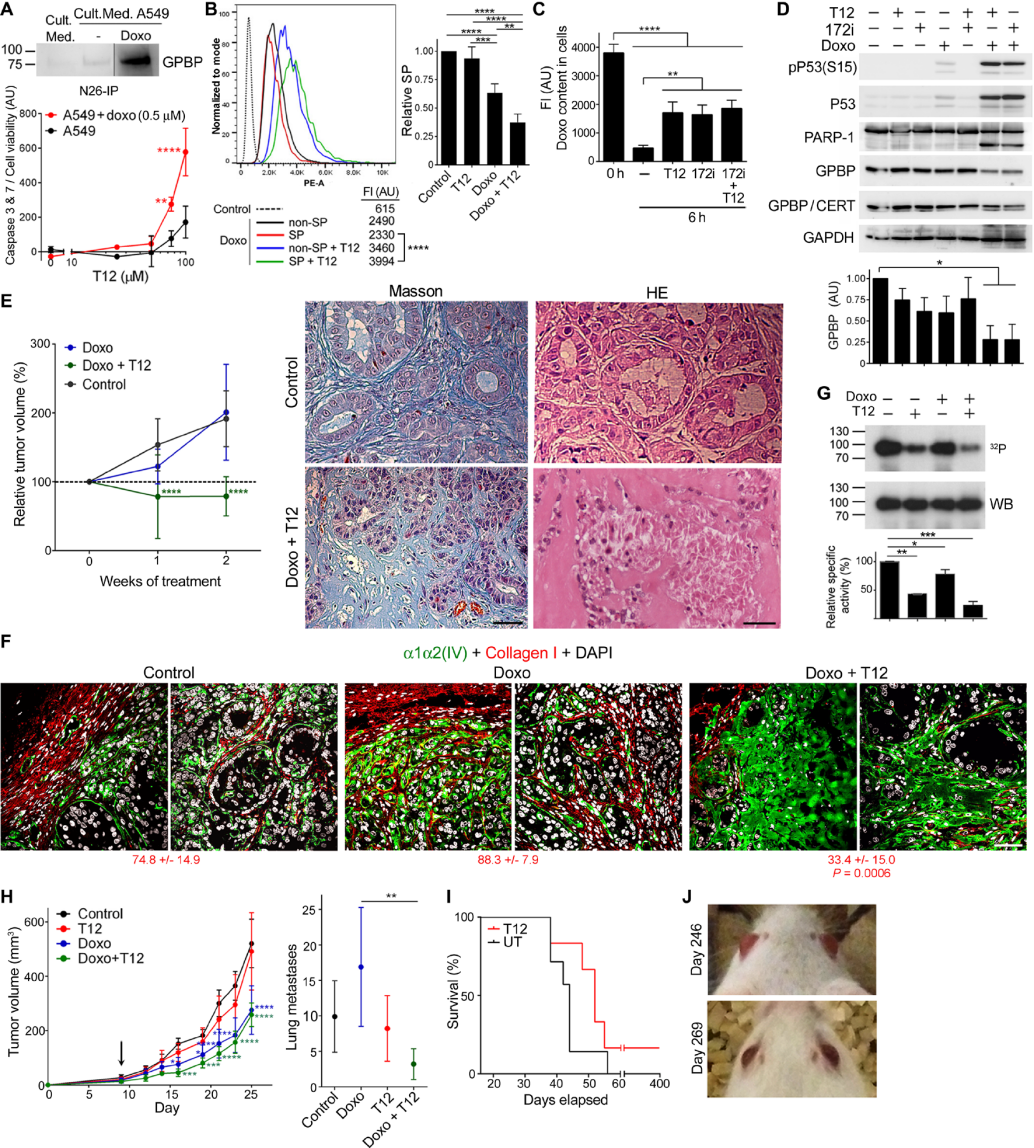
Low-dose doxorubicin sensitizes epithelial cancer progenitor cells to T12 (**A**) WB analysis with N27 of the indicated immunoprecipitates (IP). A549 cells were treated as indicated (42 h) and normalized caspase activity plotted (mean ± SD). Statistics: 2WA-S, *n* = 3. Representative of three and two assays, respectively. (**B**) *Left,* A549 cultures were treated as indicated (40 h) and analyzed by flow cytometry following doxorubicin fluorescence in SP and non-SP. Ordinate: Event numbers normalized to mode. Abscise: Phycoerythrin channel autofluorescence intensity (PE-A). Shown are mean FI. Statistics: Student’s *t*-test, *n* > 1,000. Representative of four assays. *Right*, relative SP content (mean ± SD) in treated respect untreated (Control, SP = 1) cultures. Statistics: 1WA-T, *n* = 4. (**C**) A549 cultures were doxorubicin-treated (0 h) and further cultured in doxorubicin-free medium with the indicated inhibitors (6 h) and analyzed as in B. Doxorubicin content (mean ± SD) was determined subtracting cell autofluorescence. Statistics: 1WA-T, *n* = 3. (**D**) A549 cultures were treated (40 h) with the indicated reagents and WB analyzed. Plotted is GAPDH-normalized GPBP expression (mean ± SD). Statistics: 1WA-D, *n* = 2. (**E**) Nude mice bearing large A549 tumors were treated and the indicated variable (mean ± 95% CI) plotted over time. Initial tumor size: 100%. Statistics: 2WA-S, *n* = 14. Groups in this and Figure 7E (Large) were from the same assay. Images were selected from the indicated analysis and tumors. (**F**) Representative images of tumors in E analyzed by IF-CM. Indicated are FI measured and expressed as in Figure 1. Statistics: 1WA-D comparison respect to Control. (**G**) FLAG-GPBP autophosphorylation mixtures were analyzed and represented as in Figure 5A. Statistics: 1WA-F, *n* = 2. (**H**) 4T1 mouse-model was treated from day-9 (arrow) and the indicated variable (mean ± 95% CI) plotted over time (left) or at day-25 (right). Statistics: *Left*, 2WA-S, *n* = 10. *Right*, 1WA-T, *n* = 10. (**I**) Kaplan–Meier survival curves of 4T1 mouse-model T12-treated after surgical removal of primary tumor at day-16. Statistics: Log-rank (Mantel-Cox) test, *P* = 0.15. (**J**) Pictures of mouse-1 at the indicated days post-inoculation. Unless otherwise indicated, culture treatments were: doxorubicin, 1 µM; T12, 50 µM; and 172i, 25 µM.

Doxorubicin has been reported to induce EMT [23], suggesting that it might be used to sensitize large A549 tumors to T12. Accordingly, following doxorubicin treatment, A549 cultures efficiently bound bioT12 (Supplementary Figure 9A), secreted GPBP, and responded to T12 by undergoing apoptosis (Figure 9A). Interestingly, T12 induced intracellular doxorubicin accumulation and reduced the side population (SP) of A549 cultures (Figure 9B) which contains progenitors expressing ABC transporters for xenobiotic extrusion [24]. Differential mRNA expression analysis revealed that T12 induced *DDIT3* that encodes CHOP, a transcription factor inducing apoptosis in response to ER protein misfolding (unfolded protein response or UPR) [25], and potentiated doxorubicin-induced *DDIT3* and *CFTR* expression (Supplementary Figure 9B). *CFTR* encodes for a unique ABC member which acts as monovalent anion channel instead of xenobiotic extruder [26]. All the above observations suggested that doxorubicin induced UPR in epithelial cancer progenitor cells and promoted a novel adaptive response based on CFTR recruitment for doxorubicin extrusion. T12 or the CFTR inhibitor 172 (172i) reduced doxorubicin extrusion (Figure 9C). Consistent with this, these inhibitors enhanced doxorubicin-induced p53 activation and intriguingly, when combined with doxorubicin, induced PARP-1 processing (89-kDa fragment) and reduced GPBP levels (Figure 9D). These effects were suggestive of necroptosis, a doxorubicin-induced PARP-1-dependent regulated necrosis which requires *COL4A3BP* activation [27, 28]. In support of this hypothesis, T12 combined with low-dose doxorubicin arrested large A549 tumor growth (Figure 9E, graph) and caused necrosis of “mesenchymalized” epithelial cells (Figure 9E, images). Specifically, an enlarged desmoplastic stroma (Masson) contained abundant “mesenchymalized” epithelial tumor cells that underwent enucleation and necrosis (shadow-like cells). RT-qPCR analysis of tumor revealed that doxorubicin induced *ACTA2*, *VIM* and *SOX2* whereas addition of T12 reduced *SOX2* expression (Supplementary Figure 9C). Altogether, these data suggest that T12 combined with low-dose doxorubicin selectively eliminated progenitor cells resulting, at least in part, from “mesenchymalization” of tumor epithelial structures.

Tumor IF-CM analysis supported these conclusions and unveiled new insights (Figure 9F). Specifically, low-dose doxorubicin disrupted basement membrane collagen IV and tumor epithelial organization, revealing conversion of epithelial tumor cells to a mesenchymal state. Low-dose doxorubicin alone induced an intracellular accumulation of collagen IV (see Supplementary Figure 9D for more magnification) but caused no significant reduction of the tumor cell number. However, addition of T12 induced desmoplastic (expanded and irregular) collagen IV formation and a sharp reduction in the tumor cell number. Moreover, large A549 tumors exhibited abundant collagen I (fibrosis) of murine origin specifically recognized by our antibodies that encapsulated tumor and encircled epithelial structures inside the tumor. Interestingly, doxorubicin enhanced and T12 prevented doxorubicin-induced tumor fibrosis, which was consistent with the previous observation that GPBP antibodies inhibited lung fibrosis in doxorubicin-induced mouse-model.^3^

Moreover, it was shown the existence of GPBP species sensitive to doxorubicin (Figure 9G) that underwent secretion following A549 doxorubicin treatment (Supplementary Figure 9E). Altogether, the data revealed that efficacy of combined T12-doxorubicin treatment of epithelial progenitors depended on secretion of doxorubicin-responsive GPBP species referred to here as “epithelial GPBP”, along with mesenchymal GPBP species.

We further explored doxorubicin sensitization of 4T1 mouse-model by initiating T12 treatment at day-9 when tumors were larger and suspected to be more of the epithelial phenotype. Doxorubicin alone was sufficient to reduce primary tumor growth but prior exposure to doxorubicin was required for T12 to inhibit metastases formation (Figure 9H) and to reduce *Sox2* expression (Supplementary Figure 9F). In contrast, doxorubicin did not enhance T12 efficacy treating small A549 tumors (Supplementary Figure 9G), confirming that cancer EMT phenotypes do not express the form of GPBP sensitive to doxorubicin.

We further investigated T12 effect on metastases progression by performing a survival curve in 4T1 mouse-model after primary tumor removal at day-16. T12 treatment extended mice lifespan, although all mice eventually died within two months except one (Figure 9I). This animal (mouse-1) was maintained with T12 treatment for several months and subjected to positron emission tomography–computed tomography (PET-CT) analysis, which revealed at least two metastatic nodules that allowed *in vivo* monitoring of treatment on progression of secondary tumors. Cessation of treatment triggered increased activity in cancer nodules as detected by PET-CT and therefore, treatment was reinstated. However, the follow-up PET-CT analysis revealed persistent cancer nodules (Supplementary Figure 9H) and the presence of a fast-growing brain metastasis that caused exophthalmos (Figure 9J). Then, we combined T12 with low-dose doxorubicin which resulted in decrease of cancer nodules and almost complete resolution of exophthalmos. After a few months of combined treatment, we removed doxorubicin and treatment with T12 was continued. A similar survival experiment curve resulted in a second surviving mouse (mouse-2) which was subjected to similar procedures with equivalent results (Supplementary Figure 9I). Supplementary Table 2 presents detailed pre-clinical information obtained from monitoring mouse-1 and -2. Thus, data suggested that low-dose doxorubicin also induced EMT in metastatic tumors and sensitized them to T12.

### Extracellular GPBP instigates pathogenesis

**Figure 10:**
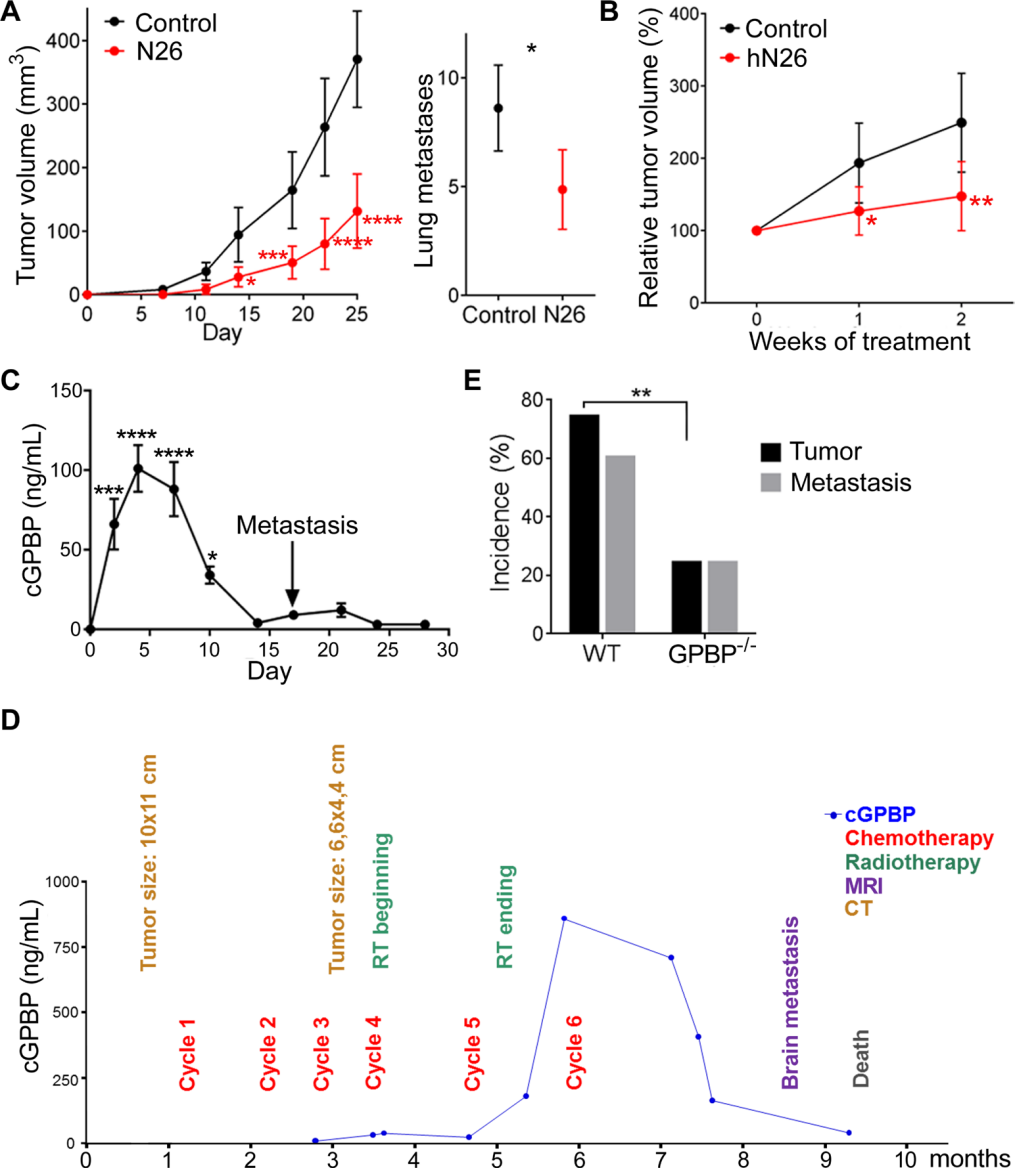
Extracellular GPBP instigates pathogenesis (**A**) Represented are the indicated variables in 4T1 mouse-model untreated (Control) or treated (N26) over time (left) or at day-25 (right). Statistics: *Left*, 2WA-S, *n* = 10. Representative of three assays. *Right*, Student’s *t*-test, *n* = 30. (**B**) Tumor growth over time in A549 mouse-model untreated (Control) or treated (hN26). Initial tumor volume (200–300 mm^3^) was set as 100%. Statistics: 2WA-S, *n* = 14. (A, B) Values are mean ± 95% CI. (**C**) cGPBP levels (mean ± SD) were monitored over time in 4T1 mouse-model using the preclinical EMTEST. Macroscopic metastases appear on day-17 (arrow). Statistics: 1WA-D. Two mice were sacrificed per day. Representative of three assays. (**D**) Time course flowchart of cGPBP levels and relevant clinical events for Patient 1. cGPBP measurements were done on fresh serum samples using preclinical EMTEST and samples stored at –80^°^ C. MRI, magnetic resonance imaging; CT, computerized tomography. (**E**) Percentage of mice with primary tumor and percentage of mice with primary tumor also developing lung metastases at day-28 in LLC mouse-model. Statistics: Fisher’s exact test. Tumor: *n* = 17; Metastases: WT *n* = 14, GPBP^−/−^ *n* = 4.

The above evidence suggested that antitumor effects of T12 depended, by and large, on targeting multimeric mesenchymal GPBP secreted by the tumor. To further confirm this, the GPBP-blocking mAb N26 (Supplementary Figure 10A) was used to treat 4T1 mouse-model (Figure 10A). Early administration of N26 reduced tumor growth and metastases formation: 20% of the mice were without lung metastases. To further explore extracellular GPBP-mediated pathogenicity and potential therapeutic exploitation of N26, we generated hN26, a chimeric version of N26 (human constant regions and murine variable regions), that was tested in the humanized A549 mouse-model (Figure 10B). Chimeric hN26 antibody reduced A549 tumor growth with similar time course as T12. Collectively, the data suggested that extracellular GPBP was strongly associated with malignancy. Thus, we developed the EMTEST assay for selective monitoring of circulating GPBP (cGPBP). EMTEST, a mAb-based sandwich enzyme-linked immunosorbent assay (ELISA), suitable for GPBP measurement in mouse plasma (Supplementary Figure 10B), confirmed that GPBP was secreted from A549 cells upon EMT induction (Supplementary Figure 10C). Monitoring of 4T1 mouse-model by EMTEST revealed progressive elevation of cGPBP levels after cell inoculation (day-0), reaching peak levels around day-5 followed by a progressive decline up to day-12 before detection of lung metastases at day-17 (Figure 10C). Inoculation of control, non-tumor forming cells in this mouse model failed to trigger elevated cGPBP levels (Supplementary Figure 10D). Hence, our findings suggest that the tumor formation in this pre-clinical model strongly correlated with increased cGPBP levels and subsequent clearance of cGPBP preceded metastasis. *COL4A3BP* gene expression levels have been associated with poor outcomes in breast [29] and lung cancer patients (Supplementary Figure 10E), suggesting that cGPBP levels might play a role in metastasis. To extend the relevance of our findings to the clinical setting, we monitored cGPBP levels in a NSCLC patient (stage IIIA) subjected to combined cisplatin-based chemotherapy and radiotherapy (Figure 10D). Chemotherapy reduced by approximately 50% the initial tumor volume before radiotherapy was initiated. Following radiotherapy, cGPBP levels sharply increased, suggesting that cGPBP levels were induced by tumor EMT phenotypes that were resistant to both radio and chemotherapy. Elevated cGPBP levels were maintained for one month and sharply cleared before brain metastasis and severe cachexia caused patient’s death (Patient 1). Subsequently, we adapted EMTEST for human samples using antibody constructs devoid of Fc region (scFvN26 and FabN27) to avoid potential interference with rheumatoid-like factors recognizing the murine Fc region of our mAb (Supplementary Figure 10F) and monitored cGPBP levels in plasma samples in two additional NSCLC patients (Supplementary Figure 10G). cGPBP levels were monitored in a patient at stage IIA and disease-free status following surgery and adjuvant cisplatin-based chemotherapy (Patient 2). In this patient, cGPBP levels were already detectable during adjuvant chemotherapy and sharply increased a few months after and remained elevated for more than a year when they sharply decreased prior to tumor relapse when multiorgan metastases caused patient’s death. Finally, in a patient with idiopathic lung fibrosis and type 2 diabetes (Patient 3), cGPBP levels were low prior to diagnosis of infiltrating mediastinum lymphoid nodes in the absence of detectable primary tumor (T0N1M0). Interestingly, combined chemotherapy and radiotherapy in this patient resulted in disease-free status, correlating with undetectable levels of cGPBP. These observations suggest that cancer cells infiltrating lymph nodes were the source of increased cGPBP levels and that ongoing remission in this patient is associated with undetectable levels of cGPBP.

As a parallel approach to confirm the relevance of extracellular GPBP for tumor initiation and progression, LLC cells were introduced into GPBP^−/−^ mice (Figure 10E). Fewer GPBP^−/−^ mice developed tumors (24% in GPBP^−/−^, 76% in WT) or lung metastases (25% in GPBP^−/−^, 62% in WT), demonstrating that deficiency of host GPBP also reduced tumor load. Collectively, the data revealed that extracellular GPBP deriving from both tumor and host instigates pathogenesis.

### Epithelial cells clear tumor mesenchymal cGPBP levels and generate a previously unrecognized form of GPBP

**Figure 11:**
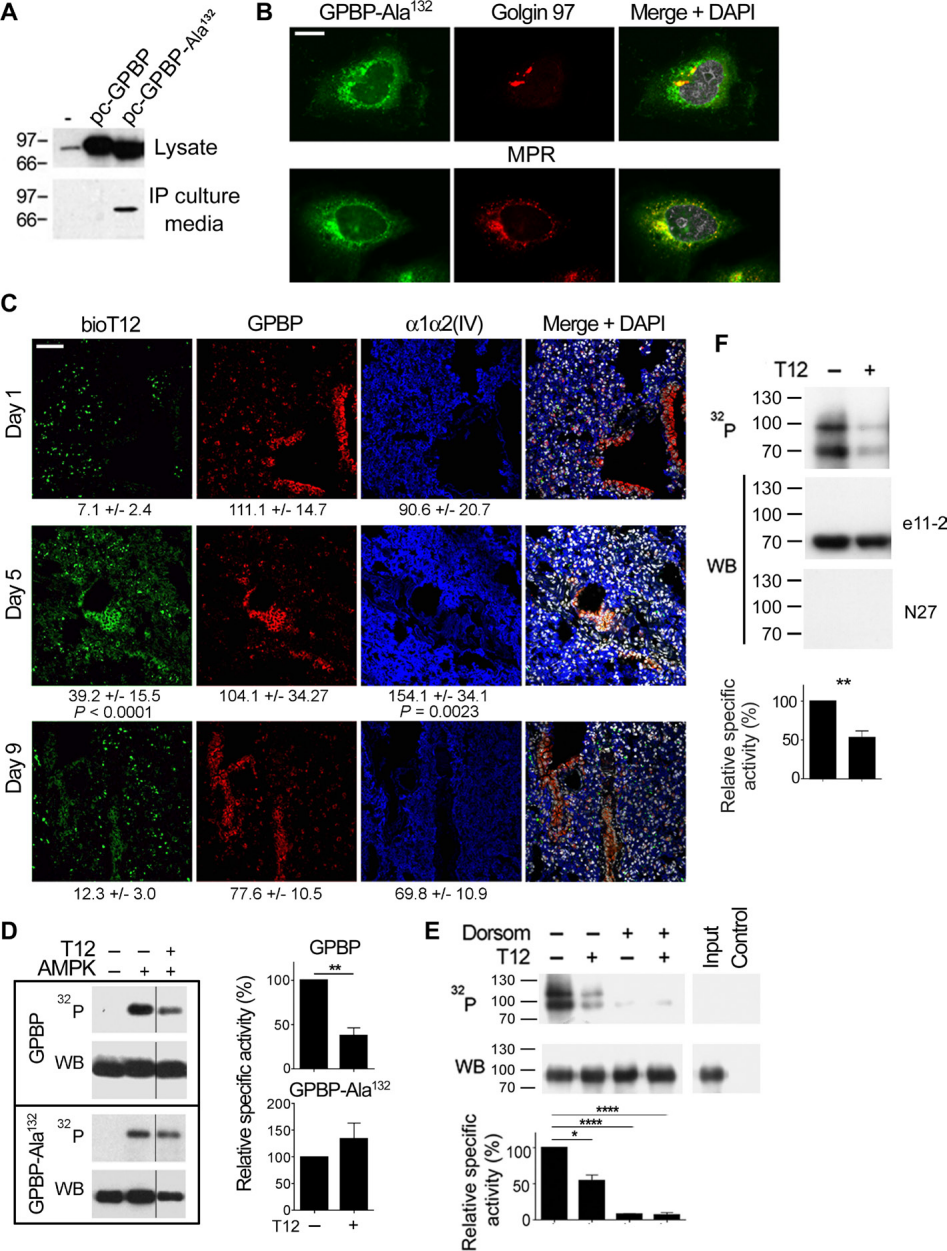
Epithelial cells clear tumor mesenchymal cGPBP levels and generate a previously unrecognized form of GPBP (**A**) HEK 293 cells were transfected with pcDNA3 (–) or the indicated derived constructs coding for FLAG-GPBP or FLAG-GPBP-Ala^132^. After 36 h, equivalent amounts of the indicated materials were analyzed by WB with anti-GPBP mAb 14 [3]. (**B**) FLAG-GPBP-Ala^132^ transfected HEK 293 cells were analyzed by IF-CM using specific antibodies for the indicated polypeptides. FLAG-GPBP-Ala^132^ was visualized with anti-FLAG. MPR, mannose-6-phosphate receptor (bar = 10 μm). (**C**) Frozen lung sections from 4T1 mouse-model sacrificed at the indicated days (*n* = 2) were analyzed by IF-CM to visualize *in situ* T12 binding (bioT12) and the indicated polypeptides. FI was measured and expressed as in Figure 1. Statistics: 1WA-D comparison respect to day 1. (**D**) The phosphorylation mixtures containing the indicated FLAG-polypeptides and reagents were analyzed and represented as in previous figures. WB was developed with α-FLAG antibody. (**E**) A549 cultures were supplemented with BM40-FLAG-GPBP for 6 h in the presence (+) or absence (–) of dorsomorphin. BM40-FLAG-GPBP purified from cellular extracts was phosphorylated with (+) or without (–) T12 and similarly analyzed and represented. Phosphorylation mixtures of BM40-FLAG-GPBP (Input) and anti-FLAG immunoprecipitated material from mock A549 cultures (Control) are also displayed. WB, was developed with N27 antibody. Where indicated in D and E, dorsomorphin and T12 were used at 40 and 50 µM, respectively. Statistics: Student´s *t*-test, *n* = 4 (D) *n* = 2 (E). (**F**) cGPBP from NSCLC patient’s plasma (0.5 mL) was immunopurified with scFvN26, released using appropriated blocking peptide and subjected to *in vitro* phosphorylation assay. Resulting phosphorylation mixtures were analyzed and represented as in Figure 5B. Statistics: Student’s *t*-test, *n* = 3. Similar results were obtained when pull-down assays were done using scFvN27 and the corresponding blocking peptide, suggesting that cGPBP multimeric structures contained a minority of N27-reactive material that allowed immunoprecipitation and limited detection by EMTEST (<1 ng/mL).

It has been reported that the binding of the two-phenylalanine(FF)-in-an-acidic-tract (FFTA) motif binding to (vesicle-associated membrane protein)-associated protein-A (VAPA) mediates GPBP exportation [4] and that phosphorylation of Ser^132^ inhibits GPBP-2/CERT binding to VAPA [30]. These observations prompted us to postulate that GPBP phosphorylation at Ser^132^ impairs exportation. Consistent with this, FLAG-GPBP-Ala^132^ mutant was more efficiently exported than FLAG-GPBP in a HEK 293 cell system for recombinant protein expression and secretion (Figure 11A). Intracellular FLAG-GPBP-Ala^132^ displayed a slightly lower M_*r*_ than wild-type GPBP, supporting the notion that recombinant intracellular GPBP was phosphorylated on Ser^132^ and this inhibited its exportation. IF-CM analysis revealed that GPBP-Ala^132^ distributes to Golgi apparatus (Golgin 97), trans-Golgi network and endosomes (mannose 6-phosphate receptor, MPR) (Figure 11B). This contrasts with the previously reported distribution of GPBP to the ER [4] and suggested that FLAG-GPBP-Ala^132^ underwent exportation and reuptake. Subsequently, we investigated *in vivo* the uptake of tumor mesenchymal cGPBP by the epithelial cells of the lung in 4T1 mouse model. For this purpose, lung specimens were stained with bioT12 (Figure 11C), which specifically recognizes mesenchymal GPBP produced and secreted by the tumor EMT phenotypes (see above). At day-1, when cGPBP levels were very low, bioT12 failed to stain lung structures; however, bioT12 stained epithelial cells of the bronchial mucosa and the parenchyma at day-5 when cGPBP levels were high. In parallel with the drop in cGPBP levels, staining of bioT12 was reduced at day-9. These findings revealed that normal epithelial cells clear, at least in part, mesenchymal cGPBP levels secreted by the tumor. Insulin-dependent Akt kinase has been reported to be enhanced in GPBP^−/−^ myocytes [21], suggesting that intracellular cytosolic GPBP reduced sensitivity to insulin. In this regard, phosphorylation of Ser^132^ has been described to downregulate intracellular ceramide transport [31] which would be expected to increase intracellular ceramide levels and to reduce sensitivity to insulin [32]. Ser^132^ comprises a phosphorylation site (Scansite Motif Scanner from Massachusetts Institute of Technology) for AMP-activated protein kinase (AMPK), suggesting that captured extracellular GPBP could undergo AMPK phosphorylation and reduce insulin-dependent glucose uptake. To investigate phosphorylation of GPBP on Ser^132^ by AMPK, we engineered and produced BM40-FLAG-GPBP and BM40-FLAG-GPBP-Ala^132^. The introduction of the basement membrane protein 40 (BM-40) particle recognition signal was designed to limit GPBP synthesis to the ER and prevent intracellular AMPK targeting. Subsequently, intracellular FLAG-purified material was used for *in vitro* phosphorylation assays in the presence or absence of T12 (Figure 11D). AMPK activated both forms, but only BM40-FLAG-GPBP responded to T12, suggesting that AMPK phosphorylation of Ser^132^ activated intracellular GPBP multimerization. To assess GPBP activation by epithelial cells, A549 cultures were supplemented with BM40-FLAG-GPBP and either treated or not with dorsomorphin, a competitive inhibitor of AMPK suitable for *ex vivo* studies. Then, T12 responsiveness of FLAG-purified intracellular materials was assessed by *in vitro* autophosphorylation (Figure 11E). BM40-FLAG-GPBP extracted from untreated cultures exhibited enhanced activity and responsiveness to T12, which contrasted with a limited autophosphorylation non-sensitive to T12 displayed by BM40-FLAG-GPBP extracted from dorsomorphin-treated cultures. Epithelial-activated BM40-FLAG-GPBP phosphorylation mixtures contained a novel T12-responsive species of higher M_*r*_ that was not reactive with N27 (Figure 11E) nor with α-FLAG (not shown) antibodies, suggesting that it may represent a low abundance phosphorylation-dependent gel-shifted BM40-FLAG-GPBP material. Our data suggested that epithelial cells captured and activated cGPBP but the clearance of this activated material from the lung (see above) suggested further secretion. This was investigated *in vivo* by isolating cGPBP from the plasma of NSCLC patients and subjecting it to *in vitro* autophosphorylation (Figure 11F). We identified two major phosphorylated GPBP species responsive to T12, one displaying conventional M_*r*_ and another of higher M_*r*_, Intriguingly, neither of these native cGPBP species reacted with N27, the antibody used for detection in EMTEST. The presence of GPBP in the mixture was confirmed using mAb e11–2, which reacted with the GPBP species exhibiting conventional M_*r*_ and failed to detect the higher M_*r*_ species, further confirming represented low abundance phosphorylation-dependent gel-shifted activated GPBP species. The data also suggested that N27 epitope masking specifically associated with re-secretion from epithelial cells and not with secretion from EMT phenotypes (see A427 in Figure 5B above). Thus, revealing that generation of activated cGPBP species required *in vivo* conditions and the contribution of epithelial cells. Consistent with this notion, a patient with Goodpasture disease, a GPBP-EMT-based disorder [5], displayed a profile of circulating GPBP species similar to that of the NSCLC patients. The profile of a healthy control individual was distinct (Supplementary Figure 11). Collectively, the evidence suggested that detection of mesenchymal cGPBP by EMTEST correlated with an early stage of pathology and that decreased levels depended on epithelial phenotype clearance and re-circulation of non-detectable epithelial-activated cGPBP species.

## DISCUSSION

Here we report the novel observation that chemoresistant cancer cells (EMT phenotypes) are maintained by mesh collagen IV networks whose formation is directed by exportable GPBP. Our data indicate that EMT cancer phenotypes secrete GPBP which sensitizes them to the new drug candidate T12 (mesenchymal GPBP). In contrast, epithelial tumor cells express an oligomeric isoform of GPBP which is retained preferentially in the ER and is sensitive to doxorubicin (epithelial GPBP). Immunological blockade of GPBP has anti-tumor effects like those of T12, suggesting that T12 targets extracellular mesenchymal GPBP to avoid malignancy.

In A549 tumors undergoing EMT, epithelial progenitor cells switch to a mesenchymal progenitor phenotype that differentiate into fusiform cells, and epithelial secretory cells switch to signet ring-like cells. Our study suggests that EMT phenotypes express different mesh collagen IV networks for phenotype maintenance: α1α2(IV) for progenitor and fusiform cells and α5(IV) for signet ring-like cells. The anti-tumor effects of T12 depend on perturbation of the mesh collagen IV networks that frame the EMT phenotypes and to some extent epithelial progenitor cell microenvironments. Our studies indicate that doxorubicin inhibits epithelial GPBP and induces UPR in the cancer epithelial phenotype including epithelial progenitor cells. Doxorubicin has been associated with ER protein misfolding [33]. Thus, our evidence suggests that to restore folding conditions, epithelial cancer cells increase GPBP expression and secretion, promoting a mesenchymal state and expansion of progenitor cell population. By inhibiting epithelial-mesenchymal transdifferentiation, T12 cooperates with doxorubicin to expand epithelial progenitor cells that undergoes necroptosis because accumulates doxorubicin (Supplementary Figure 12).

The mechanism by which T12 and CFTR inhibitor 172 (172i) impair xenobiotic (i.e. doxorubicin) extrusion from cells must differ since T12 inhibits (Figure 8) and 172i induces EMT [34]. CFTR regulates transmembrane trafficking of monovalent ions in basement membrane-polarized epithelial cells [26]. Monovalent ions control mucus hydration at the apical side [26] and membrane collagen IV assembly and reinforcement at the basal side [35, 36]. GPBP secretion is associated with membrane collagen IV disruption, mesh collagen IV expansion [12] and CFTR recruitment for xenobiotic extrusion (Figure 9). By inhibiting mesh collagen IV formation, T12 eliminates the protective barrier of epithelial progenitor cancer cells and impedes aberrant CFTR recruitment for xenobiotic extrusion (Supplementary Figure 12). By inhibiting CFTR, 172i impairs apical mucus hydration [26] and induces mesh collagen IV formation [37], although associated recruitment of CFTR for xenobiotic extrusion is inoperative because CFTR is inhibited. The mechanism for mesenchymal GPBP multimerization and its perturbation by T12 remains unknown; however, the observation that CFTR counteracts EMT emphasizes the importance of monovalent ions in these processes. The nature and oligomeric condition of epithelial GPBP remains to be determined.

Intriguingly, antibodies against α5(IV) or GPBP stain A549 xenografts focally at areas populated by tumor cells with EMT phenotypes, suggesting that detection depends in part on mesenchymal supramolecular organizations (mesh α5 network and multimeric GPBP) to enhance antibody binding. At 1–10 μM plasma concentration, T12 inhibits these supramolecular organizations and reduces tumor load. At 50–100 μM, T12 is cytotoxic for cancer EMT phenotypes and inhibits proliferation of cancer epithelial phenotypes by unknown mechanisms. However, at these concentrations T12 does not exhibit deleterious effects on human telomerase-immortalized non-cancer cell lines, implying that increased T12 dosage may further reduce tumors without corresponding toxicity.

Early treatment with T12 inhibits 4T1 metastases formation but not treatment at later timepoints, suggesting that 4T1 metastases sequentially depends on EMT and epithelial phenotypes. Consistently, at later timepoints EMT induction using doxorubicin [23] was associated with metastases formation and responsiveness to T12. In some mice (10%), metastases at later timepoints depended on EMT rather than epithelial phenotypes and T12 impeded metastases progression. In these cases, however, suspension of T12 administration resulted in metastases regrowth. Subsequent treatment with T12 was unable to prevent metastases progression unless combined with low doses of doxorubicin, suggesting that initial EMT-based metastases switched to epithelial-based metastases when T12 treatment was interrupted.

Our studies also suggest that tumor EMT phenotypes spread malignancy by promoting uptake of mesenchymal GPBP into epithelial phenotype. The evidence suggests that captured GPBP is phosphorylated by AMPK at Ser^132^, which is predicted to impair ceramide transportation for sphingomyelin synthesis [30] and to raise ceramide levels which, in turn, inhibit Akt and glucose uptake [32]. Thus, accumulation of mesenchymal GPBP could induce peripheral insulin resistance and drive glucose towards tumor metabolism. The recirculation of epithelial-activated GPBP species and its eventual recapturing by the peripheral tissues may amplify these deleterious effects. The structural basis for the observation that the N27 epitope is masked in secreted epithelial-activated GPBP remains unknown. Phosphorylation of Ser^315^, which is within the epitope cluster recognized by N27, ^4^ has been shown to promote VAPA binding [38]. Conceivably, Ser^315^ phosphorylation might promote secretion of the epithelial-activated GPBP species which is not detectable by EMTEST. Collectively, the evidence suggests that EMTEST is a valuable tool for monitoring chemoresistance or relapse (increase of cGPBP levels) as well as tumor metastasis (decrease of cGPBP levels). Additionally, EMTEST may be useful to monitor the elimination of EMT phenotypes in tumors (i.e. radiotherapy).

TNF-α regulates coordinated expression of C*OL4A3BP* and *POLK*, coding for mutagenic DNA polymerase [39], and related bidirectional transcriptional units for paired *COL4* genes.^5^ Moreover, *COL4A3BP* activation is a signature requirement for regulated necrosis, a process that TNF-α and doxorubicin induce [27, 28]. TNF-α also has been associated with aseptic activation of inflammasome NLRP3 [40] which mediates several major chronic diseases including arteriosclerosis, type 2 diabetes and Alzheimer disease [41]. Our study suggests that GPBP secretion is part of a novel TNF-α-EMT-dependent pathogenesis underlying these various human disorders.

In conclusion, we demonstrate that the novel drug candidate T12 targets mesenchymal GPBP (i.e. cGPBP) to disrupt the EMT-based chemoresistance of solid tumors including lung and breast cancer. Monitoring of cGPBP levels by EMTEST will allow selection of patients likely to respond to T12 and permit prediction of metastases formation for early treatment. Moreover, low-dose doxorubicin could be used to sensitize to T12 solid tumors and metastases including those in the brain since our evidence indicates T12 crosses blood-brain barrier. Dosage of doxorubicin for sensitization could be adjusted using EMTEST to prevent doxorubicin toxicity. Finally, the lack of toxicity, mutagenicity, and cross-inhibition of other protein kinases at therapeutic doses suggest the potential of T12 to prevent malignancy in patients with existing tumors or at risk to develop cancer.

## MATERIALS AND METHODS

### Ethical statement

Human samples were obtained from Hospital Clinico Universitario, Hospital General Universitario and Hospital Quirónsalud of Valencia, under the guidelines and approval of the corresponding Ethical Committees for Clinical Research. All human samples were collected with the informed consent of patients.

Investigation with animals has been conducted in accordance with the ethical standards and according to the Declaration of Helsinki and to national and international guidelines, and has been approved by the Animal Welfare Ethics Committee of the University of Valencia Animal Facility.

### Study design

The aim of this study was to demonstrate that specific targeting of the collagen IV that frames the microenvironment of chemoresistant tumor cells is a reliable and safe approach to reduce tumor load. The study covers three main concepts: 1) characterization of exportable GPBP as a pivotal regulator of tumor cell microenvironment; 2) rational design of inhibitors and selection of a drug candidate (T12); and 3) characterization of T12 pharmacological activity *in vitro* (autophosphorylation), *ex vivo* (3D cultures) and *in vivo* (xenograft) models.

### Yeast two-hybrid assays

Production of pGBT9-FLAG-GPBP-2 has been reported [5]. cDNA fragments representing GPBP deletion mutants were inserted into pGAD424 vector (Clontech).

### Expression and purification of recombinant GPBP

Yeast and mammalian FLAG-GPBP production has been reported [3]. Insect FLAG-GPBP proteins were produced in Sf9 cells using Bac-to-Bac^®^ Baculovirus Expression System (Thermo Fisher Scientific) and purified from culture media (extracellular) or cell lysate (intracellular) using anti-FLAG Affinity Gel (Sigma-Aldrich) column and FLAG peptide following manufacturer’s instructions.

### Size exclusion chromatography (SEC)

Fast protein liquid chromatography (FPLC)-SEC were performed using a Superdex 200 column (GE Healthcare) and high-performance liquid chromatography (HPLC)-SEC using a TSKgel® G4000SW column (Sigma-Aldrich).

### EMTEST and indirect ELISA

For preclinical EMTEST, plates (96-well) were coated with 1 μg/mL N26 in phosphate-buffered saline (PBS), blocked with 3% bovine serum albumin (BSA) in Tris-buffered saline with Tween-20 (TBST) (blocking buffer), incubated with calibrators (recombinant GPBP) diluted in blocking buffer or with plasma samples diluted 1:5 in TBST, both mixtures containing the horseradish peroxidase (HRP)-labeled detection antibody (N27-HRP, 1 μg/mL). Development was performed with the QuantaBlu™ Fluorogenic Peroxidase Substrate Kit (Thermo Fisher Scientific) and fluorescence detected with a SpectraMax GeminiXPS plate reader (Molecular Devices). Detection limit was established at 1 ng/mL. ^4^

For clinical EMTEST, single-chain variable fragment (scFv)N26 and HRP-labeled FabN27 were used for capture and detection, respectively.

When indicated, FPLC-SEC fractions were used to coat the plates and bound GPBP detected with a mixture of anti-GPBP mAb and anti-mouse IgG-HRP.

### Immunoprecipitation

N26, scFvN26 or scFvN27 was conjugated to cyanogen bromide-activated-Sepharose^®^ 4B beads (Sigma) and used to purify GPBP from plasma or culture media.

### *In vitro* phosphorylation assays

Purified recombinant and native GPBP polypeptides were phosphorylated and analyzed essentially as previously described [3].

### Collagen IV extraction and digestion

The 3D cultures were disrupted by sonication (1 min half power, 77 Joules) in PBS supplemented with Halt Protease and Phosphatase Inhibitor Cocktail (Thermo Fisher Scientific), centrifuged (20,000 × g, 1 h, 4° C) and pellets extracted with 0.1 M acetic acid pH 4 and analyzed by WB. When indicated, pellets were sequentially rinsed by centrifugation (20,000 × g for 30 min, 4° C) with 1 M NaCl, 50 mM Tris-HCl and 10 mM Tris-HCl, at pH 7.5; dispersed in 5 mM CaCl_2_, 5 mM benzamidine, 25 mM 6-aminocaproic acid, 0.4 mM phenylmethylsulfonyl fluoride (PMSF) and subjected to digestion with 50 μg/mL bacterial collagenase (Worthington Biochemical Corporation) for 24 h at 37° C under stirring. The mixtures were centrifuged (20,000 × g for 10 min at 4° C) and supernatants analyzed by WB.

### Cell cultures

All mammalian cell lines were from ATCC collection except hTERT-RPE-1 and hTERT-BJ1 (Clontech) and A7C11 (The Wistar Institute). Insect Sf9 cells (Thermo Fisher Scientific) were cultured in Sf-900™ II SFM media from the same supplier supplemented with 1% Pluronic^®^ F-68 (Sigma-Aldrich); 4T1 and A7C11 in RPMI1640 (Lonza) supplemented with 10% fetal bovine serum (FBS); LLC, RAW264.7, hTERT-BJ1, NMuMG and HEK 293 in high-glucose (4.5 g/L) Dulbeccoʼs Modified Eagle Medium (DMEM) (Lonza) supplemented with 10% FBS. NMuMG were also supplemented with 10 μg/mL insulin; A427, A549 and hTERT-RPE-1 in DMEM-F12 (Lonza) supplemented with 15 mM Hepes, 2.5 mM L-Gln and 10% FBS. All the media contained 100 U/mL penicillin and 100 μg/mL streptomycin.

### Cell line production

A549 cultures were transfected either with pEYFP-N1 (Clontech) or pEYFP-N1-GPBP expressing EYFP or GPBP-EYFP, respectively. Cells were selected with 400 mg/L of geneticin and individual cells isolated with a High-Speed Cell Sorter MoFlo (Beckman-Coulter) using intrinsic fluorescence and further cultured. Recombinant protein expression of selected clones was assessed by IF and WB.

A549 cells were cultured with 1 µM doxorubicin and the medium was changed every 48 h. When necessary the concentration of doxorubicin was lowered to 0.2 µM to prevent cell cycle arrest. After several weeks, doxorubicin IC50 was determined with alamarBlue^®^ to confirm doxorubicin resistance (A549-DR).

4T1 cells were co-transfected with plasmids expressing Cas9 and a guide RNA (Sigma-Aldrich) targeting GPBP exclusive sequence in exon 11 (CCCTATAGTCGCTCTTCCTCCA) to generate 4T1 GPBP^–/–^.

### 3D cultures and EMT induction

A549 spheroids were obtained by sequential culturing in hanging droplets and ultra-low binding plates essentially a previously described [18]. EMT was induced with 10 ng/mL TNF-α and 2 ng/mL TGF-β (Thermo Fisher Scientific) for 48 h. RAW 264.7, 4T1 and A7C11 cells formed spheroids when seeded in ultra-low binding plates.

### Cell culture transfection

Lipofectamine 2000 (Thermo Fisher Scientific) was used for cell culture transfection.

### RNA extraction and gene expression analysis

RNA was extracted with ilustra RNAspin Mini (GE Healthcare). RT was performed with the High Capacity cDNA Reverse Transcription Kit (Applied Biosystems) and qPCR was done using TaqMan^®^ Gene Expression Master Mix, specific TaqMan^®^ Gene Expression Assays and a StepOnePlus Real-Time PCR system device (Applied Biosystems). *HPRT1/Hprt* was the normalizer, the reference mean was set at 1 and reference SD was used for statistical purposes. Relative quantity (RQ) was calculated with the ∆∆C_T_ method.

### RNA interference

Silencer^®^ Select siRNAs for *COL4A3BP* and *COL4A1* and Silencer^®^ Select Negative Control siRNA No. 1 (Thermo Fisher Scientific) were used to transfect A549 cells.

### Flow cytometry analysis

Doxorubicin fluorescence was measured using FL2 channel of a BD LSRFortessa cytometer. Side population (SP) was identified using Hoechst 33342 (Thermo Fisher Scientific), verapamil and fumitremorgin C (Sigma-Aldrich) following standard procedures.

### Cell viability assays

#### Dose response analysis

Cell viability was assessed using alamarBlue^®^ (Thermo Fisher Scientific) and adherent 96-well plates.

#### Cytotoxicity and apoptosis assays

Were performed either with CytoTox-Glo™ Cytotoxicity Assay or ApoTox-Glo™ Triplex Assay (Promega). For these purposes, cells were seeded on opaque-wall, clear-bottom 96-well culture plates (2,500 cells/well), allowed to settle during 4 h and treated for 40 h.

#### Spheroids cell viability

Culture media were analyzed with Lactate Dehydrogenase Activity Assay Kit (Sigma-Aldrich).

### Mouse models

#### A549 mouse-model

A549 cells (3 x 10^6^) were suspended in 1:1 culture medium: Matrigel® (Corning) and injected into the right flank of 8-week-old athymic NMRI-Foxn1^nu^/Foxn1^nu^ male mice (Janvier). Developing tumors were periodically measured with a digital caliper and volumes calculated with the formula Volume = (Length × Width^2^)/2. When tumors reached desired volume, mice were randomly separated and treated with: vehicle (Control), doxorubicin (4 mg/kg/week, one intraperitoneal injection), T12 (0.1 mg/mL diluted in drinking water, daily), hN26 (1 mg/kg/week, one intraperitoneal injection) or the indicated combinations thereof. T12 plasma levels were determined in selected mice at the end of treatments following procedures described in the corresponding toxicokinetic report (Auxiliary Supplementary Material).

#### 4T1 mouse-model

4T1 cells (10^4^) were injected into the 4th right mammary fat pad of 4-week-old Balb/c female mice suspended in PBS, and either left untreated or treated from day 0 (inoculation day) with T12 or with N26 as above. When indicated, mice at day-9 were left untreated (control) or similarly treated with T12 and/or doxorubicin, or mice at day-16 were subjected to surgery to remove primary tumor and T12 treatment initiated and maintained until end-point criteria. In mice, which did not reach end-point criteria metastases were monitored by (micro) PET-CT and treatments modified (see Supplementary Table 2). Where indicated, blood was collected and seeded in the presence of 6-thioguanine (60 µM), c4T1 cells selected and further cultured. Mice were sacrificed, lungs excised and stained with Bouin’s solution and metastases counted.

#### LLC mouse-model

LLC cells (10^4^) suspended in PBS were injected into the right flank of 8-week-old GPBP^−/−^ and wild type C57BL/6 mice. After 28 days, mice were sacrificed and the tumor was removed if present. Metastases in lungs were counted as above.

All the animal studies were authorized and performed according to the guidelines of the Animal Welfare Ethics Committee which include end-point criteria. An independent veterinarian determined when mice met end-point criteria in survival assays.

### Histochemistry and immunohistochemistry

Tumors were fixed in 10% formalin, embedded in paraffin, and sliced on an electronic rotary microtome (Microm) to generate sections for standard histochemical and immunohistochemical procedures.

### Immunofluorescence, bioT12 staining and confocal microscopy

Spheroids of A549 cells were fixed with 4% formaldehyde (30 min), washed, permeabilized with 0.2% Triton X-100 (5 min) and blocked with 3% BSA (30 min). Then spheroids were incubated for 16 h at 4° C with the corresponding fluorophore-labeled primary antibodies diluted in blocking solution using gentle rocking. Finally, spheroids were recovered by centrifugation (100 × g, 5 min), washed and mounted for observation. PBS was the diluent in all solutions.

HEK 293 cells transfected with pc-GPBP-Ala^132^ construct were similarly washed, fixed, permeabilized and blocked as above. Cells were incubated with suitable primary antibodies at 37° C in humid chamber (1.5 h), washed with PBS and incubated with fluorophore-labelled secondary antibodies, washed again and mounted for observation.

Frozen tissue samples were processed essentially as described elsewhere [12]. For paraffin-embedded sample processing, sections were heated at 60° C (30 min), paraffin was eliminated with xylol washes (3 × 10 min), and sections were rehydrated with 1-min washes with decreasing ethanol concentrations (100%–90%–70%) and finally with tap and distilled water. Then sections were autoclaved (3 min, 1.5 atmospheres) in low-pH citrate buffer, allowed to rest at room temperature (15 min) and rinsed with water, and subsequently blocked with horse serum (20 min).

Blocked sections were incubated with primary antibodies diluted in EnVision Flex Antibody Diluent (Dako) overnight at 4° C in humid chamber, washed with PBS (3 × 5 min) and incubated with fluorophore-labeled secondary antibodies diluted in PBS (45 min, room temperature, darkness). Sections were then washed with PBS and mounted for observation. For some purposes, fluorophore-labeled primary antibodies were used.

When indicated, Alexa Fluor 488-streptavidin (Thermo Fisher Scientific) was diluted 1:500 in EnVision Flex Antibody Diluent (Dako) containing 1 μM bioT12 and preincubated for 2 h. In some instances, additional fluorophore-labeled antibodies were also added to the mixture. Frozen specimens were incubated overnight with staining mixtures in humid chamber at 4° C, washed and mounted for observation. BioT12 binding specificity was assessed by competing labelling with an excess of T12.

Anti-α1α2(IV) (Merck Millipore) and anti-GPBP (N27) were labeled with Alexa Fluor^®^ 647 and 546 Antibody Labeling Kit (Thermo Fisher Scientific), respectively.

Stained samples were analyzed with either an Olympus FV1000 confocal microscope assembled on a motorized inverted IX8 microscope or a TCS-SP2 laser-scanning confocal spectral microscope (Leica) assembled to a Leica DM1RB inverted microscope.

For some purposes, fluorescence intensity (FI) was measured in five independent fields essentially as described [42] using Photoshop^®^ software, and expressed as mean ± SD. The layer displaying the highest FI of each field was used for quantification.

### Synthesis of terphenyls

The synthesis of T12, and other related terphenyls, has been previously described.^2^ The synthesis of bioT12 and T43 are described in Supplementary Schemes 1 and 2, respectively.

### Statistics

Prism 5.0 software (GraphPad Software, San Diego, CA) was used for all calculations. In all analyses “*n*” stands for sample size per group: number of independent assays or mice unless indicated that a representative assay is being shown, in which case, refers to number of technical repetitions.

An extended version of this section is included in Supplementary Materials.

## SUPPLEMENTARY MATERIALS FIGURES AND TABLES






