# Supplementary material for: Selective targeting of collagen IV in the cancer cell microenvironment reduces tumor burden
Source: Oncotarget. 2018 Jan 19;9(13):11020–45. doi: 10.18632/oncotarget.24280 (PMC5834258; doi:10.18632/oncotarget.24280)
Supplement: Supplementary file 1 [file oncotarget-09-11020-s001.pdf]

Selective targeting of collagen IV in the cancer cell microenvironment reduces tumor burden

SUPPLEMENTARY MATERIALS

Antibodies

Mouse monoclonal antibodies (mAb) to Goodpasture antigen binding protein (GPBP) used for Western blot (WB), immunoprecipitation, immunofluorescence (IF) and EMTEST were developed by Fibrostatin, S.L.[4]. To analyze fast protein liquid chromatography (FPLC) fractions, detection was performed using a pool of mAb anti-GPBP: N4, N10, N11, N12, N27 and e11-2.

Sources of other antibodies: mouse mAb anti-E-cadherin, rabbit mAb anti-vimentin, rabbit polyclonal antibodies (pAb) anti-active caspase 3, rabbit pAb anti-Golgin 97 and rabbit pAb anti-mannose-6-phospate receptor (Abcam). Mouse mAb anti-tubulin, mouse mAb anti-FLAG and mouse mAb anti-FLAG-FITC (Sigma-Aldrich). Mouse mAb anti-GAPDH (kind gift of Erwin Knecht). Rabbit pAb anti-PARP-1, goat pAb anti-E-cadherin, rabbit pAb anti-p53 and goat pAb anti-collagen I (Santa Cruz Biotechnology). Goat pAb anti-α1α2(IV) antibodies (Merck Millipore). Anti-collagen IV mouse mAb 202 reacting with the NC1 domain of all six collagen IV chains (Fibrostatin). Rabbit pAb reacting with triple helical region (residues 21–70) of both human and mouse α5(IV) chain (LSBio). Mouse mAb anti-αSMA (Dako). Rabbit pAb anti-phospho-p53 (Ser15) (Cell Signaling Technology). Alexa Fluor-labeled secondary antibodies (Molecular Probes).

Circular dichroism (CD)

All measurements were carried out on a Jasco J-720 CD instrument, using a Neslab RTE 110 water-bath and a temperature controller, essentially as described elsewhere [43]. CD spectra were the average of a series of three to seven scans made at 0.2 nm intervals, recorded at 25° C. CD spectra of the buffer were used as baseline in all the experiments. Elliptical measurements are reported as mean residue ellipticity [θ] (deg cm^2 $dmol^{-1}$); the limits of error of measurements at 222 nm were ± 200 (deg cm^2 $dmol^{-1}$).

Peptide synthesis

Peptides Q2 (CH_3CO-LATLSHCIELMVKR-NH_2) and Q2rd (CH_3CO-VLMASLETLCRIHK-NH_2) were synthesized by solid-phase methods using N-(9-fluorenyl) methoxycarbonyl (Fmoc) chemistry and purified by preparative high-performance liquid chromatography (HPLC) of reverse phase. Matrix-assisted laser desorption ionization-time-of-flight mass spectrometry was used to confirm peptide identity.

Plasmid constructs for yeast two-hybrid assays

The individual cDNA encoding GPBP deletion mutants were obtained by polymerase chain reaction (PCR) using *Pfu* DNA polymerase (Agilent Technologies), pGBT9-FLAG-GPBP construct [5] and the following pair of primers (see sequences below): GPBP (1–614) ON-pGBT9-1m and ON-GPBP-0.2; GPBP (1–604) ON-pGBT9-1m and ON-GPBP-0.4; GPBP (1–586) ON-pGBT9-1m and ON-GPBP-0.5; GPBP (1–498) ON-pGBT9-1m and ON-GPBP-1.5; GPBP (1–448) ON-pGBT9-1m and ON-GPBP-P2; GPBP (1–370) ON-pGBT9-1m and ON-N-t EX26-c; GPBP (1–264) ON-pGBT9-1m and ON-GPBP-P6; GPBP (1–259) ON-pGBT9-1m and ON-GPBP-P6.1; GPBP (1–254) ON-pGBT9-1m and ON-GPBP-P6.2; GPBP (1–249) ON-pGBT9-1m and ON-GPBP-P6.3; GPBP (1–244) ON-pGBT9-1m and ON-GPBP-P7; GPBP (379–624) ON-C-t EX26-m and ON-pGBT9-1c. The PCR products were purified (GenElute PCR Clean-Up Kit, Sigma-Aldrich), digested with *Eco*RI and *Sma*I endonucleases and ligated with pGAD424 vector previously linearized with the same enzymes to obtain the corresponding pGAD424-based constructs.

Others

pGAD424-GPBP (273–624), pGBT9-FLAG-GPBP was digested with *Pvu*II and *Ngo*MI and the corresponding

DNA fragment was purified and cloned into pBRIDGE vector (Clontech) previously digested with *Sma*I and *Ngo* MI. The resulting construct was digested with *EcoR*I and *Sal*I and the released DNA fragment further ligated into pGAD424 previously digested with the same enzymes.

pGAD424-FLAG-GPBP (1–129), pGBT9-FLAG-GPBP was digested with *EcoR*I and *Sal*I and the released DNA fragment was ligated with pGAD424 previously digested with the same enzymes.

pGAD424-GPBP (130–624), pGBT9-FLAG-GPBP was digested with *Sal*I and the released fragment ligated with *Sal*I-linearized pGAD424.

pGAD424-FLAG-GPBP-E264A expressing GPBP-Ala^{264} fused to the GAL4 activation domain was produced with Transformer™ Site-Directed Mutagenesis Kit (Clontech) using mutagenic primer ON-GPBP-E264A and selection primer ON-pGAD SEL, following manufacturer's instructions.

pGBT9-GIP130, the cDNA encoding for I20 truncated GIP130 polypeptide [21] was cloned into pGBT9 using PCR procedures.

Oligonucleotides

ON-pGBT9-1m: 5′-AATAAGTGCGACATCATC-3′: ON-pGBT9-1c: 5′-TCATAAGAAATTCGCCCG-3′: ON-N-t EX26-c: 5′-AAACCCGGGTTACTTTGGACA AATCTATGTGT-3′: ON-C-t EX26-m: 5′-AAAGAATT CGTTGAAGAGATGGTGCAGAAG-3′: ON-GPBP-0.2: 5′-AAAGAATTCCCGGGTTATTGGACGTAAGAAGT AAAACG-3′: ON-GPBP-P0.4: 5′-AAAGAATTCCCGGG TTATTTAGGATACTCTCGCTT-3′: ON-GPBP-P0.5: 5′-A AAGAATTCCCGGGTTATCCAGGGTTCACATTAGC-3′: ON-GPBP-P1.5: 5′-AAAGAATTCCCGGGTTACACC CTCTTGTGTGTTTG-3′: ON-GPBP-P2: 5′-AAAGAA TTCCCGGGTTACATGGGTAGCTTTTAAAG-3′: ON-GPBP-P6: 5′-AAAGAATTCCCGGGTTATTCAATACAA TGAGAAAG-3′: ON-GPBP-P6.1: 5′-AAAGAATTCCC GGGTTAAAGTGTTGCAAGGATTCC-3′: ON-GPBP-P6.2: 5′-AAAGAATTCCCGGGTTATCCAGCAGTAGT TGCTTT-3′: ON-GPBP-P6.3: 5′-AAAGAATTCCCGGG TTATTTAAAAGTTATCGCTTCCCC-3′: ON-GPBP-P7: 5′-AAAGAATTCCCGGGTTATTCCCCTTTAAAGTCT AT-3′: ON-GPBP-E264A: 5′-CAACACTTTCTCATTGT ATTGCACTAATGGTTAAACGTGAGG-3′: ON-pGAD SEL: 5′-GTGCCACCTGATATCTAAGAAACC-3′.

Other plasmid constructs

pHIL-D2-FLAG-GPBP-E264A expressing FLAG-GPBP-Ala^{264} mutant was produced by digestion of pGAD424-FLAG-GPBP-E264A with *EcoR*I and subsequent cloning of the released cDNA into pHIL-D2 vector (Thermo Fisher Scientific).

Production of pc-GPBP, a pcDNA3 (Invitrogen)-based construct used for expressing FLAG-GPBP in mammalian cells, has been described elsewhere [3]. The derived point mutated pc-GPBP-Ala^{132} was obtained by PCR-based site-directed mutagenesis.

For recombinant expression of FLAG-GPBP, BM40-FLAG-GPBP and BM40-FLAG-GPBPAla^{132} in Sf9 insect cells, the corresponding cDNAs were cloned into pFastBac1 vector. BM40- and FLAG-containing cDNAs were obtained by PCR from pc-GPBP and pc-GPBP-Ala^{132}.

Immunoprecipitation

Media from A427 and A549 cell cultures were immunoprecipitated with 50 µL of Sepharose-conjugated single chain variable fragment of mAb N26 (scFvN26), overnight with gentle rocking at 4° C. Beads were recovered by centrifugation (500 × g, 10 min, 4° C) and washed six times alternating Tris-buffered saline (TBS) and TBS-Tween 20 (TBST) (1 mL per wash). Elution was performed with TBS containing 0.1 mg/mL of peptide PS132 [CLRRHGS(P)MVSLV] where the serine representing Ser^{132} in GPBP sequence (underlined) is phosphorylated. Elution fractions were pooled and the peptide eliminated by alternating ultrafiltration (Amicon® Ultra Centrifugal filters 10 K, Merck Millipore) and phosphate-buffered saline (PBS)-dilution steps. Purified materials were stored at –80° C until use.

For some purposes, supernatants of HEK 293 cultures transfected with pc-GPBP, pc-GPBP-Ala132 or with pcDNA3 vector were immunoprecipitated with ANTI-FLAG® M1 Agarose Affinity Gel, and bound material was eluted with FLAG peptide (0.1 mg/mL in TBS) and analyzed by WB.

Where indicated, plasma samples from non-small cell lung cancer (NSCLC) and Goodpasture patients and from healthy donors were immunoprecipitated with anti-GPBP scFvN26, bound material eluted with peptide PS132, subjected to serial dilution (with TBS) and concentration (Amicon® Ultra Centrifugal filters 10 K, Merck Millipore) cycles and used for *in vitro* kinase assays. In some cases, plasma samples from NSCLC patients were immunoprecipitated with anti-GPBP scFvN27, and bound material was eluted with peptide 43C [LINEEEFFDAVEAALDRQ] (0.1 mg/mL in TBS).

Size exclusion chromatography (SEC)

FPLC-SEC analyses were carried out with a Superdex 200 column and an ÄKTApurifier (GE Healthcare) using TBS as mobile phase at 0.5 mL/min and monitoring the eluted material by following absorbance at 280 nm. For purified Sf9-expressed FLAG-GPBP typically 150–250 µg were analyzed. Where indicated, 125 µg of Sf9-expressed extracellular FLAG-GPBP or of BSA were incubated with or without 10 mM T12 for 15 min at room temperature and analyzed by FPLC-SEC. Native GPBP immunopurified from 2.5 L of culture media of A549 or A427 was similarly analyzed and fractions (500 µL) stored

at −80° C until used for enzyme-linked immunosorbent assay (ELISA) purposes. When indicated, 25–75 µg of recombinant yeast FLAG-GPBP proteins were analyzed by HPLC using TSK-G4000SW column (Sigma-Aldrich) and eluted with 50 mM Tris-HCl, pH 7.5, 150 mM NaCl at 1 mL/min and monitoring the eluted material by following absorbance at 220 nm.

RNA extraction and quantitative PCR (qPCR)

Total RNA was extracted with illustra RNAspin Mini (GE Healthcare) from cells or tumors following manufacturer's instructions. Reverse transcription of 1 µg RNA samples was performed with High Capacity cDNA Reverse Transcription Kit (Applied Biosystems).

For qPCR, 1.5 µl of cDNA were mixed with TaqMan® Gene Expression Master Mix (Applied Biosystems) and the specific TaqMan® Gene Expression Assays (Applied Biosystems). The TaqMan® Gene Expression Assays for the analyzed genes were selected according to manufacturer's recommendations, except for *COL4A3BP-1*, for which a custom TaqMan® Gene Expression Assay was developed to specifically target *COL4A3BP* exon 11. Relative quantity (RQ) was calculated with the ΔΔCt method. PCR were performed using a StepOnePlus Real-Time PCR system (Applied Biosystems) in duplicate.

In vitro phosphorylation assays

Purified recombinant GPBP proteins (200 ng), or native immunopurified cGPBP were incubated in 25 mM disodium β-glycerophosphate, 8 mM $MgCl_2$, 0.5 mM ethylenediaminetetraacetic acid (EDTA), 0.5 mM ethylene glycol-bis(β-aminoethyl ether)-N,N,N′,N′-tetraacetic acid (EGTA), 1 mM dithiothreitol (DTT), 5 mM $MnCl_2$ and Q2, Q2rd, T12 or doxorubicin where indicated, during 10 min at 37° C with orbital agitation (350 rpm). Then $\gamma[^{32}P]$ ATP (Perkin Elmer) was added to a final concentration of 0.132 µM and reactions were allowed to proceed during 15 min at 37° C with orbital agitation (350 rpm). Reactions were stopped with sodium dodecyl sulfate polyacrylamide gel electrophoresis (SDS-PAGE) loading buffer, heated (95° C, 3 min) and analyzed by SDS-PAGE, electro-transference to polyvinylidene difluoride (PVDF) membrane (Merck Millipore) and autoradiography. Proteins were visualized with anti-FLAG (Sigma-Aldrich) or GPBP-specific antibodies and chemiluminescence (ECL, GE Healthcare).

Where indicated, purified FLAG-GPBP-Ala^{132} expressed in Sf9 and recombinant AMP-dependent protein kinase (AMPK) (Millipore) were used. In phosphorylation assays with AMPK, 100 µM unlabeled ATP was included in reactions along with 0.132 µM $\gamma[^{32}P]$ATP.

Western blot

Cells were lysed in TBS supplemented with 1% Triton X-100, 0.1% SDS, 1× Halt Protease and Phosphatase Inhibitor Cocktail (Thermo Fisher Scientific). Lysates were centrifuged (16,000 × g, 5 min, 4° C), supernatants collected and protein concentration determined (Bio-Rad Protein Assay). Samples were subjected to SDS-PAGE using a Mini-Protean System and electro-transferred onto PVDF membrane (Bio-Rad). Membranes were blocked in 5% skim milk in TBST and incubated with suitable primary antibodies and horseradish peroxidase (HRP)-labelled secondary antibodies or with HRP-labelled primary antibodies. When indicated, biotin-labelled primary antibodies and neutravidin-HRP (Thermo Fisher Scientific) were used. Development was performed by chemiluminescence (ECL Prime, GE Healthcare) using an ImageQuant LAS 4000 Mini system (GE Healthcare).

Where indicated, autoradiography was performed before blocking step and antibody binding detected by chemiluminescence with Hyperfilm (GE Healthcare).

Signals were quantified using ImageQuantTL (GE Healthcare) or WCIF ImageJ software.

Recombinant GPBP expression and purification

Recombinant expression in Sf9 insect cells was performed with the Bac-to-Bac® Baculovirus Expression System (Thermo Fisher Scientific). For these purposes, pFastBac 1-based plasmid constructs containing FLAG-GPBP, BM40-FLAG-GPBP and BM40-FLAG-GPBP-Ala^{132} cDNAs were used to transform *Escherichia coli* DH10Bac™, where cDNAs are transposed into a bacmid genome. The resulting recombinant bacmids were isolated and used to transfect Sf9 cells and obtain virus. Initial viral particles were used to infect freshly cultured Sf9 cells for amplification. In infections, Sf9 cells were grown in SF900II SFM media supplemented with antibiotics and 1% Pluronic (Sigma-Aldrich) in suspension in spin flasks at 120 rpm and 27.5° C. Then cells were brought to a 300,000-cells/mL density and infected at a multiplicity of infection (MOI) of two upon reaching a 1,500,000-cells/mL density. Amplified virus was used for large scale Sf9 infection. FLAG-polypeptides expressed with BM40 signal peptide suffered forced secretion to the culture media, while FLAG-polypeptides expressed without BM40 underwent spontaneous secretion. Extracellular FLAG-polypeptides were purified from culture media 72 h after large scale infection. Briefly, media were centrifuged (500 × g, 10 min) to pellet insect cells and supernatants were further centrifuged (160,000 × g, 1 h, 4° C) to pellet viral particles and cell debris, filtered (45 µm-pore size) and extracted with an ANTI-FLAG® M1 Agarose Affinity Gel (Sigma-Aldrich) column. The column was extensively

washed with TBS and bound FLAG-GPBP eluted with FLAG peptide (0.1 mg/mL in TBS). FLAG peptide was eliminated by alternating ultrafiltration (Amicon® Ultra Centrifugal filters 10 K, Merck Millipore) and TBS dilution steps. Finally, the purified protein was quantified (Bio-Rad Protein Assay) and stored at −80° C. To obtain purified intracellular FLAG-polypeptides, infected Sf9 cells were lysed in TBS supplemented with 0.1% Triton X-100, 1 mM phenylmethylsulfonyl fluoride (PMSF), 10 µg/mL leupeptin and 10 µg/mL benzamidine at 4° C during 30 min. Lysates were centrifuged (16,000 × g, 1 h, 4° C) and supernatants filtered with 45 µm-pore-size filter and purified as above.

Yeast FLAG-GPBP and FLAG-GPBP-Ala^{264} were obtained essentially as previously described [3].

Bacterial poly-His-GPBP was obtained essentially as previously described.[3]

Interference assays of GPBP-Collagen IV binding

Briefly, 96-well ELISA plates were coated overnight with poly-His-GPBP in PBS at 4° C. Blocking was performed with 3% BSA in PBS (1 h, room temperature). After blocking plates were incubated with FLAG-α3NC1 [44] in TBST in the absence (control) or presence of N26 for 2 h at 37° C. Bound FLAG-α3NC1 was detected by incubation with anti-FLAG M2-HRP (1 h, room temperature) and development with SIGMAFAST™ OPD using a LKB microplate reader. All polypeptides were used at a 1 µg/mL concentration. Washings with TBST were performed between steps.

Isolation and characterization of 4T1 $GPBP^{-/-}$ clones

Clones were selected by limiting dilution and screened by IF with mAb e11-2 (GPBP) and N27 [GPBP and GPBP-2/ceramide transfer protein (CERT)]. Selected clones were further characterized by WB with the same antibodies. To characterize the mutations introduced in *Col4a3bp* exon 11, genomic PCR products encompassing the target sequence were ligated into a pCR™ plasmid (TA Cloning, Thermo Fisher Scientific), propagated in *E. coli*, and individual clones were isolated and sequenced.

Flow cytometry

Study of doxorubicin effects on side population (SP) and non-SP

A549 cells (1×10^6) were cultured during 24 h and treated with 1 µM doxorubicin and/or 50 µM T12 for additional 36–42 h. Cells were twice washed with PBS, trypsinized, dispersed in Dulbecco's Modified Eagle's medium (DMEM)-F12, 10% fetal bovine serum (FBS), 10 mM HEPES pH 7.4 (suspension medium), collected by centrifugation (300 × g, 5 min, 4° C), further dispersed in suspension medium (10^6 cells/mL), added with Hoechst 33342 (Thermo Fisher Scientific) at 5 µg/mL and incubated at 37° C in a shaking water-bath for 90 min in darkness. To aliquots of each sample, ABC transporter inhibitors verapamil (50 µM) and fumitremorgin C (10 µM) (Sigma-Aldrich) were added along with Hoechst 33342 to set the SP gate. After incubation, cells were centrifuged (300 × g, 5 min, 4° C), dispersed in ice-cold suspension medium and analyzed by flow cytometry using a BD LSRFortessa™ cytometer to assess both, doxorubicin and Hoechst 33342 fluorescence. Data were analyzed with FlowJo version 10.0.

Analysis of doxorubicin extrusion

A549 cells were incubated with 6.9 µM doxorubicin in culture medium for 2 h, washed with PBS and incubated in doxorubicin-free culture medium for 6 additional hours. Cells were subsequently trypsinized and analyzed with a BD FACSVerse™ flow cytometer for assessment of doxorubicin content.

Cell cycle analysis

A549 cells were seeded on 6-well plates (7×10^4 cells/well) and cultured overnight. Cells were then treated with 0 or 50 µM T12 for 48 h and later culture media were centrifuged to collect detached cells while attached cells were trypsinized and suspended in PBS. At that point both cell fractions were pooled and centrifuged (500 × g, 10 min), and cell pellets were resuspended in PBS, centrifuged again and suspended in 1 mL ice-cold 70% ethanol added dropwise under vigorous vortexing. Ethanol-suspended cells were overnight stored (4° C), centrifuged (1500 × g, 10 min), twice washed with 1 mL of PBS, and finally suspended in 250 µL of PBS containing RNase (0.2 mg/mL) and incubated at 37° C for 1 h. Propidium iodide was added (10 µg/mL), cells incubated for 40 min in darkness and cell cycle analyzed with a BD FACSVerse™ flow cytometer and the ModFit LT software.

Cell proliferation assays

Were performed with the CellTrace™ Cell Proliferation Kit (Thermo Fisher Scientific) following manufacturer's instructions. Briefly, cells were seeded on 6-well plates (5×10^4 cells/well), cultured overnight, incubated with CFSE reagent for 20 min and treated for 72 h. Subsequently cultures were trypsinized and fixed by suspension in 4% paraformaldehyde in PBS for 20 min in darkness at room temperature. Next, cells were centrifuged (500 × g, 10 min), rinsed twice with PBS and analyzed with a BD FACSVerse™ flow cytometer.

Assessment of early and late apoptosis and necrosis

PE Annexin V Apoptosis Detection Kit I (BD Bioscience) was used with a BD FACSVerse™ flow cytometer.

Cell viability assays

Dose-response analysis

For IC50 determination of peptidomimetics, cells were seeded on 96-well culture plates (2,500 cells/well) and allowed to settle during 4 hours. Then cells were treated with individual compounds at concentrations ranging from 0 to 200 μM during 36–40 h. Subsequently, alamarBlue® reagent was added to wells and incubation maintained for 3 additional hours. Fluorescence was measured using 560EX nm/590EM nm filter settings with a SpectraMax GeminiXPS plate reader (Molecular Devices). Blank wells containing media were used to determine background fluorescence. Data processing and IC50 calculations were performed with SoftMax Pro software (Molecular Devices).

For assessment of the *ex vivo* activity of peptidomimetics (Supplementary Table 1), the half maximal inhibitory concentration (IC50) of doxorubicin for A549 cells was calculated with alamarBlue® reagent using doxorubicin concentrations ranging from 0 to 10 μM, in the absence or presence of the corresponding peptidomimetics at 50 μM-concentration. The *ex vivo* activity for each peptidomimetic was calculated by dividing the A549 doxorubicin IC50 in absence of peptidomimetic by that determined in presence of the peptidomimetic at 50 μM. For T12 similar analysis were performed at 10 and 25 μM.

To compare sensitivity to doxorubicin of A549 and doxorubicin-resistant A549 (A549-DR) cells, we performed dose-response analysis to doxorubicin with alamarBlue® as described above. In this case the fluorescence readings were transformed into cell numbers using a standard curve performed with wells where known cell numbers had been seeded 4 h before.

GPBP uptake by A549 cells

A549 cells were incubated with 1 μg/mL purified recombinant FLAG-tagged GPBP expressed in Sf9 cells in culture medium for 6 h, in presence of 40 μM of the AMPK inhibitor dorsomorphin or with the vehicle (DMSO). Then cells were washed with PBS and lysed in TBS supplemented with 0.5% Triton X-100, 1 mM PMSF, 10 μg/mL leupeptin during 30 min at 4° C. Lysates were cleared by centrifugation (16,000 × g, 5 min, 4° C) and recombinant GPBP extracted with anti-FLAG Affinity Gel (Sigma). Bound protein was eluted with FLAG peptide (0.1 mg/mL in TBS), concentrated with 10K Amicon Centrifugal Filter Units (Millipore) and used in phosphorylation assays.

Three-dimensional spheroid cultures and epithelial-to-mesenchymal transition (EMT) induction

Cells were grown on standard two-dimensional (2D) conditions to reach 80% confluence, and then trypsinized, dispersed in DMEM supplemented with 10% FBS and counted with an automated cell counter (Moxi Z, Orflo). The cell suspension was brought to 10^6 cells/mL, and 25 μL dropped onto the underside of a 10-cm tissue culture plate lid. Each lid was loaded with approximately 55 droplets and placed onto a tissue culture plate containing 6 mL of sterile PBS and incubated for 48 h to facilitate spheroid formation. Freshly formed spheroids were transferred into 6-well ultra-low binding plates (Nunclon Sphera, Thermo Fisher Scientific) and cultured in DMEM supplemented with 2% FBS. For EMT induction, spheroids were treated with 10 ng/mL tumor necrosis factor (TNF)-α and 2 ng/mL transforming growth factor (TGF)-β (Thermo Fisher Scientific) (TαTβ stimulation) for 48 h. Where indicated, spheroids were subjected to a second stimulation in fresh media.

Production of humanized N26 (hN26)

Production of hFabN26

Vectors pComb3XSS and pComb3XTT were kindly provided by The Barbas Laboratory (Scripps Research Institute, USA). The mRNA from N26 hybridoma was collected using TRI Reagent (Sigma-Aldrich) and total cDNA was synthesized using SuperScript III Reverse Transcriptase (Thermo Fisher Scientific). *E. coli* expression vector was constructed by cloning hFabN26 cDNA into pComb3XSS following three sequential steps of PCR amplification as previously described [45]. First, murine variable regions of the heavy chain (VH) and the light chain (VL) were amplified by PCR using cDNA of N26 hybridoma as template. Human constant regions of both the heavy chain domain 1 (CH1) and the light chain (CL) were amplified by PCR using vector pComb3XTT as the template. Second, equimolar quantities of VH and CH1 PCR products were used in the overlap PCR to create the heavy chain Fd (Fd), while equimolar quantities of VL and CL PCR products were used to create the light chain. Third, equimolar quantities of Fd and light chain PCR products were pooled in the overlap PCR to create the full chimeric Fab cDNA. Finally, chimeric Fab cDNA was cloned into pComb3XSS following single digestion of restriction endonuclease *Sfi*I.

The resulting library contained approximately 1.5×10^7 independent transformants. Three rounds of panning with immunoplates coated with 1 μg/mL FLAG-

GPBP were performed with the library, and the binding phages of each round were eluted by competition with FLAG-GPBP (0.1 mg/mL). Finally, a phage ELISA was performed and 34/94 (36.1%) of clones were positive for GPBP, demonstrating selection had occurred during biopanning.

Twelve clones were subjected to sequencing. Seven different hFabN26 cDNA sequences were found and the corresponding polypeptides were expressed and purified for affinity constant determination (Biacore, GE). Erlenmeyer flasks containing 1 L of lysogeny broth (LB), 100 µg/mL ampicillin and 2 % glucose were inoculated with 10 mL of overnight culture from a selected Fab-expressing *E. coli* clone. Clones were incubated at 37° C until an OD600 nm of 0.6 was reached and then induced with 1.5 mM isopropyl β-D-1-thiogalactopyranoside (IPTG). After overnight expression at 30º C, cells were collected by centrifugation, lysed by sonication in TBS with protease inhibitors and supernatants of cell lysates loaded onto G-protein superflow sepharose (ABT) column equilibrated with TBS, pH 7.4, washed with 5 volumes of TBS, pH 7.4. Individual Fabs were eluted in different fractions with 0.1 M Gly-HCl (pH 2.7) and rapidly neutralized with 1 M Tris-HCl (pH 8). Fractions containing Fabs were pooled and dialyzed against TBS, and quantified by ELISA prior to affinity measurement.

Surface plasmon resonance assays

The affinity of selected hFabN26 polypeptides to FLAG-GPBP was determined with the Biacore T100™ surface plasmon resonance (SPR) system (Biacore Inc., Piscataway, NJ), equipped with a CM5 sensor chip (Biacore AB, Uppsala, Sweden). For interaction analysis, reaction surfaces were generated by amine coupling of FLAG-GPBP to flow cell 2, using flow cell 1 as reference. CM5 chips were activated with N-ethyl-N'-(3-dimethylaminopropyl)-carbodiimide hydrochloride (EDC) and N-hydroxysuccinimide (NHS) following supplier's instructions. FLAG-GPBP was diluted into 10 mM sodium acetate pH 4.5 and injected over the activated chip at 30 µg/mL. Using variable flow time across individual chip channels, a range of FLAG-GPBP densities was achieved: 200–800 response units. The chip was then blocked with ethanolamine. Assays were performed at 37° C, with Biacore running buffer composed of HBS-EP (0.01 M HEPES, pH 7.4, 0.15 M NaCl, 3 mM EDTA, 0.005% Surfactant P20), a flow of 0.1 mL/min with 120 seconds of contact time and a dissociation time of 540 seconds. The regeneration step consisted of a wash with 20 mM NaOH for 45 seconds at the same flow. The kinetic assays were performed with purified hFabN26 polypeptides at different concentrations (50, 100, 150, 200 and 250 nM). Each run included at least two repeats of the 100 nM sample and three blanks without sample.

The kinetic data were obtained after analysis of sensograms performed with the BIAevaluation 2.0 software (GE Healthcare). Kinetic association (kOn) and dissociation (kOff) rates were obtained simultaneously by fitting the data to a 1:1 Langmuir binding model. Equilibrium dissociation constants (KD) were calculated. A Fab clone was selected according to its lowest KD and was used as template for scFVN26 and hN26 production.

scFv preparation

To generate scFvN26 we amplified by PCR the selected hFabN26 cDNA clone with suitable primers [45] to incorporate a flexible glycine-serine long linker between the VL and VH sequences, and then cloned the PCR product into *Sfi*I-digested pComb3XSS prokaryotic vector upstream of a poly-His tail. Later, the scFvN26 cDNA poly-His tailed was excised and further cloned into the pFastBac1 eukaryotic vector, under the BM40 exportation signal. Expression of scFvN26 was achieved in Sf9 insect cells as performed for FLAG-GPBP (see above). For purification purposes of secreted scFvN26, media from cultures were collected 72 h after infection and centrifuged (500 × g, 10 min) to pellet insect cells, and supernatants were further centrifuged (160,000 × g, 1 h, 4° C) to pellet viral particles. The pH of final supernatants was adjusted to 7.4 with PBS and tangential filtration with 10K filters (Spectrum Labs), and scFvN26 was purified by immobilized metal ion affinity chromatography (IMAC) with NTA agarose (ABT). Bound scFvN26 was eluted with 300 mM imidazole in PBS, pH 7.4. Elution fractions were pooled and imidazole was eliminated by alternating ultrafiltration (Amicon® Ultra Centrifugal filters 10 K, Merck Millipore) and PBS-dilution steps.

For generation of scFvN27 a similar procedure was carried out using N27 hybridoma.

Preparation of complete chimeric hN26 antibody

For expression of hN26 antibodies in Sf9 insect cells, the corresponding heavy and light chain cDNAs were cloned into the bicistronic pFastBac Dual vector (Thermo Fisher Scientific). First, the cDNA of hFabN26 heavy chain was PCR-amplified from pComb3X-based construct (see above) and cloned downstream in-frame with the cDNA of BM40 (MRAWIFFLLCLAGRALAA) eukaryotic exportation signal in the polyhedrin promoter-driven expression cassette of pFastBac Dual. Second, a synthetic cDNA of the Fc of a human IgG1 (GenScript) was cloned downstream in-frame with the hFabN26 heavy chain cDNA totalizing a full hN26 heavy chain cDNA. Third, the cDNA of the hFabN26 light chain was also PCR-amplified from the pComb3X-based construct and cloned downstream in-frame with BM40 cDNA in the PD10 promoter-driven expression cassette of pFastBac

Dual vector. The final construct co-expressing BM40-tagged heavy and light hN26 chains was transformed into DH10Bac competent cells and recombinant baculovirus were produced following the instructions manual (Bac-to-Bac® Baculovirus Expression System, Thermo Fisher Scientific). Media from cell cultures were removed 72 h after infection and hN26 protein purified using the G-protein affinity gel (ABT) per manufacturer's protocol. Prior to affinity purification, the media containing the recombinant mAb were centrifuged at 160,000 × g (1 h, 4° C) to remove baculovirus particles and cellular debris. The recombinant protein was eluted with 0.1 M Gly-HCl (pH 2.7) and rapidly neutralized with 1 M Tris-HCl (pH 8). In a typical purification, about 15 mg of soluble recombinant mAb exported to the media was obtained from each liter of infected cells.

The affinity of hN26 to human FLAG-GPBP was assessed by surface plasmon resonance as above and KD was 4.5 × 10e-10 (KD of N26 = 2.21 × 10e-9).

Preparation of FabN27

FabN27 used in clinical EMTEST was prepared from purified mAb N27 using Pierce Mouse IgG1 Fab and F(ab')2 Preparation Kit (Thermo Fisher Scientific) and subsequently labelled with HRP.

Micro positron emission tomography-computed tomography (PET-CT)

Studies were done in the microPET-CT service at the Central Unit for Research in Medicine of the University of Valencia, using an Albira ARS System (Oncovision). Mice were maintained under isoflurane anesthesia during the entire procedure. To assess *in vivo* glucose uptake, anesthetized mice received an intraperitoneal injection of 0.2–0.3 mCi of 2-deoxy-2-(^{18}F)fluoro-D-glucose (^{18}F-FDG) in 200 µL of PBS. Mice were transferred to the microPET-CT system and were kept immobilized for 40 min to facilitate ^{18}F-FDG distribution. Whole-body images were acquired: PET (15 min) and CT (7 min; 45 kV; 0.8 mA). Images were reconstructed using the system software. Radiation emission (kBq/cm^3) in regions of interest (nodules) was quantified using AMIDE free software.

T12 sodium salt

To formulate T12 as a sodium salt, solid T12 was suspended in 1 equivalent of 0.2 M NaOH solution and stirred at room temperature until the mixture became clear. The solution was freeze-dried and the resulting powder was stored in a desiccator.

Synthesis of bioT12 and T43

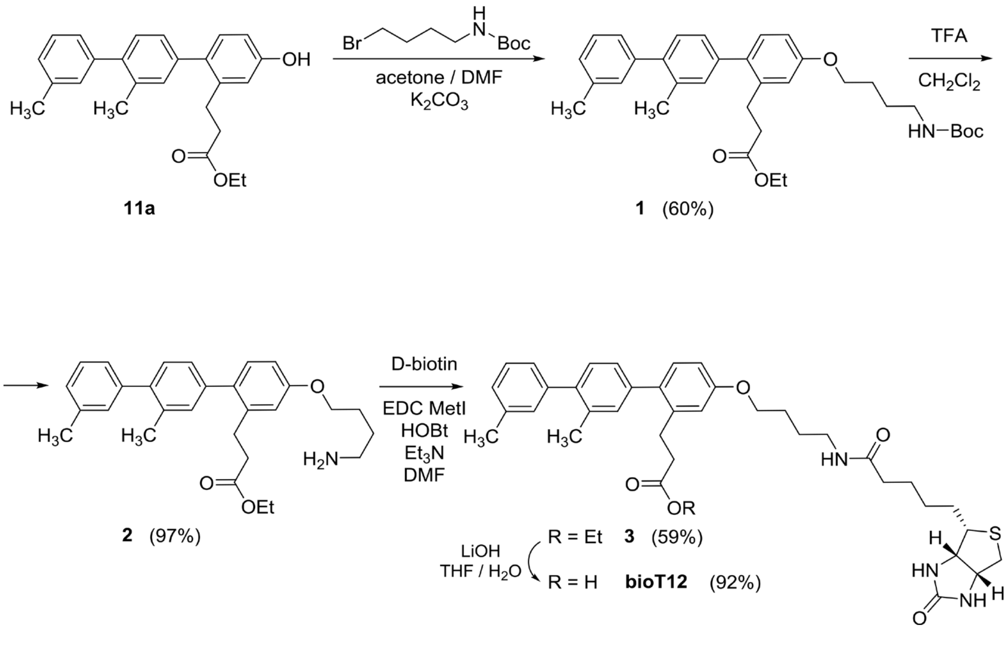

Supplementary Scheme 1: synthesis of bioT12, an adduct between compound 11a[2] and D-biotin with the use of commercial 4-(Boc-amino)butyl bromide as a linker.

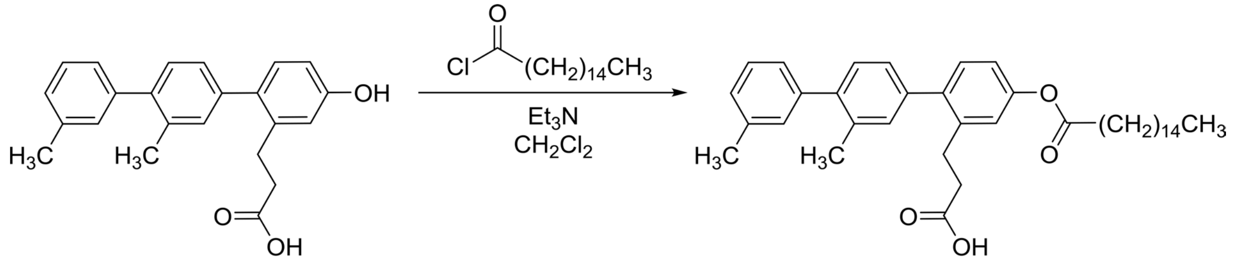

12a **T43** (15%)

Supplementary Scheme 2: synthesis of compound T43 from compound 12a.[2]

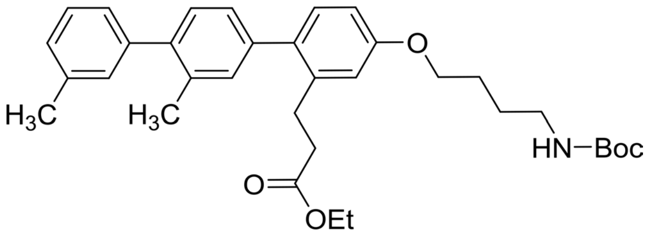

Production of: ethyl 3-(4-(4-((tert-butoxycarbonyl)amino)butoxy)-3′,3″-dimethyl-[1,1′:4′,1″-terphenyl]-2-yl) propanoate (compound 1). *Tert*-butyl (4-bromobutyl)carbamate (336 mg, 1.33 mmol) and potassium carbonate (230 mg, 1.67 mmol) were added to a microwave flask. Next, 250 mg (0.66 mmol) of compound **11a** were dissolved in 5 mL of acetone/dimethylformamide (DMF) (10:1) and added to the reaction mixture. The reaction was heated 2 h at 100° C in a microwave oven. The solvent was eliminated *in vacuo*, resuspended in water (10 mL) and extracted with ethyl acetate (AcOEt) (3 × 10 mL). The organic layers were dried over Na_2SO_4, filtered and concentrated. The crude was purified by flash chromatography on silica gel (hexane/AcOEt, 7:1) to afford 220 mg of compound **1** as a colorless oil. Yield 60%. ^{1}H nuclear magnetic resonance (NMR) (300 MHz, $CDCl_3$) δ ppm: 7.36–7.29 (m, 1H), 7.27–7.12 (m, 7H), 6.85 (d, J = 2.4 Hz, 1H), 6.81 (dd, J_1 = 8.4 Hz, J_2 = 2.7 Hz, 1H), 4.66 (br s, 1H), 4.10 (q, J = 7.1 Hz, 2H), 4.02 (t, J = 6.2 Hz, 2H), 3.22 (t, J = 6.5 Hz, 2H), 2.98 (t, J = 8.0 Hz, 2H), 2.50 (t, J = 8.0 Hz, 2H), 2.42 (s, 3H), 2.32 (s, 3H), 1.91–1.79 (m, 2H), 1.76–1.64 (m, 2H), 1.47 (s, 9H), 1.22 (t, J = 7.1 Hz, 3H) ; ^{13}C NMR (75 MHz, $CDCl_3$) δ ppm: 173.0, 158.3, 156.1, 141.8, 140.5, 140.2, 139.5, 137.7, 135.2, 134.5, 131.4, 131.4, 130.2, 129.7, 128.1, 127.6, 126.8, 126.4, 115.2, 112.2, 79.3, 67.6, 60.5, 40.5, 35.6, 28.7, 28.6 (3x), 27.0, 26.7, 21.6, 20.7, 14.3.

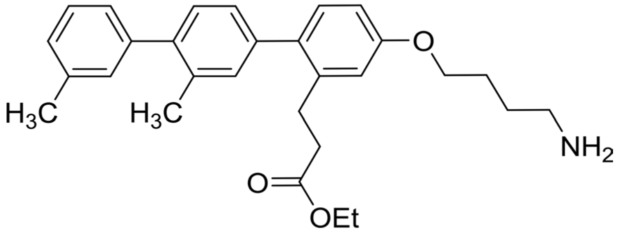

Production of: ethyl 3-(4-(4-aminobutoxy)-3′,3″-dimethyl-[1,1′:4′,1″-terphenyl]-2-yl)propanoate (compound 2). Compound **1** (1.10 g, 2.01 mmol) was dissolved in 40 mL of dichloromethane (DCM) under argon atmosphere and cooled with an ice bath. Next, trifluoroacetic acid (2.3 mL, 15 equiv.) was added dropwise to the reaction mixture. The reaction was stirred at room temperature until completion (24 h). Then, the mixture was neutralized with 1 M Na_2CO_3 solution and extracted with DCM (3 × 30 mL). The combined organic layers were dried over Na_2SO_4, filtered and concentrated. The crude was purified by flash chromatography on silica gel (DCM/ methanol, 5:1) to afford 868 mg of compound **2** as a colorless oil. Yield 97%. ^{1}H NMR (300 MHz, $CDCl_3$) δ ppm: 7.36-7.29 (m, 1H), 7.28–7.10 (m, 7H), 6.85 (d, J = 2.7 Hz, 1H), 6.80 (dd, J_1 = 8.4 Hz, J_2 = 2.7 Hz, 1H), 4.09 (q, J = 7.2 Hz, 2H), 4.05 (t, J = 5.6 Hz, 2H), 3.49 (q, J = 6.3 Hz, 2H), 2.98 (t, J = 7.8 Hz, 2H), 2.49 (t, J = 8.0 Hz, 2H), 2.42 (s, 3H), 2.32 (s, 3H), 1.93–1.78 (m, 4H), 1.21 (t, J = 7.1 Hz, 3H); ^{13}C NMR (75 MHz, $CDCl_3$) δ ppm: 173.1, 158.1, 141.8, 140.6, 140.1, 139.6, 137.8, 135.2, 134.9, 131.5, 131.4, 130.2, 129.7, 128.1, 127.6, 126.8, 126.5, 115.2, 112.2, 67.4, 60.5, 39.8, 35.6, 28.7, 26.5, 26.1, 21.6, 20.7, 14.3.

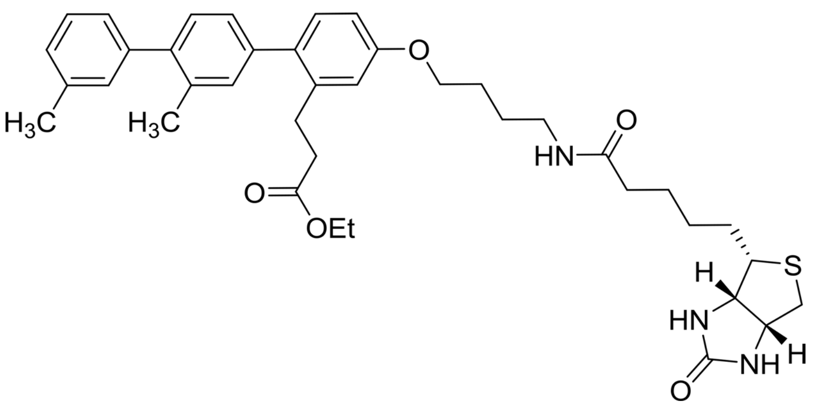

Production of: ethyl 3-(3′,3′′-dimethyl-4-(4-(5-((3aS,4S,6aR)-2-oxohexahydro-1H-thieno[3,4-d]imidazol-4-yl) pentanamido)butoxy)-[1,1′:4′,1′′-terphenyl]-2-yl)propanoate (compound 3). Compound **2** (868 mg, 1.95 mmol) and biotin (476 mg, 1.95 mmol) were dissolved in 20 mL of anhydrous DMF and the mixture was cooled in an ice bath. Next, 1-(3-dimethylaminopropyl)-3-ethylcarbodiimide methiodide (EDCMetI) (579 mg, 2.92 mmol), 1-hydroxybenzotriazole hydrate (HOBt) (395 mg, 2.92 mmol) and triethylamine (197 mg, 1.95 mmol) were added to the reaction mixture. The reaction was stirred at room temperature under argon atmosphere until completion (2 days). Next, the concentrated crude reaction was taken up in H_2O (10 mL) and extracted with DCM (3 × 20 mL). The combined organic layers were dried over Na_2SO_4, filtered and concentrated. The crude was purified by flash chromatography on silica gel (DCM/methanol, 20:1) to afford 774 mg of compound **3** as a white solid. Yield 59%, mp: 137–139° C. ^{1}H NMR (300 MHz, $CDCl_3$) δ ppm: 7.35–7.28 (m, 1H), 7.26–7.10 (m, 7H), 6.83 (d, J = 2.4 Hz, 1H), 6.79 (dd, J_1 = 8.4 Hz, J_2 = 2.4 Hz, 1H), 6.45 (s, 1H, NH), 6.22 (s, 1H, NH), 5.53 (s, 1H, NH), 4.54–4.45 (m, 1H), 4.35–4.27 (m, 1H), 4.08 (q, J = 7.1 Hz, 2H), 4.01 (t, J = 6.0 Hz, 2H), 3.33 (q, J = 6.6 Hz, 2H), 3.19–3.10 (m, 1H), 2.96 (t, J = 8.0 Hz, 2H), 2.90 (dd, J_1 = 12.9 Hz, J_2 = 4.8 Hz, 1H), 2.72 (d, J = 12.9 Hz, 1H), 2.48 (t, J = 8.0 Hz, 2H), 2.41 (s, 3H), 2.30 (s, 3H), 2.22 (t, J = 7.4 Hz, 2H), 1.91–1.78 (m, 2H), 1.77–1.62 (m, 6H), 1.52–1.40 (m, 2H), 1.20 (t, J = 7.2 Hz, 3H); ^{13}C NMR (75 MHz, $CDCl_3$) δ ppm: ^{13}C NMR 173.4, 173.1, 164.1, 158.3, 141.8, 140.6, 140.1, 139.5, 137.8, 135.2, 134.6, 131.5, 131.4, 130.2, 129.7, 128.1, 127.6, 126.8, 126.5, 115.2, 112.3, 67.7, 61.9, 60.5, 60.3, 55.8, 40.7, 39.3, 36.2, 35.7, 28.7, 28.3, 28.2, 26.9, 26.5, 25.9, 21.6, 20.7, 14.3.

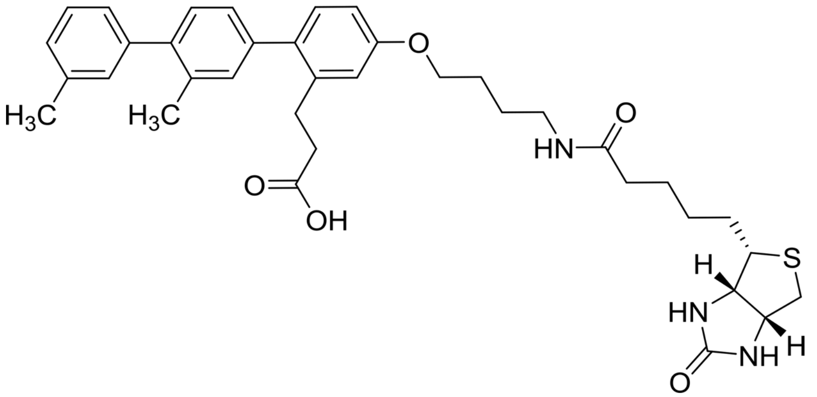

Production of: 3-(3′,3′′-dimethyl-4-(4-(5-((3aS,4S,6aR)-2-oxohexahydro-1H-thieno[3,4-d]imidazol-4-yl)pentanamido) butoxy)-[1,1′:4′,1′′-terphenyl]-2-yl)propanoic acid (bioT12). Compound **3** (700 mg, 1.04 mmol) was dissolved in 20 mL of a tetrahydrofuran (THF)/water mixture (4:1) and then lithium hydroxide (131 mg, 3.12 mmol) was added. The reaction was stirred at room temperature until completion. The mixture was neutralized by adding 1 M HCl solution and the THF was eliminated *in vacuo*. The aqueous layer was extracted with AcOEt (3 × 50 mL), the organic layers were dried over Na_2SO_4, filtered and concentrated. The crude was purified by crystallization from ethanol to yield 616 mg of **bioT12** as a white solid. Yield 92%. ^{1}H NMR (300 MHz, MeOD + $CDCl_3$) δ ppm: 7.31–7.24 (m, 1H), 7.20–7.06 (m, 7H), 6.86 (d, J = 2.4 Hz, 1H), 6.78 (dd, J_1 = 8.4 Hz, J_2 = 2.4 Hz, 1H), 4.49–4.41 (m, 1H), 4.31–4.24 (m, 1H), 4.01 (t, J = 6.2 Hz, 2H), 3.26 (t, J = 6.8 Hz, 2H), 3.21–3.13 (m, 1H), 2.91 (t, J = 7.8 Hz, 2H), 2.90–2.83 (m, 1H), 2.69 (d, J = 12.9 Hz, 1H), 2.43 (t, J = 8.0 Hz, 2H), 2.37 (s, 3H), 2.25 (s, 3H), 2.22 (t, J = 7.2 Hz, 2H), 1.89–1.76 (m, 2H), 1.76–1.54 (m, 6H), 1.49–1.37 (m, 2H); ^{13}C NMR (75 MHz, MeOD + $CDCl_3$) δ ppm: 177.3, 175.9, 165.8, 159.5, 142.8, 141.6, 141.4, 140.6, 138.6, 136.0, 135.5, 132.2, 132.1, 130.8, 130.4, 128.9, 128.4, 127.7, 127.2, 116.0, 113.1, 68.4, 63.1, 61.4, 56.8, 41.0, 40.0, 36.7, 36.6, 29.7, 29.6, 29.3, 27.7, 27.0, 26.8, 21.7, 20.8.

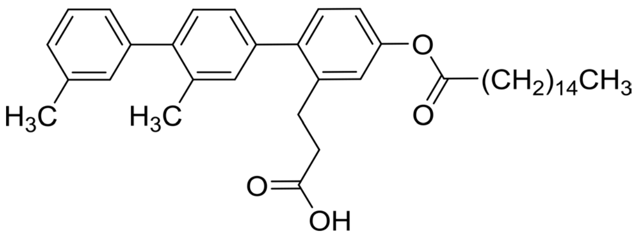

Production of: 3-(3′,3′′-dimethyl-4-(palmitoyloxy)-[1,1′:4′,1′′-terphenyl]-2-yl)propanoic acid (T43). To a solution of compound **12a** (50 mg, 0.14 mmol) and triethylamine (29 mg, 0.30 mmol) in 10 mL of dry DCM was added palmitoyl chloride (43 mg, 0.16 mmol) and the reaction was stirred overnight at room temperature. The mixture was acidified by adding 1 M HCl solution and the layers were separated. The aqueous layer was extracted with DCM (3 × 10 mL), the organic layers were dried over Na_2SO_4, filtered and concentrated. The crude was purified by flash chromatography (hexane/AcOEt, 3:1) to yield 13 mg of **T43** as a colorless oil. Yield 15%. ^{1}H NMR (300 MHz, $CDCl_3$) δ ppm: 7.35–7.28 (m, 1H), 7.28–7.11 (m, 7H), 7.02 (d, J = 2.1 Hz, 1H), 6.99 (dd, J_1 = 8.1 Hz, J_2 = 2.4 Hz, 1H), 2.99 (t, J = 8.0 Hz, 2H), 2.62–2.49 (m, 4H), 2.41 (s, 3H), 2.31 (s, 3H), 1.83–1.71 (m, 2H), 1.40–1.21 (m, 24H), 0.88 (t, J = 6.8 Hz, 3H); ^{13}C NMR (75 MHz $CDCl_3$) δ ppm: 177.6, 172.6, 150.2, 141.7, 141.1, 139.5, 139.2, 137.8, 135.4, 131.5, 131.2, 130.2, 129.8, 128.1, 127.7, 126.6, 126.5, 122.0, 119.6, 34.8, 34.6, 32.1, 29.8, 29.8, 29.6, 29.5, 29.4, 29.3, 28.2, 25.1, 22.8, 21.6, 20.7, 14.3.

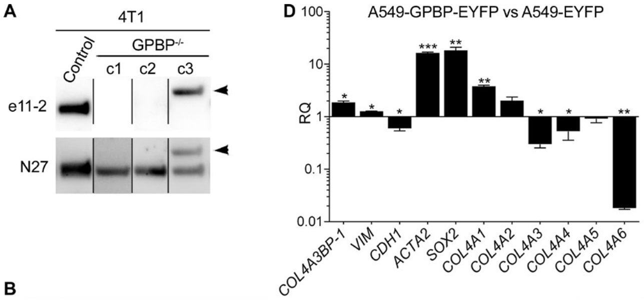

B

Clone	No. clones/sequences analyzed	No. alleles identified	Mutations found in exon 11 (target sequence)	Predicted translation (* = stop)	Frequency
1	14	4	TA deletion: CCCTA--GTCGCTCTTCCTCCA	P*	6
			A insertion: CCCTATAAGTCGCTCTTCCTCCA	PYKSLFLHVFH*	5
			AGTC deletion: CCCTAT----GCTCTTCCTCCA	PYALPPCLPL*	2
			G deletion: CCCTATA-TCGCTCTTCCTCCA	PYIALPPCLPLI*	1
2	15	3	A insertion: CCCTATAAGTCGCTCTTCCTCCA	PYKSLFLHVFH*	11
			TA deletion: CCCTA--GTCGCTCTTCCTCCA	P*	2
			G deletion: CCCTATA-TCGCTCTTCCTCCA	PYIALPPCLPLI*	2
3	15	3	A insertion: CCCTATAAGTCGCTCTTCCTCCA	PYKSLFLHVFH*	8
			GT deletion: CCCTATA--CGCTCTTCCTCCA	PYTLFLHVFH*	6
			T insertion: CCCTATTAGTCGCTCTTCCTCCA	PY*	1

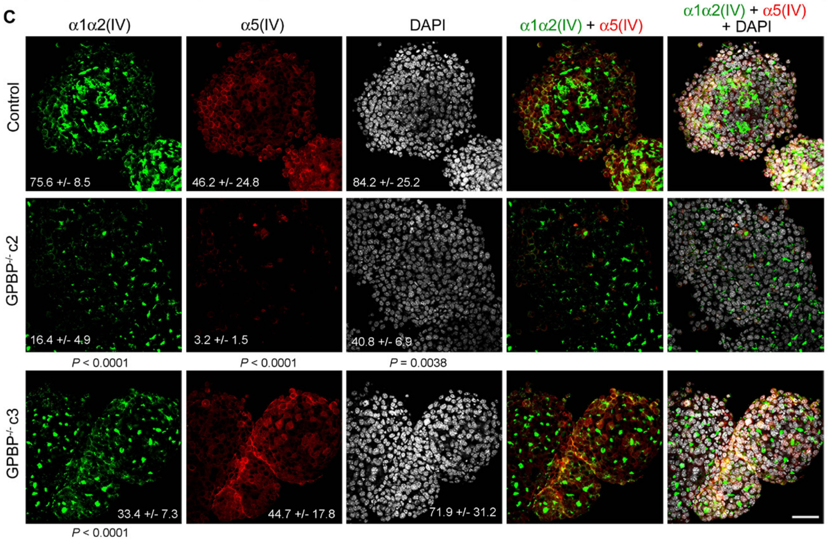

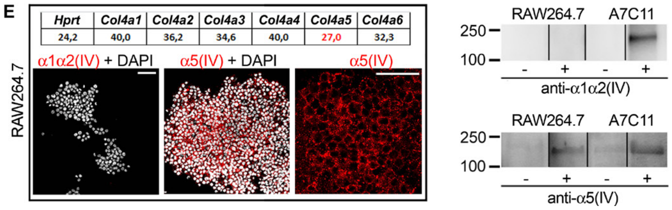

Supplementary Figure 1: (**A**) Selection of 4T1 GPBP$^{-/-}$ clones obtained by CRISPR/Cas9 genome editing. 4T1 cells were co-transfected with individual plasmids expressing Cas9 and a guide RNA (gRNA) that hybridizes with 5′-CCCTATAGTCGCTCTTCCTCCA-3′ of exon 11, exclusive of GPBP mRNA. The efficiency of the procedure was assessed by *Cel*I digestion of a genomic PCR product encompassing the target sequence. Clones were selected by limiting dilution. A sample of each clone was lysed and the presence of GPBP (e11-2) or GPBP and GPBP-2/CERT (N27) assessed by WB. Clone 3 expressed an abnormal sized GPBP (arrowheads). Primary 4T1 cells were also analyzed (Control). (**B**) Genotypic characterization of 4T1 GPBP$^{-/-}$ clones. The genomic PCR products encompassing the target sequence from each clone were ligated into an appropriate plasmid, and clones of the corresponding alleles were isolated and nucleotide sequenced. Mutations found in clone 3 did not account for the abnormal GPBP and thus the origin of this GPBP isoform remains unknown. (**C**) Phenotypic characterization of 4T1 GPBP$^{-/-}$clones. The clones indicated were cultured in 3D and spheroids analyzed by IF-CM for collagen IV expression (c1 is displayed in Figure 1C). Fluorescence intensities were measured and expressed as in Figure 1. Statistics: 1WA-D respect to Control. (**D**) Individual A549 clones expressing GPBP-EYFP (clone B11) exhibited mesenchymal progenitor mixed phenotype. RQ of the indicated mRNA is represented as in Figure 1. Statistics: Student's *t*-test, $n = 2$. (**E**) RAW 264.7 murine macrophages express mainly *COL4A5***.** *Left*, the indicated 2D (Table) and 3D (IF-CM) cultures were analyzed as in Figures 1A and 1C, respectively. *Right*, the indicated 2D cultures were extracted and undigested collagen IV chains analyzed by WB with the indicated antibodies. Numbers and bars denote size (kDa) and position of the MW markers. Unless otherwise indicated scale bars are 50 µm.

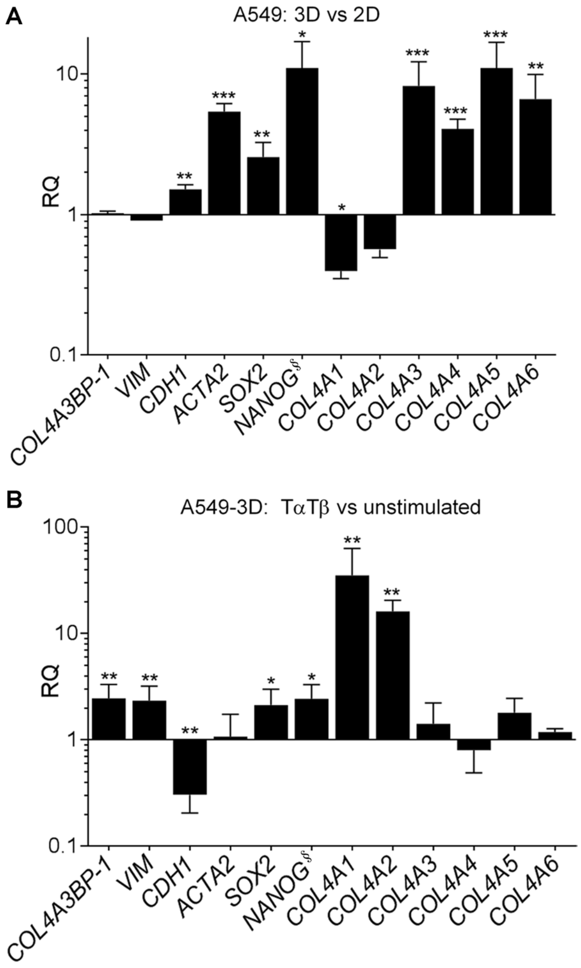

Supplementary Figure 2: (**A**) A549 spheroid formation (**B**) TαTβ spheroid stimulation. Bars represent relative quantity (RQ) of indicated mRNA expressed as mean ± SD for the indicated comparisons. Statistics: Student's *t*-test, $n = 4$. [§]NANOG expression levels were very low in 2D cultures and the expression in 3D cultures was still low in absolute values.

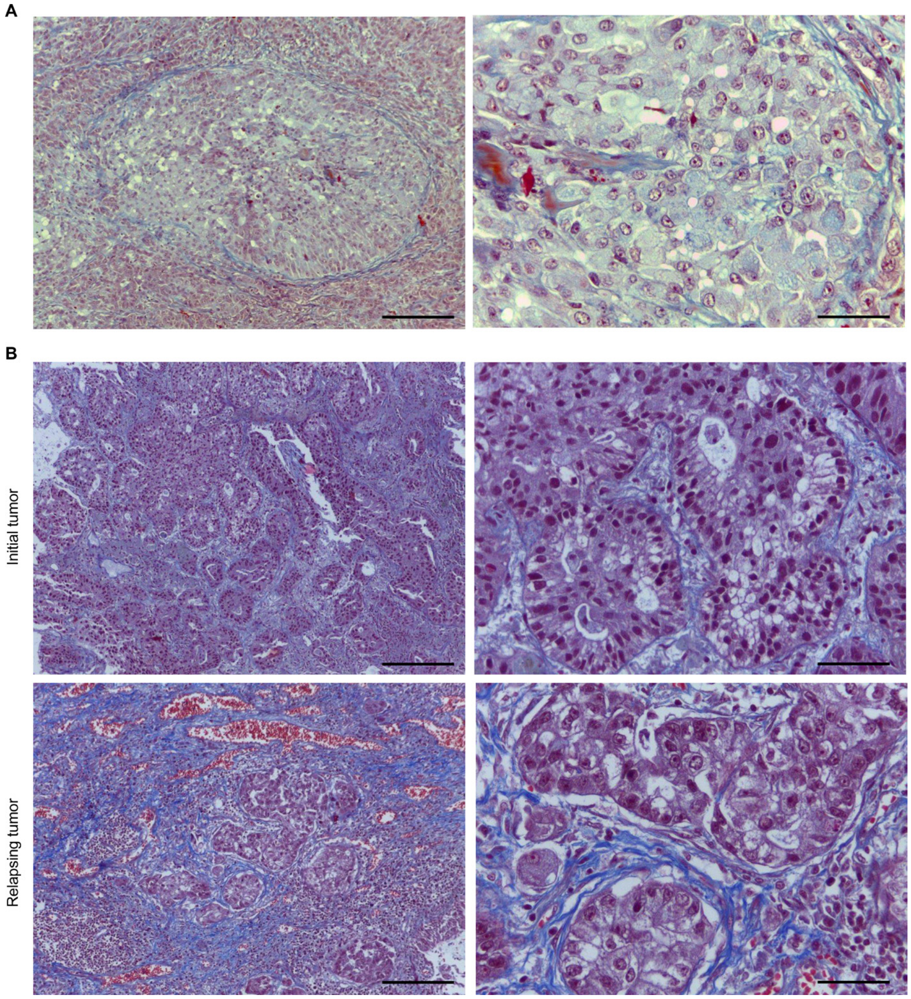

Supplementary Figure 3: (**A**) Masson staining of human small A549 xenografts. Aniline blue dye stained collagenous material in the stroma but also in mesenchymalized epithelial cells occupying tumor nodular regions (*left*, bars = 200 µm). (**B**) Masson staining of human NSCLC at diagnosis (Initial tumor) and at relapsing after chemotherapy (Relapsing tumor). At diagnosis, the primary tumor exhibited a characteristic histology of adenocarcinoma with typical acinar structures exhibiting epithelial organization and limited collagenous material in the surrounding tumor stroma. In the relapsing tumor, there was loss of epithelial organization and abundant collagenous material in stroma and nodular regions (*left*, bars = 200 µm).

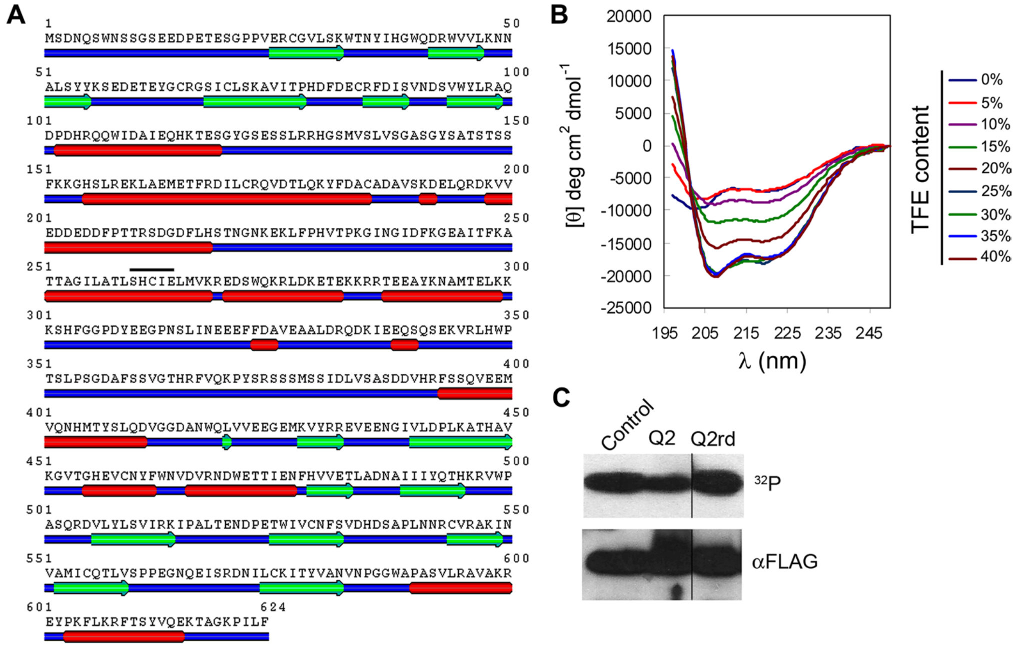

Supplementary Figure 4: (**A**) Predicted secondary structure of human GPBP. Shown is the primary structure of GPBP in one-letter code. Denoted are predicted β-sheet (green) and α-helix (red) segments, using the SABLE software from Division of Biomedical Informatics, Cincinnati Children's Hospital Research Foundation. The SHCIE motif (black line) maps within an α-helix context. (**B**) SHCIE is predicted to exhibit α-helix secondary structure. Q2 (CH_3CO-LATLSHCIELMVKR-NH_2), a peptide representing SHCIE and flanking sequences was analyzed by circular dichroism at the indicated trifluoroethanol (TFE) concentrations. Plotted are TFE titrations of Q2 solution revealing promptness of Q2 to form α-helix [43], thus supporting predictions in A. (**C**) Q2 inhibits GPBP autophosphorylation. Recombinant human GPBP produced in yeast was used for *in vitro* phosphorylation in the presence of Q2 or a randomized peptide with the same amino acid composition (Q2rd). Reactions were analyzed by SDS-PAGE, WB and autoradiography (^{32}P). Finally, the membrane was probed with anti-FLAG. (B, C). Shown is a representative assay of two.

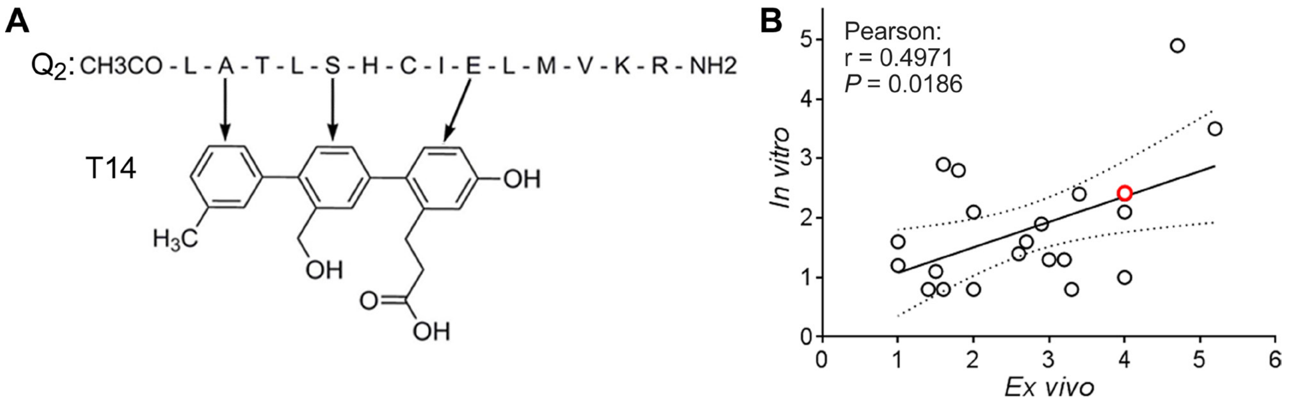

Supplementary Figure 5: (**A**) Design of a peptidomimetic of Q2. The correspondence among residue lateral chains (Q2) and chemical group substituents (T14) are denoted by arrows. (**B**) *In vivo* and *ex vivo* activities of terphenyls are related variables. Plotted are GPBP autophosphorylation (*in vitro*) and A549 doxorubicin IC50 (*ex vivo)* at 50 μM of individual T14-related terphenyls (circles) expressed as relative value respect to untreated (see Supplementary Table 1). T12: red circle; Statistics: Pearson test.

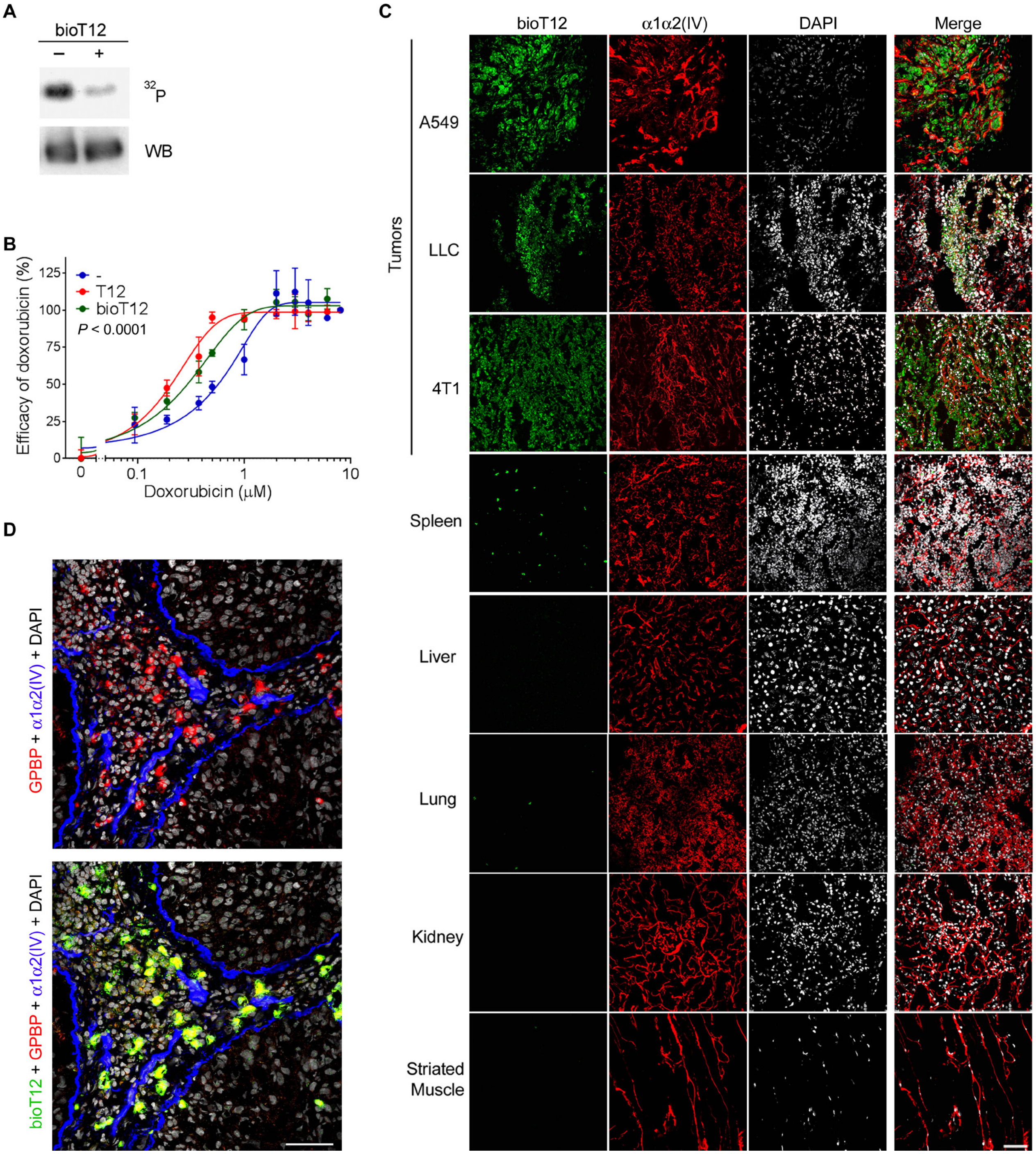

Supplementary Figure 6: (**A**) BioT12 inhibits GPBP autophosphorylation *in vitro*. FLAG-GPBP was assayed for autophosphorylation with (+) or without (–) 50 μM of bioT12 and analyzed essentially as in Figure 5A. (**B**) BioT12 reduces doxorubicin IC50 *ex vivo*. Cells were seeded in 96-well plates and incubated for 36 h with increasing concentrations of doxorubicin and 50 μM of the indicated compound. Cell viability was analyzed and represented as in Figure 5A. Shown are means ± SD of a representative assay done in quadruplicate. Similar results were obtained in an additional assay. Statistics: 2WA-D comparing T12 or bioT12 curves with control. (**C**) BioT12 binds to tumors but not to normal tissues. Frozen sections of the indicated tumors and C57BL/6 mouse organs were IF-CM analyzed using the indicated reagents and with antibodies against the polypeptides also indicated (bar = 100 μm). Shown are selected representative images. Similar conclusions were obtained when staining organs from BALB/c. (**D**) BioT12 binds to a limited number of cancer cells overexpressing GPBP in naturally occurring human NSCLC. Frozen sections of patient adenocarcinoma were IF-CM analyzed to visualize *in situ* T12 binding (bioT12) and the indicated polypeptides in a representative tumor region.

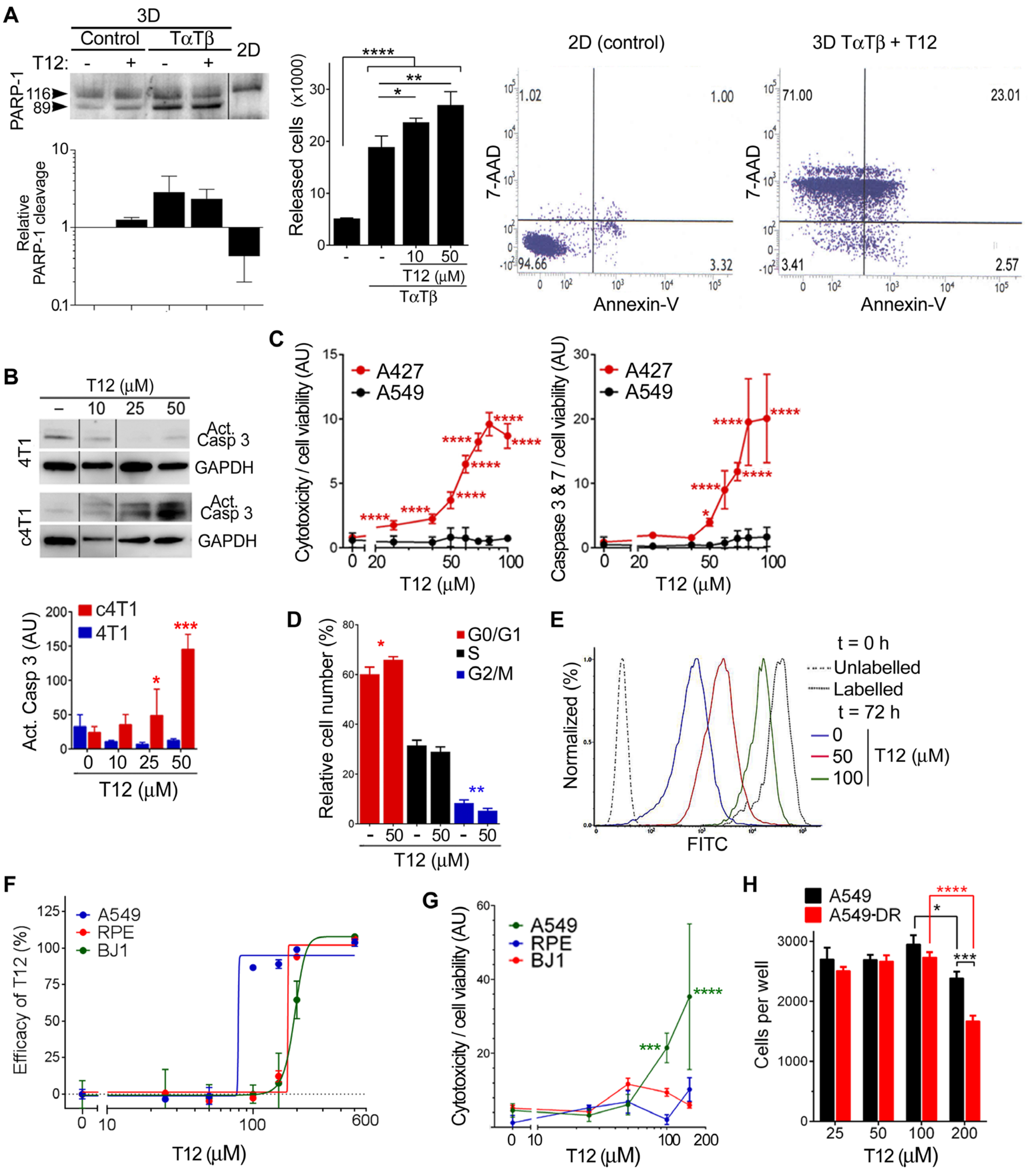

Supplementary Figure 7: (**A**) T12 induces cell detachment from EMT A549 spheroids. *Left*, A549 spheroids were cultured, stimulated (day-1 to day-4) and treated with 50 μM T12 (day-3 to day-4). Then cells were lysed and analyzed by WB to assess PARP-1 cleavage. Plotted is cleaveage of PARP-1 [89-kDa/(89-kDa+116-kDa)] respect to untreated control (mean ± SD) which was set at 1, $n = 2$. *Middle*, bars indicate the number of cells found in the culture media of A549 spheroids untreated (–) or stimulated and treated as at the left. TαTβ induced and T12 enhanced cell detachment. The number of cells in culture media was determined using a MOXI Z Mini Automated Cell Counter. Shown are mean ± SD of a representative assay done in duplicate. Statistics (Left and Middle): 1WA-T. Similar results were obtained in two additional assays. *Right*, the indicated detached cells were collected and analyzed by flow cytometry using PE Annexin V Apoptosis Detection Kit (BD Bioscience) to determine live cells (lower left area), apoptosis (lower right area), late apoptosis (upper right area) or necrosis (upper left area). Indicated are the percentages of cells in each area. Similar results were obtained in two independent assays. Cells detached from TαTβ-stimulated spheroids exhibited similar distribution whether untreated or treated with 10 or 50 (shown) μM T12. Where indicated lysed or trypsinized A549 2D cultures were used as internal control. (**B**) T12 activates caspase 3 in cultures of c4T1 in a dose-dependent manner. Caspase 3 activation was analyzed by WB (image) for the indicated cells and treatments (72 h). Values

were normalized with GAPDH (mean ± SD) and plotted (histogram). Statistics: 2WA-F, $n = 2$. (**C**) T12 induces more cytotoxicity and apoptosis in "mesenchymal" A427 than in "epithelial" A549. Plotted are the indicated variables (mean ± SD) measured using ApoTox-Glo-Triplex Assay (Promega) in the cultures and treatments (40 h) which are also indicated. Statistics: 2WA-S, $n = 4$. Representative of two assays. (**D**) T12 induces "epithelial" cancer cells to accumulate in G0/G1. The bars indicate the cell cycle phase distribution of untreated A549 cultures (−) or treated (48 h) with the indicated concentration of T12. Bars represent mean ± SD of two independent assays done in duplicate. Statistics: Student's *t*-test. (**E**) T12 impairs "epithelial" cancer cell proliferation. A549 cell proliferation was monitored using CellTrace™ Cell Proliferation Kit (Thermo Fisher Scientific). The cell dye load drops by half in each division. Represented are normalized cell dye load distributions setting at 1 the mode value of each individual treatment. FITC stands for FI indicative of dye load. (**F**) Immortalized non-malignant cells are less sensitive to T12 than cancer cells. Represented are viability assays for A549 and for telomerase-immortalized hTERT-RPE-1 (RPE) and hTERT-BJ1 (BJ1) cultures (Clontech), representing human retina (epithelial) and fibroblast (mesenchymal), respectively. Cell viability was assessed and represented as in Figure 5A. Shown are mean ± SD of a representative assay done in quadruplicate. Similar results were obtained in three independent assays. Maximum efficacy on A549 cultures was set at 100%. (**G**) T12 exhibited reduced cytotoxicity for cell cultures representing immortalized non-malignant cells. The cultures in (**F**) were subjected to the indicated treatments for 40 h and the indicated variable (mean ± SD) measured using CytoTox-Glo™ Cytotoxicity Assay (Promega) and plotted. Statistics: 2WA-D ($n = 4$, comparison respect T12 = 0 µM in each series). Similar results were obtained in three independent assays done in triplicate. (**H**) Cancer epithelial cells exhibiting drug-resistance were more responsive to T12. The indicated cell cultures were subjected to the indicated treatments (48 h) and cells per well were measured using alamarBlue® and a standard run in parallel of the corresponding cell type. Statistics: 2WA-S ($n = 2$).

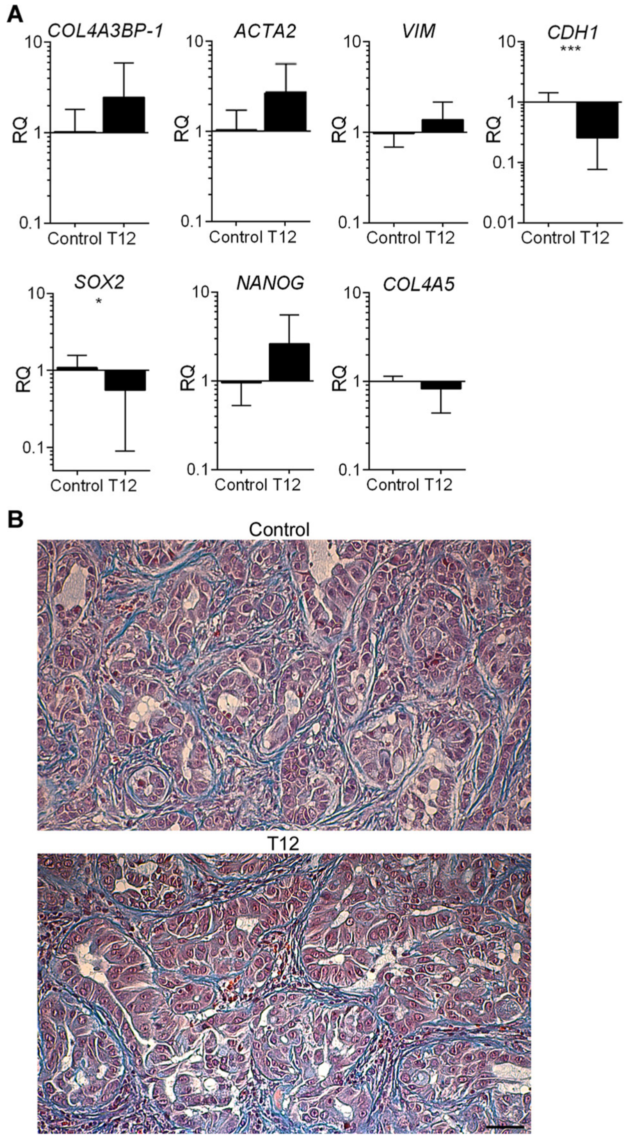

Supplementary Figure 8: T12 does not disrupt epithelial A549 tumor organization. (**A**) RQ (mean ± SD) of the indicated mRNA in T12-treated large R-A549 tumors vs untreated (Control). Statistics: Student´s *t*-test; *upper row*, Control, $n = 4$, T12, $n = 5$; *lower row*, Control, $n = 8$, T12, $n = 10$. (**B**) Masson's trichrome staining of specimens representing untreated (Control) and treated (T12) large R-A549 tumors. Shown are selected representative images.

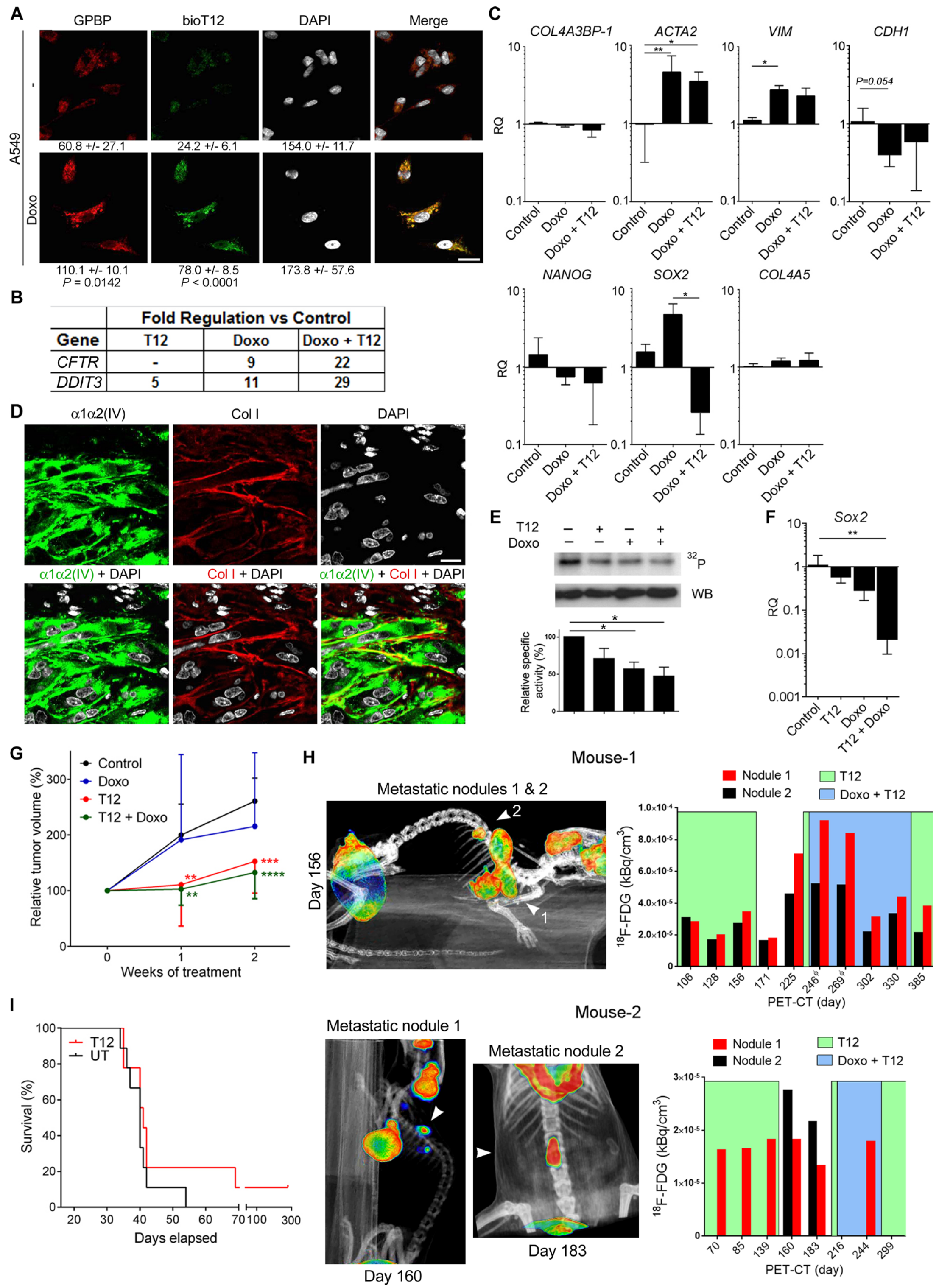

Supplementary Figure 9: (**A**) Doxorubicin induces A549 cells to express mesenchymal GPBP. A549 cultures were treated with 1 µM doxorubicin for 24 h, fixed and analyzed by IF-CM using the indicated reagents and N27 to visualize GPBP (bar = 20 µm). Indicated are FI measured, expressed and compared as in Fig.1 Statistics: Student's *t*-test. (**B**) T12 enhances doxorubicin-induced expression of *DDIT3* and *CFTR*. The table shows the relative mRNA levels determined in an expression array of A549 cells treated with 50 µM T12 and/or 1 µM doxorubicin for 36 h with respect to untreated cells (Control). (**C**) T12 specifically targets progenitor cells in large A549 tumors sensitized with doxorubicin. RQ (mean ± SD) of the indicated mRNA in large A549 tumors from mice treated with doxorubicin (Doxo) or with doxorubicin and T12 (Doxo+T12) vs untreated (Control). Statistics: 2WA-T, n = 8 (Control), n = 9 (Doxo) and n = 10 (Doxo + T12). (**D**) Doxorubicin induces collagen IV intracellular accumulation. Detail of the large A549 tumor treated with doxorubicin analyzed by IF-CM in Fig 9F (bar = 10 µm). Shown are selected representative images. (**E**) Doxorubicin-treated A549 cells secrete doxorubicin- and

T12-sensitive GPBP species. A549 cells were incubated with 0.5 µM doxorubicin in serum-free medium containing 0.4% BSA during 6 h. The medium (50 mL) was collected and extracted with agarose-scFvN26. Bound GPBP was eluted with PS132 peptide in TBS and used in phosphorylation assays with 1 µM doxorubicin and 50 µM T12 where indicated (+). Reactions were analyzed by SDS-PAGE, blotted, autoradiographed (^{32}P) and developed with anti-GPBP mAb e26 (WB). Specific activities (mean ± SEM) relative to control reaction (100%) were calculated with WCIF ImageJ software and represented (GraphPad Prism). Statistics: 1WA-F, n = 2. (**F**) T12 specifically targets progenitor cells in 4T1 tumors sensitized with doxorubicin. RQ (mean ± SD) of *Sox2* in 4T1 tumors from the assay showed in Figure 9H. Statistics: 1WA-T, n = 10. (**G**) Doxorubicin does not enhance T12 efficacy over small A549 tumors. Represented is the indicated variable (mean ± 95% CI) over time in A549 mouse-model bearing small A549 tumors treated with the indicated compounds. Statistics: 2WA-S, n = 14. (**H**) PET-CT analysis of mouse-1. Indicated (arrowheads) are the emission of radiation by non-physiologic uptake of ^{18}F-FDG (metastatic nodules) and the day of the analysis. Other PET signals correspond to physiological activity (central nervous system, heart and bladder) or accumulation in the injection site close to the bladder. Graphs, ^{18}F-FDG emission over time of each nodule. Periods and treatments are indicated with colors. $^{\Phi}$ Additional nodules were observed. (**I**) Mouse-2 production and PET-CT monitoring. Kaplan–Meier survival curves of 4T1 mouse-model treated with T12 upon primary tumor removal which rendered a second animal (mouse-2) with metastases that survived for more than one year with T12 treatment. Statistics: Log-rank (Mantel-Cox) test, P = 0.26. Mouse-2 was monitored by PET-CT analysis and data represented as in (H).

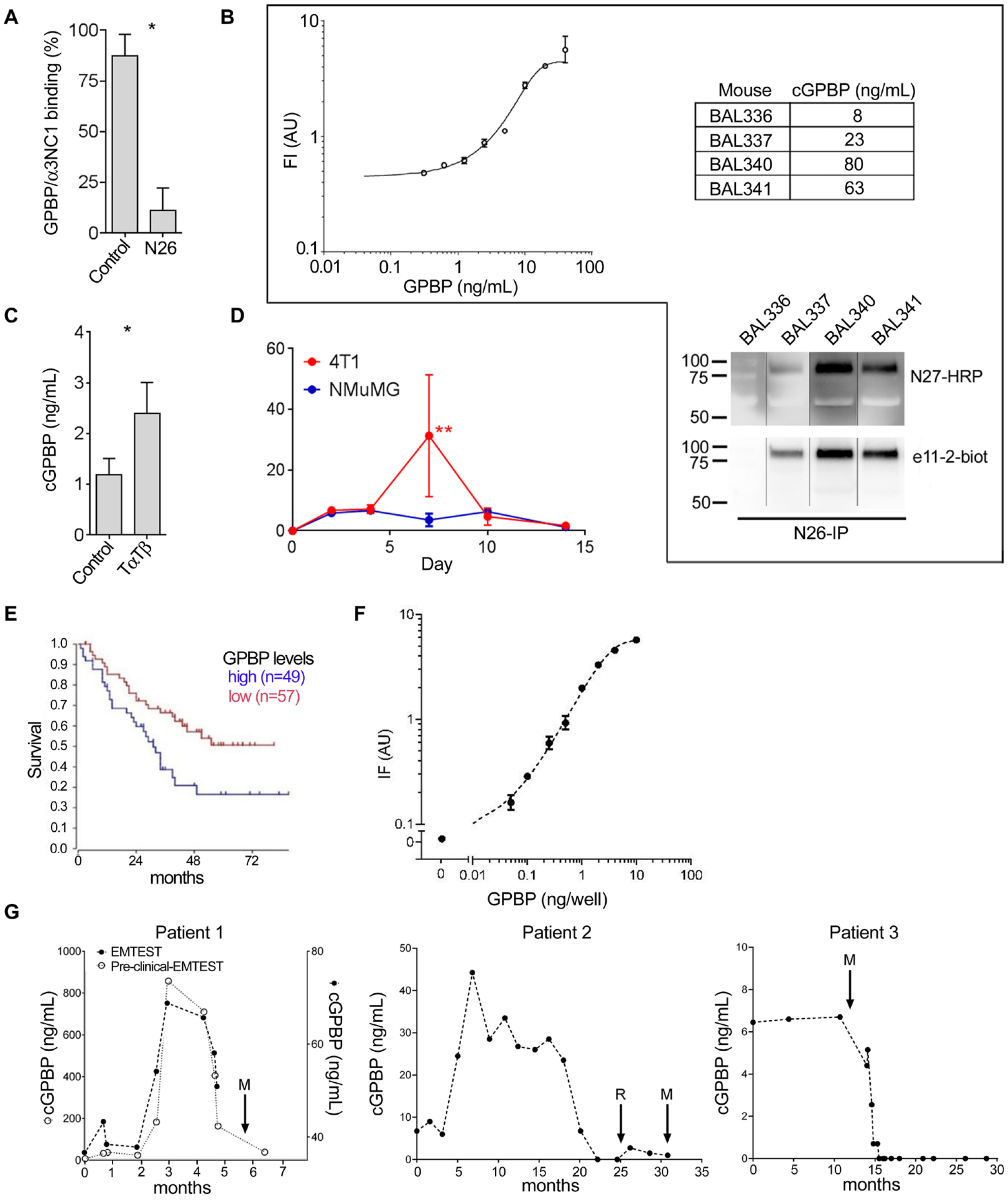

Supplementary Figure 10: (**A**) N26 antibody impairs GPBP interaction with collagen IV. Represented is the mean ± SD of the indicated molecular interaction in the absence (Control) or presence of the indicated mAb (N26). Control binding activity was set at 100%. Briefly, 96-well plates were coated with poly-His-GPBP in PBS. After blocking, plates were incubated with FLAG-α3NC1 in the absence (control) or presence of N26. Bound FLAG-α3NC1 was detected with anti-FLAG M2-HRP. All polypeptides were used at 1 μg/mL. Statistics: Student's *t*-test, $n = 2$. We tested 11 independent GPBP-specific mAb and selected N26 for its best performance *in vitro* and *in vivo*.[3] (**B**) Preclinical EMTEST is a reliable ELISA to measure cGPBP in mice. Shown is a log-log graph with the means ± SD and the best-fit curve obtained measuring the indicated amounts (dots) of calibrator (poly-His-GPBP) using preclinical EMTEST. *Table*, cGPBP levels calculated with preclinical EMTEST for the indicated individual 4T1 mouse-model plasma samples. *Image*, these plasma samples were immunoprecipitated with sepharose-N26 (the capturing antibody) and immunoprecipitated materials analyzed by WB with N27-HRP (the detecting antibody) or biotin-labeled e11-2 (e11-2-biot). (**C**) TαTβ induces the secretion of cGPBP. Bars represent cGPBP levels (mean ± SD) estimated with preclinical EMTEST in the culture media of A549 cells unstimulated (Control) or stimulated with TαTβ. Statistics: Student's *t*-test, $n = 4$. (**D**) Non-cancer NuMuG cells do not induce cGPBP levels in mice. cGPBP levels (mean ± SEM) over time were measured with preclinical EMTEST in plasma of BALB/c mice inoculated with the indicated cells (10^4). Statistics: 2WA-S, $n = 4$ per day. (**E**) *COL4A3BP* expression correlates with reduced overall survival in lung cancers. Kaplan-Meier plot of patients with lung cancer sorted depending on *COL4A3BP* expression (mRNA) in tumors. A cohort of 106 human lung cancer patients was used in the study. One way analysis of variance (ANOVA, $P = 0.011$). Total RNA was isolated from lung tumors and used to hybridize Affymetrix arrays (GeneChip Human Genome u133p2 array). Data obtained from Bild study at R2: microarray analysis and visualization platform (http://r2.amc.nl). (**F**) EMTEST calibrator for human plasma samples. A representative calibrator curve is shown using human plasma for analytical purposes and scFvN26 and FabN27 as capturing and detecting antibody, respectively. The dynamic range is from 0.1 ng to 10 ng. In this format, the recovery is > 95% using patient plasma samples. By measuring the same samples over three months the reproducibility was constant. The reagents can be stored during several months and the samples repeatedly analysed after several years of storage at –80° C with satisfactory reproducibility. (**G**) EMTEST monitoring of NSCLC patients. The EMTEST in F was used to measure cGPBP levels in individual serum (Patient 1) or plasma (Patients 2 and 3) samples (dots) collected at the indicated time points and stored at –80° C until analysis. For patient 1, cGPBP levels were assessed on fresh serum samples using preclinical EMTEST along with cGPBP levels of the same serum samples measured with EMTEST adapted for human plasma samples after several years and freezing-melting rounds. The different values obtained by preclinical and clinical EMTEST in Patient 1 is presently under investigation although the evidence supports the existence of GPBP-associated rheumatoid-like factors that enhance detection with preclinical EMTEST. Arrows indicate diagnosis of tumor relapsing (R), and metastases (M).

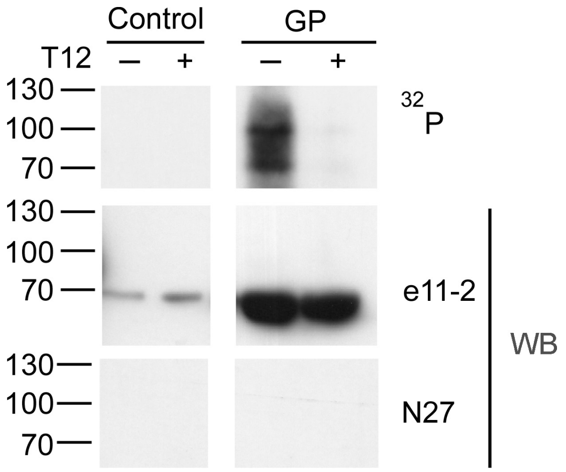

Supplementary Figure 11: T12 inhibits cGPBP from Goodpasture patients. Plasma samples from a Goodpasture patient (GP) and a healthy donor (Control) were purified and analyzed as in Figure 11F**.**

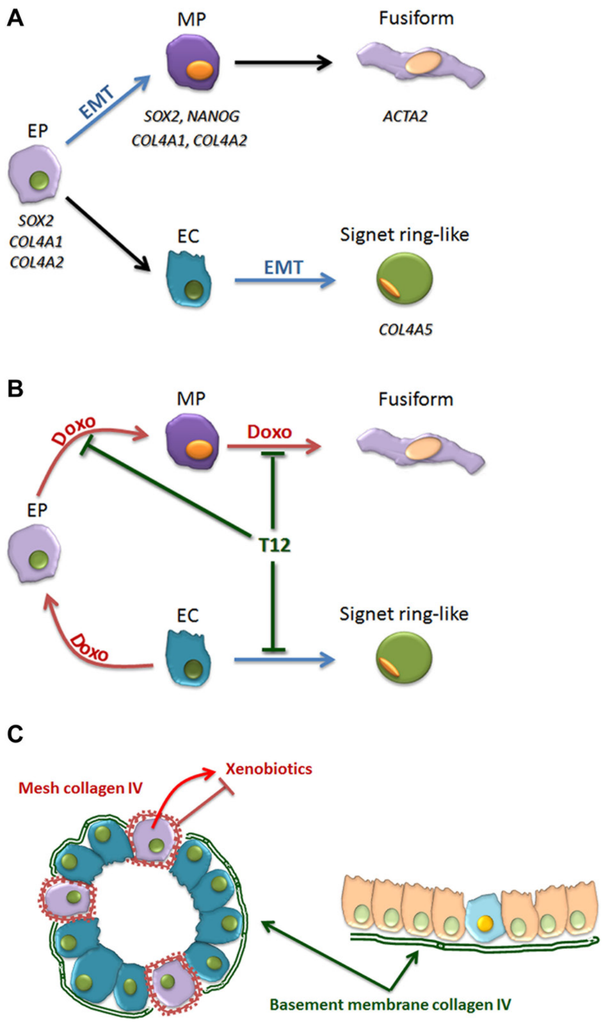

Supplementary Figure 12: Proposed model for T12 antitumor mechanism of action. (A) Cellular diversity in A549 tumors. A549 2D cultures contain high levels of E-cadherin and low levels of vimentin, representative of its epithelial phenotype and origin. A549 2D cultures contain an abundant SP along with non-SP. When 2D cultures are inoculated into nude mice, these populations became EP (epithelial progenitor) and EC (epithelial cell) in the tumor, respectively. In EMT, EP cells switch to mesenchymal progenitor cells (MP) that further differentiate to fusiform cells (tumor stroma) and EC cells switch to signet ring-like cells (tumor nodular regions). Mesh α1α2(IV) maintains MP and fusiform cells and mesh α5(IV) maintains signet ring-like cells. The evidence also suggest that MP cells express more *NANOG* than EP cells. *SOX2* and *NANOG* expression is reduced during mesenchymal differentiation to fusiform cells that express *ACTA2*. **(B)** T12 inhibits and doxorubicin induces EMT. T12 impairs mesh collagen IV formation required for EMT and mesenchymal progenitor differentiation, thus expanding EP cells. Doxorubicin induces EP cells expansion through “mesenchymalization” of EC [16], induces EMT to form MP and further mesenchymal progenitor differentiation, but not in the presence of T12 in which case EP cells further expand. **(C)** Mesh collagen IV surrounds EP cancer cells but not normal EP cells. Mesh collagen IV forms a barrier that impedes entry of xenobiotics into EP cancer cells and recruits CFTR for xenobiotic extrusion. Normal EP cells (light blue cell) are polarized by membrane collagen IV network and rely on ABC transporters for xenobiotic extrusion. By inhibiting mesh collagen IV formation, T12 renders cancer but not normal EP cells sensitive to xenobiotics.

Supplementary Table 1: Library of Q2 peptidomimetics. See Supplementary_Table_1

Supplementary Table 2: Mouse-1 and -2 follow-ups. See Supplementary_Table_2

NOTE

Auxiliary Supplementary Material is available upon request to the corresponding author.
