# Supplementary material for: Selective targeting of collagen IV in the cancer cell microenvironment reduces tumor burden
Source: Oncotarget. 2018 Jan 19;9(13):11020–45. doi: 10.18632/oncotarget.24280 (PMC5834258; doi:10.18632/oncotarget.24280)
Supplement: Supplementary file 2 [file oncotarget-09-11020-s002.docx]

**Supplementary Table 1: Library of Q2 peptidomimetics**

| **Terphenyl (ref. pat.)** | **Structure** | **MW** | **log P** | **H-bond donors** | **H-bond acceptors** | **Activity** | |
| --- | --- | --- | --- | --- | --- | --- | --- |
|  |  |  |  |  |  | ***ex vivo*** | ***in vitro*** |
| **1 (22e)** | 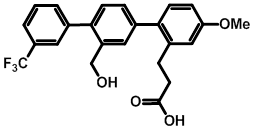 | 430.42 | 5.52 | 2 | 7 | 2.0 | 0.8 |
| **2 (22c)** | 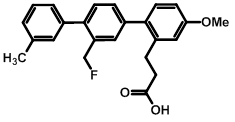 | 378.44 | 5.80 | 1 | 4 | 3.3 | 0.8 |
| **3 (22g)** | 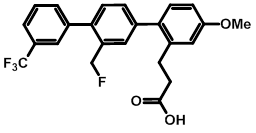 | 432.41 | 6.23 | 1 | 7 | 4.0 | 1 |
| **4 (22d)** | 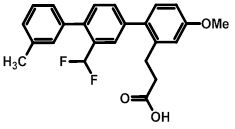 | 396.43 | 5.93 | 1 | 5 | 3.2 | 1.3 |
| **5 (22h)** | 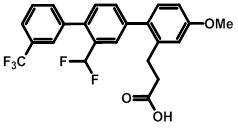 | 450.40 | 6.36 | 1 | 8 | 4.0 | 2.1 |
| **9 (12b)** | 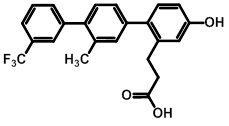 | 400.39 | 6.32 | 2 | 6 | 2.0 | 2.1 |
| **10 (1b)** | 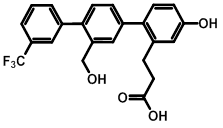 | 416.39 | 5.26 | 3 | 7 | 1.4 | 0.8 |
| **11 (22a)** |  | 376.44 | 5.09 | 2 | 4 | 1.6 | 0.8 |
| **12 (22b)** | 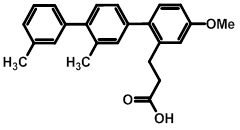 | 360.45 | 6.15 | 1 | 3 | 4.0 | 2.4 |
| **13 (12a)** | 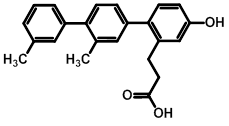 | 346.42 | 5.89 | 2 | 3 | 2.9 | 1.9 |
| **14 (1a)** |  | 362.42 | 4.83 | 3 | 4 | 1.6 | 2.9 |
| **32 (2a)** | 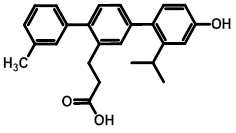 | 374.47 | 6.64 | 2 | 3 | 1.5 | 1.1 |
| **34 (15a)** | 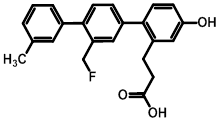 | 364.41 | 5.53 | 2 | 4 | 3.2 | 1.3 |
| **35 (20a)** | 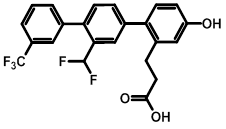 | 382.40 | 5.67 | 2 | 5 | 2.7 | 1.6 |
| **36 (20b)** | 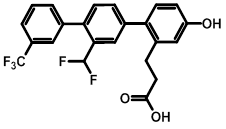 | 436.37 | 6.10 | 2 | 8 | 3.0 | 1.3 |
| **39 (34a)** | 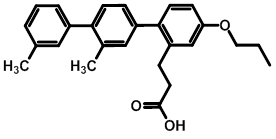 | 388.50 | 6.97 | 1 | 3 | 2.6 | 1.4 |
| **40 (24b)** | 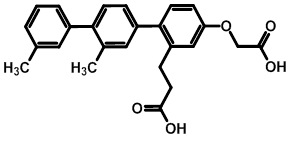 | 404.46 | 5.42 | 2 | 5 | 1.0 | 1.6 |
| **43** | 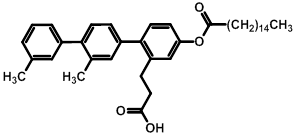 | 584.83 | 11.94 | 1 | 4 | 1.0 | 1.2 |
| **48 (22f)** | 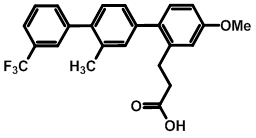 | 414.42 | 6.58 | 1 | 6 | 3.4 | 2.4 |
| **50 (32)** | 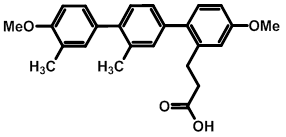 | 390.47 | 6.02 | 1 | 4 | 5.2 | 3.5 |
| **51 (24c)** | 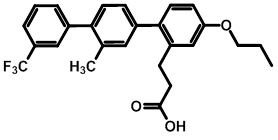 | 442.47 | 7.41 | 1 | 6 | 1.8 | 2.8 |
| **52 (15b)** | 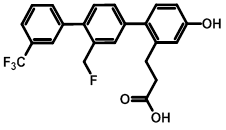 | 418.38 | 5.97 | 2 | 7 | 4.7 | 4.9 |

Columns (left to right): terphenyl number and patent reference between parenthesis; chemical structures determined by ^1^H and ^13^C NMR; MW; log P calculated using the Chemdraw Professional 15.0 algorithms; number of H-bond donors; number of H-bond acceptors; the *ex vivo* activity which was calculated by dividing the A549 doxorubicin IC50 in absence of peptidomimetic by that determined in the presence of the peptidomimetic (50 μM); *in vitro* activity which was calculated by dividing GPBP specific autokinase activity in absence of peptidomimetic by specific activity in the presence of peptidomimetic (50 μM).
