# Supplementary material for: Selective targeting of collagen IV in the cancer cell microenvironment reduces tumor burden
Source: Oncotarget. 2018 Jan 19;9(13):11020–45. doi: 10.18632/oncotarget.24280 (PMC5834258; doi:10.18632/oncotarget.24280)
Supplement: Supplementary file 3 [file oncotarget-09-11020-s003.docx]

**Supplementary Table 2: Mouse-1 and -2 follow-ups**

| **Mouse-1** | **Days after 4T1 inoculation** | **Observations** |
| --- | --- | --- |
| **Birth** | **-46** |  |
| **Inoculation of 4T1 cells** | **0** |  |
| **Mastectomy and**  **beginning of T12 treatment (0.1 mg/mL)** | **16** |  |
| **PET-CT (1)** | **106** | **Metastatic nodules detected** |
| **T12 dose raised (0.5 mg/mL)** | **106** |  |
| **PET-CT (2)** | **128** | **Reduced or maintained PET activity** |
| **PET-CT (3)** | **156** |  |
| **T12 treatment discontinued** | **158** |  |
| **PET-CT (4)** | **171** |  |
| **PET-CT (5)** | **225** | **Raise of PET activity** |
| **T12 treatment restarted (0.1 mg/mL)** | **226** |  |
| **Exophthalmos observed** | **239** | **Disease progression** |
| **Additional treatment with doxo (ip; 2 mg/kg)** | **241** |  |
| **PET-CT (6)** | **246** | **Additional nodules detected** |
| **Additional treatment with doxo (ip; 2 mg/kg)** | **248** |  |
| **PET-CT (7)** | **269** | **Exophthalmos regression and reduction of PET activity** |
| **Additional weekly treatment with doxo**  **(1 mg/kg/week; injected ip twice per week)** | **276** |  |
| **PET-CT (8)** | **302** | **Reduction of the number of nodules detected and PET activity** |
| **PET-CT (9)** | **330** | **Reduced PET activity** |
| **Doxo treatment discontinued** | **338** |  |
| **PET-CT (10)** | **385** |  |
| **T12 dose lowered (0.01 mg/mL )** | **456** |  |

| **Mouse-2** | **Days after 4T1 inoculation** | **Observations** |
| --- | --- | --- |
| **Birth** | **-76** |  |
| **Inoculation of 4T1 cells** | **0** |  |
| **Beginning of T12 treatment (0.5 mg/mL)** | **13** |  |
| **Mastectomy** | **16** |  |
| **PET-CT (1)** | **70** | **Metastatic nodules detected** |
| **PET-CT (2)** | **85** | **Maintained PET activity** |
| **T12 dose reduced (0.1 mg/mL)** | **85** |  |
| **PET-CT (3)** | **139** |  |
| **T12 treatment discontinued** | **140** |  |
| **PET-CT (4)** | **160** | **An additional nodule detected** |
| **PET-CT (5)** | **183** |  |
| **T12 treatment restarted (0.1 mg/mL)** | **184** |  |
| **Additional weekly treatment with doxo**  **(1 mg/kg/week; injected ip twice per week)** | **190** |  |
| **PET-CT (6)** | **216** | **The additional nodule is not detected** |
| **PET-CT (7)** | **244** | **Reduced PET activity** |
| **Doxo treatment discontinued** | **252** |  |
| **PET-CT (8)** | **299** |  |
| **T12 dose lowered (0.01 mg/mL )** | **370** |  |

Columns (left to right): relevant interventions on mice; day referred to inoculation of cells; and observations related with preclinical progression.
